# Common and rare variant associations with latent traits underlying depression, bipolar disorder, and schizophrenia

**DOI:** 10.1038/s41398-023-02324-6

**Published:** 2023-02-06

**Authors:** Saloni Dattani, Pak C. Sham, Bradley S. Jermy, Jonathan R. I. Coleman, David M. Howard, Cathryn M. Lewis

**Affiliations:** 1 Social, Genetic and Developmental Psychiatry Centre, Institute of Psychiatry, Psychology & Neuroscience, King’s College London, London, UK; 2 Department of Psychiatry, Li Ka Shing (LKS) Faculty of Medicine, University of Hong Kong, Hong Kong SAR, China; 3 NIHR Maudsley Biomedical Research Centre, South London and Maudsley NHS Trust, London, UK; 4 Department of Psychiatry, State Key Laboratory of Brain and Cognitive Sciences, The University of Hong Kong, Hong Kong SAR, China; 5 Centre for PanorOmic Sciences, Li Ka Shing Faculty of Medicine, The University of Hong Kong, Hong Kong SAR, China; 6 Division of Psychiatry, University of Edinburgh, Royal Edinburgh Hospital, Edinburgh, UK; 7 Department of Medical and Molecular Genetics, Faculty of Life Sciences and Medicine, King’s College London, London, UK

**Keywords:** Clinical genetics, Diagnostic markers, Psychiatric disorders

## Abstract

Genetic studies in psychiatry have primarily focused on the effects of common genetic variants, but few have investigated the role of rare genetic variants, particularly for major depression. In order to explore the role of rare variants in the gap between estimates of single nucleotide polymorphism (SNP) heritability and twin study heritability, we examined the contribution of common and rare genetic variants to latent traits underlying psychiatric disorders using high-quality imputed genotype data from the UK Biobank. Using a pre-registered analysis, we used items from the UK Biobank Mental Health Questionnaire relevant to three psychiatric disorders: major depression (*N* = 134,463), bipolar disorder (*N* = 117,376) and schizophrenia (*N* = 130,013) and identified a general hierarchical factor for each that described participants’ responses. We calculated participants’ scores on these latent traits and conducted single-variant genetic association testing (MAF > 0.05%), gene-based burden testing and pathway association testing associations with these latent traits. We tested for enrichment of rare variants (MAF 0.05–1%) in genes that had been previously identified by common variant genome-wide association studies, and genes previously associated with Mendelian disorders having relevant symptoms. We found moderate genetic correlations between the latent traits in our study and case–control phenotypes in previous genome-wide association studies, and identified one common genetic variant (rs72657988, minor allele frequency = 8.23%, *p* = 1.01 × 10^−9^) associated with the general factor of schizophrenia, but no other single variants, genes or pathways passed significance thresholds in this analysis, and we did not find enrichment in previously identified genes.

## Introduction

Psychiatric disorders, such as major depression, schizophrenia and bipolar disorder, are devastating conditions that disrupt individuals’ normal functioning. They are also heritable, meaning that a proportion of the variance of the predisposition to these disorders is attributable to genetic variation. The heritability of these disorders can be estimated from twin and family-based studies; for example, the heritability of major depression has been estimated as 32% from national registries in Denmark, while schizophrenia and bipolar disorder have estimates of 67 and 64%, respectively [1, 2].

Several large-scale genome-wide association studies (GWAS) have identified common genetic variants associated with these disorders [3–6]. However, when these associations are aggregated, the estimated heritability from common variants is substantially lower than the estimates of heritability from twin and family-based studies: while the heritability estimate of bipolar disorder from twin studies is around 64%, the estimate using common single-nucleotide polymorphisms (SNPs) from GWAS is 17–23% [6].

One explanation for the discrepancy between heritability estimated from twin studies and from SNPs is the focus of the latter on *common* genetic variation (present in ≥1% of the population) while largely ignoring the contribution of rare genetic variants [7]. For example, where whole genome sequencing data was used to estimate the total contribution of both rare and common variants to height, heritability estimates from measured genetic variants were consistent with those from family-based studies [8].

In psychiatry, few studies have focused on rare genetic variants associated with major depression [9, 10], while several have investigated schizophrenia and bipolar disorder [11–13]. Studies of some psychiatric disorders including schizophrenia have found an enrichment of ultra-rare disruptive variants [11, 14, 15] and studies of common variants for complex diseases have found enrichment of genes associated with matched Mendelian disorders [16].

The high cost of genome and exome sequencing has likely impacted the rate of progress for rare variant analysis. Sample sizes with whole genome or exome sequence data have been relatively small compared to those with SNP genotyping array data. Recently, however, studies have increased in their sample sizes [17–19]. Also, consortia of researchers have aggregated large panels of individuals with whole genome sequence data, which can be used as reference samples to accurately impute variants that have not been directly genotyped. This provides an additional method for researchers to increase the number of genetic variants that can be tested without significant cost.

In addition, there is growing interest and recognition of subclinical symptoms in the general population, especially among those with a family history of psychiatric disorders, and those who may not seek treatment or be diagnosed [20].

Studies suggest that depressive symptoms lay on a continuum and that dichotomisation results in a loss of information [21, 22], although researchers often use a sum score model to categorise individuals as cases or controls depending on whether the sum of their responses on a questionnaire falls above or below a certain threshold. The assumptions underlying the sum score model—such as that items have equivalent loadings and residuals on the underlying factor—may be violated for symptoms of these disorders [23], and simulations have shown that these violations reduce statistical power to detect associated variants [24].

In this study, we investigated common and rare genetic variants associated with latent psychiatric traits found in the population, using high-confidence imputed genotype data from the UK Biobank. The Mental Health Questionnaire of the UK Biobank contains symptom-level data for >150,000 participants related to various psychiatric disorders, which makes it a rich source of information on the distributions of these symptoms and correlations between them. Using symptom-level data of psychiatric traits, we extracted participant-level scores on continuous latent factors using factor analysis.

We identified items in the Mental Health Questionnaire that matched criteria in the DSM-V and ICD-10 diagnoses of major depression, schizophrenia and bipolar disorder, and used factor analysis to construct latent variable models for each condition. With these, we calculated individual scores for the latent psychiatric traits, and investigated genetic variants and genes across the allele frequency spectrum associated with them.

Furthermore, we explored the relationship between the latent psychiatric traits and Mendelian disorders exhibiting similar symptoms, by testing whether genes associated with matched Mendelian disorders were enriched for genetic associations with the latent psychiatric traits. We also investigated whether rare genetic variants associated with the latent psychiatric traits were colocalised in genes that were previously identified by case-control studies of the psychiatric disorders that used common variant analysis. Finally, we tested whether rare genetic variants associated with these latent psychiatric traits had larger effect sizes than common genetic variants.

## Methods

**Fig. 1:**
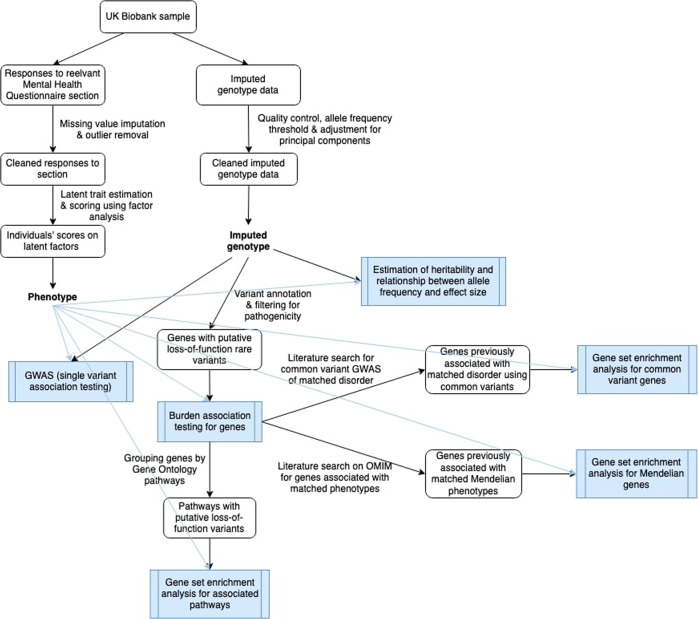
Flowchart depicting methods used in this paper. Shaded boxes represent statistical analyses reported in the results of this paper.

A flowchart summarising the methods is presented in Fig. 1.

### Sample

We used data from the Mental Health Questionnaire (MHQ) in the UK Biobank to investigate latent traits underlying psychiatric symptoms, as this questionnaire contains items relating to current and lifetime symptoms of psychiatric disorders that match the DSM-V and ICD-10 criteria.

The MHQ was a follow-up study of participants that were initially recruited for the UK Biobank between 2006–2010 and aged 40–69 years [25, 26]. One hundred and fifty-seven thousand five hundred and thirty-eight participants completed the questionnaire and we conducted power calculations to ascertain that we had high power to detect the smallest effect sizes reported in the literature (Tables S1 and S2).

### Latent trait construction

We identified MHQ items (supplementary material) matching the DSM-5 and ICD-10 criteria for schizophrenia, bipolar disorder and major depression. For major depression (pilot analysis), we used the items and five factor model in Jermy et al. [27]. Items related to mania (for schizophrenia and bipolar disorder) were asked as follow-ups (symptoms experienced during a period of irritability or a period of feeling high, excited or hyper), so negative responses to these period questions were coded as negative responses to the follow-up items.

We imputed missing responses using their responses to other items with the regression imputation function *regressionImp()* in VIM [28] and removed participants with any remaining missing responses, as described in the supplement. We removed participants who scored >99th percentile on the multivariate Robust Mahalanobis Distance score [29, 30] as outliers in combinations of their responses (except for schizophrenia, due to the low prevalence of affirmative responses to some items). This resulted in sample sizes of 153,693, 134,249 and 148,681 for major depression, bipolar disorder, and schizophrenia respectively (Table S6 and Figs. S1–S11).

We split the data into two random subsamples of participants to perform exploratory factor analysis and confirmatory factor analysis in either half. We used weighted least squares factoring and geominQ (olique) rotation to fit models using different numbers of latent factors [31], compared them using fit statistics and used Thurstone’s rules to adjust item retention (Figs. S12–S17). In confirmatory factor analysis, we estimated fit statistics to validate chosen models and we specified a hierarchical general factor to account for correlations between latent factors and computed fit statistics for hierarchical models (Figs. S18–S20).

Finally, we calculated individual-level scores using the empirical Bayes method with lavPredict() in lavaan for R [32]. Scores on the hierarchical general factors were used as phenotypes for all genetic analyses (Figs. S21–S23). These showed moderate genetic correlations with matched case–control phenotypes in published GWAS (Table 1).

**Table 1:** Heritability and genetic correlation estimation from using high-definition likelihood inference (HDL).

	*N*_eff_	*h*^2^ (SE)	Genetic correlation (SE)	*p*
Depression (Wray et al. 2018^a^ [3])	111,221	0.081 (0.004)	0.68 (0.05)	1.43 × 10^−40^
Internalising factor (Int)	74,663	0.113 (0.007)		
Schizophrenia (PGC 3 SCZ, 2020)	157,013	0.291 (0.009)	0.24 (0.03)	6.34 × 10^−14^
General factor of schizophrenia (Sch)	74,087	0.111 (0.006)		
Bipolar disorder (PGC 3 BD, 2020)	101,962	0.274 (0.010)	0.40 (0.04)	2.62 × 10^−26^
General factor of bipolar disorder (Bip)	65,709	0.103 (0.006)		

Results of LD-derived heritability and genetic correlation analysis of latent traits in this study and case control analyses from matched GWAS studies, using the high-definition likelihood inference software (HDL). Minimum MAF = 0.05%. *N*_eff_ = effective sample size (Table S22), *h*^2^ *=* heritability, SE = standard error. For matched GWAS studies, we estimated heritability with HDL using summary statistics from the original papers.

^a^For Wray et al. (2018 [3]), we used summary statistics from PGC (i.e. excluding UK Biobank and 23andMe data).

### Genotype data quality control

DNA were extracted from samples collected during recruitment and genotyped in 106 batches across assessment centres and aligned to build GRCh37. Genotype data underwent quality control prior to release by the UK Biobank [25]. We used imputed genotype data provided by the UK Biobank (supplementary methods).

We applied a maximum missing genotype filter of <0.02 for variants and <0.02 for participants, Hardy–Weinberg Equilibrium threshold of *p* < 1 × 10^−8^ and removed gender/sex mismatches. Participants’ relatedness was estimated prior to release by the UK Biobank, using the KING software [33]. We removed participants with a relatedness >0.044 with others using a greedy algorithm [34].

Rare variant analysis is highly sensitive to population stratification, therefore we restricted participants to genetically-inferred European ancestry, using four-means clustering on the first two genetic principal components (PCs) derived from the UK Biobank [35]. Out of 502,620 UK Biobank participants with genotype data, 116,961 were removed during genotype QC and 5 excluded due to missing covariates (Tables S6 and S7).

The final sample size was 134,463, 130,013, and 117,376 for the general factors of depression, schizophrenia and bipolar disorder, respectively.

Phenotype scores were adjusted for the first 20 genetic PCs, their collection centre and genotype batch, using linear regression. PCs were calculated from quality-controlled genotype data using FlashPCA2 with no MAF threshold, a window size of 1500 SNPs, a window shift of 150 SNPs, and a linkage disequilibrium threshold of *r*^2^ > 0.02 for pruning [36].

We excluded variants with INFO < 0.7 [37], converted gene dosages to hard-called genotypes with a threshold of >0.9, and excluded variants with MAF < 0.05% for GWAS and burden tests, and MAF < 0.005% for heritability estimation, following recommendations by Wright et al. [38].

### Association testing

We conducted genome-wide association testing for single variants including common variants (MAF > 0.05%), by regressing genotypes on PC-adjusted latent traits with the fastGWA software [39] and plotted results using qqman and ggManhattan [40, 41], using the significance threshold of 5 × 10^−9^, recommended by Wu et al. for imputed genotype data [42]. Since we processed phenotypes separately, 13,921,407, 13,933,876 and 13,911,892 single variants were tested for the general factors of depression, schizophrenia and bipolar disorder respectively (Tables S8 and S9).

We conducted gene-based burden testing using only rare (MAF 0.05–1%) variants predicted to be deleterious (as described below) with the MAGMA software [43], which uses multiple linear principal components regression, to regress participants’ scores on latent traits and calculate empirical *p* values using an *F*-test with 1000 fixed permutations. We applied Bonferroni correction to adjust for the number of genes tested, for a significance threshold of *p* = 4.2 × 10^−5^.

Using the association statistics from gene-based burden testing, we also tested Gene Ontology pathways (all GO pathways in C5v6 using Entrez gene definitions) [44] using MAGMA, with competitive gene-set enrichment analysis, along with Bonferroni correction for the number of gene sets tested.

Genes were defined from the NCBI transcription start to stop site, for protein-coding genes; these were listed on the MAGMA repository, updated September 2018 [45]. Variants were annotated with ANNOVAR using ensGene [46] and dbNSFP33a [47] (to predict the functional impact of variants with dbscSNV [48], MutationTaster [49], GERP++ [50], FATHMM [51], and SIFT [52]) and we considered them predicted deleterious if they passed thresholds recommended by authors (supplementary methods).

### Enrichment analysis

Using the association statistics from gene-based burden testing, we grouped all matched genes (described below) as a gene set and performed competitive gene-set enrichment analysis using MAGMA to test for an enrichment of predicted deleterious variants in these genes.

#### Matched Mendelian disorders

To identify loci linked to Mendelian disorders exhibiting relevant clinical features, we conducted an advanced search on the Online Mendelian Inheritance in Man database (OMIM) [53]. We searched for clinical features of depression (for major depression), schizophrenia or psychosis (for schizophrenia), and bipolar disorder or mania (for bipolar disorder) (supplementary methods). We manually filtered search results to relevant phenotypes and restricted associated loci to those that contained <5 genes each (Fig. S24).

#### Matched common variant GWAS

We used significantly associated genes from gene-based burden testing from the three largest GWAS of matched psychiatric illnesses: Wray et al. [3] for major depression, with UK Biobank data excluded and the analysis restricted to variants with a MAF > 1% (58 genes); the Mullins et al. [54] of 40,000 cases for bipolar disorder (126 genes); and Ripke et al. [55] of 69,000 cases for schizophrenia (360 genes). These can be found in our repository: https://osf.io/w8jyu/.

### Heritability and genetic correlations

In post hoc analysis, we used the HDL software [56] (v1.3.9) to estimate LD-derived heritability, and genetic correlations between latent traits and case-control phenotypes from matched GWAS [3, 54, 57] using genome-wide summary statistics. We used 1,029,876 QCed UK Biobank imputed HapMap3 SNPs as the LD reference panel [58].

We also used the openMPI parallelised version of GCTB [59] v2.0 to estimate polygenicity and the relationship between allele frequency and effect size (*S*, also an indicator of selection). We analysed only chromosome 1 for computational feasibility; the distribution of *S* (selection coefficient) and pi (polygenicity) are not expected to vary between chromosomes and are not additive. We used the nested BayesC model, which analyses non-overlapping genomic regions as windows and skips over windows with zero effect sizes. We specified a nested window size of 1 Mb, with starting values pi = 0.05, *h*^2^ = 0.05 and *S* = 0, and a MCMC chain length of 10,000 and burn-in of 2000, as recommended by Zeng et al. [59].

## Results

### Phenotype creation

One hundred and fifty-seven thousand three hundred and sixty-sixparticipants who responded to the Mental Health Questionnaire of the UK Biobank (accessed in November 2020) were used to construct latent factor phenotypes for subsequent analyses. Data processing was conducted separately for each disorder. After imputation of missing values and outlier removal, we performed exploratory factor analysis, with 153,693 participants retained for the phenotype of major depression, 134,249 for bipolar disorder and 148,681 for schizophrenia.

#### Latent factor models

For depression, the model matching that constructed in Jermy et al. [27] was used. Five factors were identified and labelled anxiety, psychomotor, neurovegetative, mood and reflective symptoms. A general hierarchical factor (called the internalising factor) was fitted, which loaded highly onto each of the five lower factors (>0.7). In the hierarchical model constructed with depression-related items, measures indicated an acceptable model fit (omega Total=0.9, ECV = 0.46, CFI = 0.992, TLI = 0.990, RMSEA = 0.039 and SRMR = 0.031).

For schizophrenia, three factors were identified, labelled psychotic, negative and disorganised symptoms. A general hierarchical factor was fit, which had moderate to high loadings on each of the three lower factors (>0.6). In the hierarchical model constructed with schizophrenia-related items, measures indicated an acceptable model fit (omega Total=0.88, ECV = 0.41, CFI = 0.996, TLI = 0.995, RMSEA = 0.018 and SRMR = 0.048).

For bipolar disorder, three factors were identified, labelled depressive, manic and disorganised symptoms. A general hierarchical factor was fit, which loaded moderately onto each of the three lower factors (>0.5). In the hierarchical model constructed with bipolar disorder-related items, measures indicated an acceptable model fit (omega total = 0.93, ECV = 0.73, CFI = 0.999, TLI = 0.999, RMSEA = 0.023 and SRMR = 0.044). The final model solutions are shown in Figs. S18–S20.

### Association testing

#### Single variant association tests

Single variant association tests were conducted with a MAF > 0.05% using linear regression adjusted for the first 20 principal components, with the fastGWA software. The genome-wide significance threshold of *p* < 5 × 10^−9^ was used to identify single variants associated with latent traits.

**Fig. 2:**
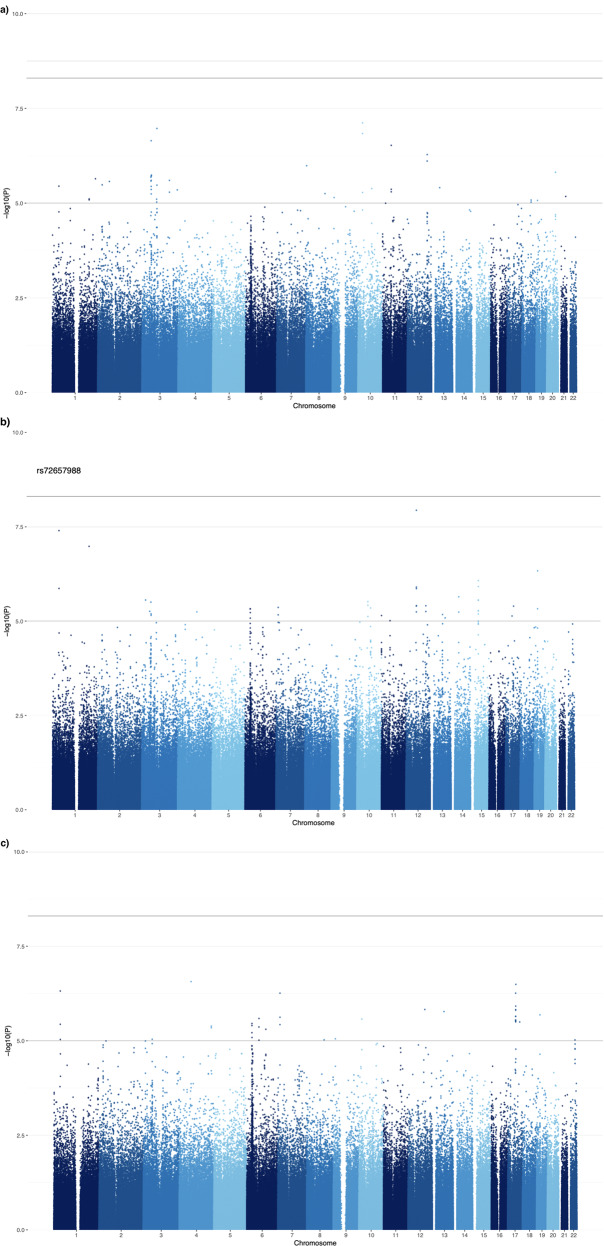
Manhattan plot for single variants. Panels depict the –log_10_(*p*-value) of association test statistics for single variants with **a** the internalising factor of depression (Int), **b** general factor of schizophrenia (Sch), and **c** general factor of bipolar disorder (Bip). Points on this plot represent individual genetic variants. The horizontal axis depicts the genomic coordinate of variants tested for an association with the internalising factor of depression. Minimum MAF = 0.05%. The solid horizontal line (at *p* = 5 x10^–9^) depicts the threshold for genome-wide statistically significant associations, while the light horizontal line (at *p* = 1 x 10^–5^) depicts a more lenient threshold above which variant rsID numbers are labelled.

For the internalising general factor of depression and the general factor of bipolar disorder, no single variants passed the significance threshold. One common SNP was associated with a general factor of schizophrenia above the genome-wide significance level: rs72657988, located on chromosome 1 (MAF = 0.0823, *p* = 1.01 × 10^−9^). This SNP was in linkage disequilibrium with two other SNPs that were nominally significant rs78201023 (MAF = 0.0393, *p* = 4.01 × 10^−8^, *D*’ = 0.897) and rs77512118 (MAF = 0.0372, *p* = 1.37 × 10^−6^, *D*’ = 0.908). In a recent meta-analysis, rs72657988 was identified to be associated with post-traumatic stress disorder (*p* = 1.2 × 10^−10^) [60]. Figure 2a–c display the results of the single variant association tests as Manhattan plots.

We calculated that the genomic inflation factor was 1.09 for the general factors of depression and schizophrenia and 1.08 for the general factor of bipolar disorder. We also calculated that the LDSC intercept was 1.0045, 1.0074 and 1.0073 for the three factors respectively (Table S13) which suggests the inflation arises from polygenicity rather than population stratification. QQ-plots are provided in Supplementary Figs. S26–S28 and indicate that genomic inflation predominately occurs for common variants (MAF > 0.05). Summary statistics from these analyses are available in supplementary materials (Tables S10–S12) and available in full online at https://osf.io/w8jyu/.

### Functional prediction

Twenty-one thousand two hundred and forty-four autosomal variants were considered predicted deleterious variants, either by being annotated as protein-truncating (*N* = 3 774) deleterious missense or indel (*N* = 14 099), deleterious splicing (*N* = 3 341), or deleterious non-coding (*N* = 30) variants (Tables S14–S16 and Fig. S29).

#### Gene-based burden tests

**Fig. 3:**
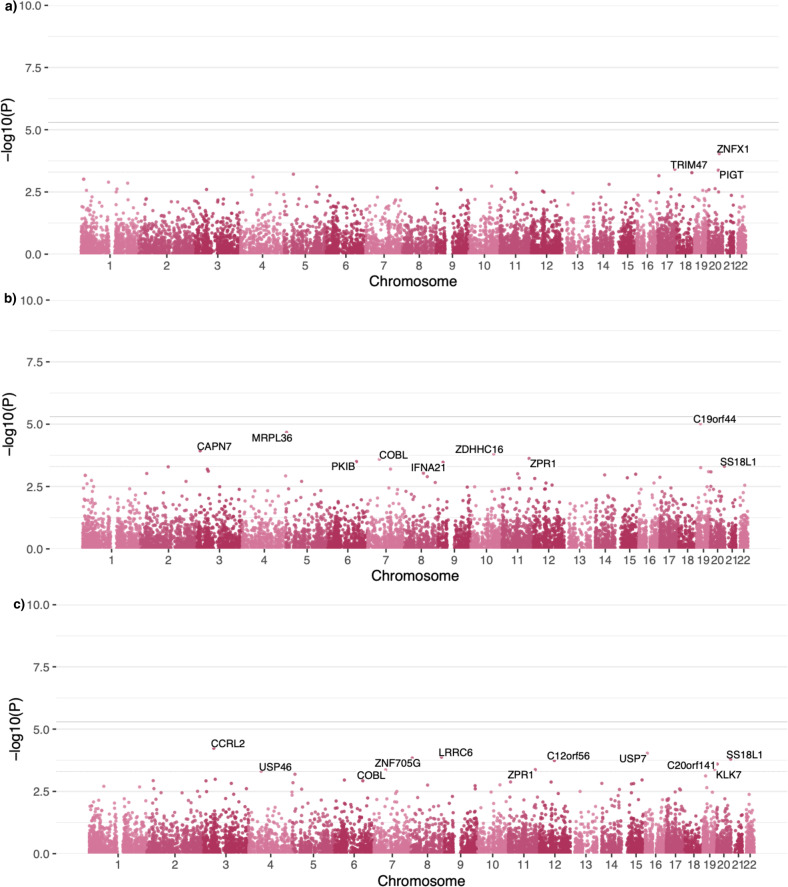
Manhattan plot for genes. Panels depict the –log_10_(p-value) of association test statistics for genes using gene burden testing with **a** the internalising factor of depression (Int), b the general factor of schizophrenia (Sch), **c** the general factor of bipolar disorder (Bip). Points on this plot represent individual genes with predicted deleterious variants. The horizontal axis depicts the genomic coordinate of genes tested for an association with the general factor of schizophrenia. Predicted deleterious variants within the MAF range between 0.05% and 1% were retained for gene burden testing. The solid horizontal line (at *p* = 4.2 x 10^–5^) depicts the threshold for genome-wide statistically significant associations after Bonferroni correction for number of tests performed. The dashed line (at *p*< 1 x 10^–3^) depicts a more lenient threshold above which genes have been annotated.

A median of three rare (MAF 0.05–1%) predicted deleterious variants were found per gene tested in gene-burden analyses (Fig. S30). Since we processed samples for each trait separately, 9964, 9912 and 9954 genes were tested for the general factors of depression, bipolar disorder and schizophrenia respectively (Table S8). No gene met the significance threshold of *p* = 4.2 × 10^−5^ (using Bonferroni correction for the number of tests performed) for any of the three latent traits (Fig. 3a–c). Summary statistics for this analysis are available in Tables S17–S19 and available in full online at https://osf.io/w8jyu/.

We estimated the genomic inflation factor from results of the burden analyses: 1.01 for the internalising factor of depression, 1.04 for the general factor of schizophrenia and 1.02 for the general factor of bipolar disorder. QQ-plots are provided in Fig. S31a–c.

### Enrichment analyses

#### Matched common variant genes

There was no enrichment for rare (MAF 0.05–1%) predicted deleterious variants in genes implicated by common variants previously associated with matched disorders [3, 54, 55] in any of the latent psychiatric traits in our study (internalising factor with GWAS of major depression, 58 genes, *p* = 0.59; general factor of schizophrenia with GWAS of schizophrenia, 360 genes, *p* = 0.33; general factor of bipolar disorder with GWAS of bipolar disorder, 126 genes, *p* = 0.16), see Table 2.

**Table 2:** Gene set enrichment analysis for GWAS results and Mendelian genes from OMIM.

	Set tested for enrichment	*N* genes	Beta	SE	*p*
	GWAS				
General factor of bipolar disorder (Bip)	Mullins et al. (PGC)	126	0.09	0.093	0.16
General factor of schizophrenia (Sch)	Ripke et al. (PGC)	360	0.02	0.055	0.33
Internalising factor of depression (Int)	Wray et al. (only PGC & 23andMe data)	58	−0.03	0.150	0.59
	OMIM				
General factor of bipolar disorder (Bip)	Depress* OR mania OR manic	169	−0.07	0.078	0.81
General factor of schizophrenia (Sch)	Schiz* OR psychotic OR psychosis	54	0.20	0.137	0.08
Internalising factor of depression (Int)	Depress*	26	0.11	0.198	0.28

Summary statistics to show results of gene set enrichment analysis of predicted deleterious variants in genes previously associated with matched disorders in GWAS and in genes previously associated with matched phenotypes of Mendelian disorders on OMIM. Minimum MAF = 0.05%, maximum MAF = 1%. *N* genes = number of genes included in the gene set that was tested for enrichment, Beta = regression coefficient, SE = standard error of the regression coefficient.

The asterisk represents the wildcard used for searching for alternative endings of the word, such as “depressive” and “depression”, or “schizophrenia” and “schizophrenic”.

#### Matched Mendelian disorders

We identified loci associated with matched clinical features from Mendelian disorders on OMIM (26 loci for depression, 54 loci for schizophrenia and 169 loci for bipolar disorder), as shown in Table 2. Focusing on rare (MAF 0.05–1%) predicted deleterious variants, there was no enrichment for any of the three sets of common variants previously associated with matched disorders (internalising factor with Mendelian disorders with depressive phenotypes, *p* = 0.81; general factor of schizophrenia with Mendelian disorders with schizophrenia phenotypes, *p* = 0.08; general factor of bipolar disorder with Mendelian disorders with bipolar disorder phenotypes, *p* = 0.28).

#### Gene Ontology pathways

Five thousand nine hundred and sixteen GO pathways were tested, resulting in a Bonferroni-corrected significance threshold of 8.45 × 10^−6^. No GO pathways passed significance thresholds after Bonferroni correction. Full summary statistics are available in Tables S19–21.

### Heritability and genetic correlations

We used the HDL software [56] in post-hoc analysis to estimate LD-derived heritability for each latent trait and to estimate genetic correlations between these traits and the phenotypes measured in case-control analyses from the common variant GWAS studies used for enrichment analysis.

Heritability was estimated using HDL at 0.113 for the internalising factor of depression, 0.111 for the general factor of schizophrenia and 0.103 for the general factor of bipolar disorder. For case–control phenotypes from common variant GWAS studies, heritabilities were estimated at 0.081 for depression in Wray et al. [3], 0.291 for schizophrenia in Ripke et al. [61] and 0.274 for bipolar disorder in Mullins et al. [54].

Genetic correlations between each pair of traits were moderate in magnitude and were all highly significant (*p* < 9.14 × 10^−14^ for Levene’s test for variance heterogeneity; Tables 1 and S22), indicating that the continua of latent traits estimated from the population in this study shared genetic contribution with phenotypes used in case-control analyses.

We estimated the polygenicity to be 0.045 and 0.051, and the selection coefficient to be 7.8 × 10^−5^ and 6.8 × 10^−5^ for the general factor of bipolar disorder and of schizophrenia respectively, using data from chromosome 1 and a MAF threshold of >0.005%, while the MCMC model for the internalising factor did not converge. Figure S33 and Table S24 illustrates the estimates and distributions of heritability, polygenicity and selection coefficient across 10 000 iterations of the MCMC model.

## Discussion

This study explored the contribution of common and rare variants to latent factors derived from symptoms of major depression, schizophrenia and bipolar disorder in the UK Biobank. The Mental Health Questionnaire contains self-report questions from the CIDI-SF questionnaire which asks sufficient questions for a clinical diagnosis according to the ICD-10 and DSM-5, which makes it a particularly valuable source of data regarding the distribution of psychiatric traits in the wider population and their relation to clinical phenotypes.

We found moderate genetic correlations between the latent traits we derived from reported symptoms and case-control phenotypes from previous studies. We noted that, for schizophrenia, the genetic correlation between the latent trait and the case-control phenotype was low, while it was moderate for bipolar disorder and much higher for depression (Table 1). We found high genetic correlations between all three latent traits and the case-control phenotype of depression (Table S23). Previous studies [62, 63] have also found higher genetic correlations between psychotic experiences and depression than schizophrenia, although the mechanisms are unclear.

Additionally, heritability estimates were lower in latent traits in the UK Biobank compared to case-control phenotypes from previous GWAS (Table 1). This may be partly due to the characteristics of the sample, as participants in the UK Biobank and Mental Health Questionnaire demonstrate ‘healthy volunteer bias’—where participants tend to be healthier and more educated than the wider population—and may have lower liability to psychiatric illness, which may result in lower estimates of heritability and genetic correlations [64]. The prevalence of self-reported diagnoses of mental illness including depression has been reported to be similar in the Mental Health Questionnaire and representative surveys of the general population of the same age group [26, 65]. However, these comparisons have more uncertainty for bipolar disorder and psychotic disorders due to their low prevalence rates and small numbers in the survey data, and it is likely that those with concurrent severe symptoms were less likely to participate [26]. The challenges associated with voluntary recruitment for cohort studies including the UK Biobank imply that our findings may be more informative about milder symptoms in the wider population than those with severe mental illness.

In genome-wide association analysis, we identified one common SNP associated with a general factor of schizophrenia: rs72657988 (MAF = 0.0823, *p* = 1.01 × 10^−9^). This SNP was in linkage disequilibrium with two other SNPs that were nominally significant, none of which had previously been associated with schizophrenia, and had been identified as a statistically significant association in a recent meta-analysis of post-traumatic stress disorder [60]. It will be important to validate the consequences of this SNP in functional studies. No SNPs were associated with the general factor of bipolar disorder or the general internalising factor of major depression.

No genes were associated with the three latent factors in gene-based burden testing of rare predicted deleterious variants. Similarly, no GO pathways were associated with the three latent factors.

Although power calculations indicated this study had sufficient power (95% power to detect SNP associations with a QTL variance of >0.00038 and genes with >0.1% explained variance (Tables S1 and S2), the lack of findings suggests that rare variants remain challenging to capture using large, imputed genotype data sets and their effect sizes may be smaller than anticipated.

### Enrichment in Mendelian genes

Gene-based burden testing was used to examine enrichment of predicted deleterious variants in genes previously associated with Mendelian disorders where patients developed matched clinical features. For example, are predicted deleterious variants associated with the latent factor of schizophrenia more likely to be found in genes associated with Mendelian disorders where patients developed schizophrenia?

Enrichment may occur in patients at distribution extremes for psychiatric symptoms who develop those traits due to single fully penetrant Mendelian variants. However, research has failed to identify fully penetrant single variants for psychiatric disorders [66, 67]. Several rare copy number variants that confer substantial risk to schizophrenia and to bipolar disorder have been identified, but they are not fully penetrant [68, 69]. Previous research has also found an increased burden of missense and loss-of-function de novo variants in schizophrenia and bipolar disorder [70, 71], suggesting that their genetic component includes both rare variants with large effects and common variants with small effects.

Genes associated with Mendelian traits may also contain common variants associated with the broader phenotype of a trait. This would indicate that complex phenotypes and Mendelian traits with matched phenotypes have a common aetiology, resulting from the functions of genes containing such variants. For example, Freund et al. [16] found an enrichment of genes associated with Mendelian traits in GWAS of various matched complex disorders. Similarly, Blair et al. [72] also found comorbid associations between Mendelian disorders and complex traits, including between Marfan syndrome and psychiatric illnesses, and between psychiatric illnesses and four genes associated with Mendelian disorders (SYNE1, PRPF3, CACNA1C and PPP2R2B).

Since Mendelian variants are often associated with syndromes that cause a variety of symptoms and phenotypes, it is believed that complex traits may result from variation in genes that have pleiotropic effects [73]. However, we found no enrichment of Mendelian genes of matched phenotypes for any of the three latent psychiatric traits in this study.

There are several potential reasons for not observing enrichment. Firstly, the genes we identified through OMIM may have been coincidentally associated with matched phenotypes. For example, we searched for keywords related to psychiatric disorders in the clinical features section of entries of Mendelian disorders. However, in some cases, patients who developed those Mendelian disorders may have also developed depression and mania from unrelated causes but would still lead to an inclusion of associated Mendelian genes into our gene sets. Secondly, the study may lack the statistical power necessary to detect an enrichment in these genes for a large population with symptom-level data. Thirdly, rare variants of large effects may be missed in imputed genotype data due to limited haplotypes in reference panels.

### Enrichment of genes identified by GWAS

We examined the shared genetic aetiology between common and rare variants associated with psychiatric illnesses, by testing for the enrichment of rare predicted deleterious variants in genes previously associated in GWAS of common variants for matched psychiatric disorders as the latent traits in this study. However, we found no evidence for enrichment.

Previous research has identified an enrichment of rare variants in genes associated with psychiatric disorders. For schizophrenia, several studies [11, 74] have found enrichment of ultra-rare variants in genes implicated by schizophrenia GWAS of common variants, with some research focusing on disruptive variants in particular [75].

Research examining enrichment in bipolar disorder have been more mixed, with some research finding enrichment of rare variants in genes previously implicated by bipolar disorder GWAS, while other studies have not found evidence for enrichment [76, 77]. Few studies have examined rare variant associations with major depression [10] or their relationship with genes implicated by common variant GWAS.

Unlike other literature, this study did not use sequence or exome array data due to a lack of availability for large symptom-level data, but the UK Biobank Axiom array also covers variation in exonic regions and areas of rare coding variation including protein-truncating variants [78]. The lower coverage of these genomic regions means the results of this study are not informative about the contribution of ultra-rare, de novo and private mutations, which have been implicated in previous studies of psychiatric illness [11, 15, 79].

### Relationship between allele frequency and effect size

This study also investigated the relationship between allele frequency and effect size for latent traits and found a very weak relationship (sigma of 7.8 × 10^−5^ and 6.8 × 10^−5^ for the general factors of bipolar disorder and schizophrenia, respectively).

In contrast, Zeng et al. [59] found that many complex traits were under significant negative selection as measured by the selection coefficient (relationship between allele frequency and effect size) using the software GCTB. Our finding of a virtually zero relationship between SNP allele frequency and effect size for these latent traits may indicate they are not under selection or that, as many rare variants will be recent, there has been insufficient time for selection to reduce their frequency in the population.

While much of the literature reports larger effect sizes from rare variants than common variants, the focus has primarily been on structural variants and single nucleotide variants predicted to be highly damaging [11, 66]. Singh et al. [11] found that common SNPs have smaller effect sizes than rare CNVs and protein-truncating variants for schizophrenia using exome sequence data. This may in part be because CNV and protein-truncating variants are expected to affect gene function more considerably than SNPs.

### Limitations

There are several limitations when analysing rare variants, which we sought to minimise. Firstly, imputation accuracy is lower for rare variants due to the limited number of reference haplotypes containing those rare variants. We followed recommendations by Pistis et al. [37] and restricted variants to those with an INFO score above 0.7 and additionally restricted the minimum MAF of variants in this study according to recommendations from Wright et al. [38], to reduce type 1 errors.

Secondly, it is challenging to adjust rare variants for confounding by geographical stratification than common variants, because their spatial distributions are more complex and segmented in populations [80], although this limitation is less severe in gene-burden analyses as the averaging of multiple variants within genes more closely resembles the spatial distribution of common variants [81]. To address this, we adjusted factor scores for latent traits for the first 20 principal components generated from genotype data (excluding imputed variants) across the allele frequency spectrum, including rare variants. However, additional analyses using exome- or genome-sequence data may shed further light on these findings.

Thirdly, it is important to externally validate the results of this study, particularly the significant hit of rs72657988 for the general factor of schizophrenia, using functional studies that may illuminate the direct impact of this variant on the phenotype.

Finally, our study focused on participants who completed the Mental Health Questionnaire in the UK Biobank, a cohort study in which participants tend to be older, more educated and healthy than the general population [25, 26], and our analysis was restricted to participants of European ancestry, meaning that the findings may not be generalisable to the wider global population.

Aside from these limitations, this study has important contributions to the literature on genetic psychiatry. To our knowledge, this is the first study that jointly investigates the contribution of rare and common variants associated with a depression phenotype or that tests for enrichment of predicted deleterious variants in genes implicated by GWAS of depression. It is also the first to investigate the enrichment of Mendelian disease genes with matched clinical symptoms for depression—previous research had tested for enrichment in Mendelian disease genes that were associated with neurological disorders, rather than depressive symptoms specifically [16]. Finally, it is also one of few genetic studies that focuses on the continua of psychiatric traits in the wider population, rather than case-control status.

## Supplementary information

Supplementary materials

## Data Availability

We piloted the analysis using genotype data to test the method focusing on depression, and pre-registered the same analyses for imputed genotype data to test the methods for depression, schizophrenia and bipolar disorder. Summary data is available at https://osf.io/w8jyu/.

## Code availability

Code is available at https://osf.io/w8jyu/.
