# Supplementary material for: Common and rare variant associations with latent traits underlying depression, bipolar disorder, and schizophrenia
Source: Transl Psychiatry. 2023 Feb 6;13:46. doi: 10.1038/s41398-023-02324-6 (PMC9902570; doi:10.1038/s41398-023-02324-6)
Supplement: Supplementary file 1 — Supplementary materials [file 41398_2023_2324_MOESM1_ESM.pdf]

Supplementary materials for Common and rare variant associations with latent traits underlying depression, bipolar disorder, and schizophrenia

Authors: Saloni Dattani[1,2], Pak C Sham[3,4], Bradley S Jermy[1,5], Jonathan RI Coleman[1,5], David M Howard[1,6]*, Cathryn M Lewis[1,7]*

Affiliations:

1. Social, Genetic and Developmental Psychiatry Centre, Institute of Psychiatry, Psychology & Neuroscience, King's College London, London, UK
2. Department of Psychiatry, Li Ka Shing (LKS) Faculty of Medicine, University of Hong Kong, Hong Kong SAR, China
3. NIHR Maudsley Biomedical Research Centre, South London and Maudsley NHS Trust, London, UK
4. Social, Genetic and Developmental Psychiatry Centre, Institute of Psychiatry, Psychology & Neuroscience, King's College London, London, UK
5. NIHR Maudsley Biomedical Research Centre, South London and Maudsley NHS Trust, London, UK
6. Division of Psychiatry, University of Edinburgh, Royal Edinburgh Hospital, Edinburgh, UK
7. Department of Medical and Molecular Genetics, Faculty of Life Sciences and Medicine, King's College London, London, UK

Correspondence:
Saloni Dattani (first author and correspondence author), MSc.
Postal address: Social Genetic and Developmental Psychiatry Centre, Institute of Psychiatry, Psychology & Neuroscience, King's College London, London, UK
Email: saloni.dattani@kcl.ac.uk
Author contribution: Conceptualisation and design of the study, analysis, interpretation of data, and writing of manuscript

Pak C Sham, PhD
Author contribution: Interpretation of data and revision of manuscript

Bradley S Jermy, MSc:
Author contribution: Statistical analysis relating to major depression and revision of manuscript

Jonathan RI Coleman, PhD:
Author contribution: Genetic analysis, quality control and revision of manuscript

David M Howard* (senior author), PhD:
Author contribution: Design of the study, analysis, interpretation of data and revision of manuscript

Cathryn M Lewis* (senior author), PhD:
Author contribution: Design of the study, interpretation of data and revision of manuscript

* These two authors contributed equally to the manuscript

Supplementary code and results are also available at: https://osf.io/w8jyu/

POWER ANALYSES 4

PRE-REGISTRATION DETAILS AND DEVIATIONS 7

SUPPLEMENTARY METHODS 8

Phenotype creation 9
Factor analysis for latent trait estimation 9
Missing values 10
Outlier removal 10
Exploratory factor analysis 11
Confirmatory factor analysis 12
General factor solution 12
Factor scoring 12

Genotype data 13
Quality control 13
Imputation 14
Functional prediction of genetic variants 15
Heritability estimation 16

SUPPLEMENTARY RESULTS AND FIGURES 27

Raw responses to items 27
Depression phenotype 27
Schizophrenia phenotype 30
Bipolar disorder phenotype 33

Missing value imputation 36
Depression phenotype 36
Schizophrenia phenotype 37
Bipolar disorder phenotype 39

Outlier removal 40
Depression phenotype 40
Schizophrenia phenotype 41
Bipolar disorder phenotype 42

Exploratory factor analysis 43
Depression phenotype 43

Schizophrenia phenotype 45
Bipolar disorder phenotype 47

Confirmatory and hierarchical factor analysis 49
Depression phenotype 49
Schizophrenia phenotype 50
Bipolar disorder phenotype 52

Factor scoring 53
Depression phenotype 53
Schizophrenia phenotype 54
Bipolar disorder phenotype 55

Sample size and removals due to exclusions and genotype QC procedures 56

GWAS of single nucleotide variants 58
QQ plots 61
QQ plots stratified by MAF 63
Functional annotations of variants 65

Gene-based association testing 67

Heritability estimation and genetic correlations 81

Power analyses

Single variant association testing

We searched the published literature for genome-wide or exome-wide studies that tested individual rare variants for associations with schizophrenia and bipolar disorder. So far, published genome-wide association studies have tended to remove variants with an allele frequency lower than 1%. Those that have included rare variants have not found statistically significantly associations of individual rare (MAF <1%) single nucleotide polymorphisms with schizophrenia or bipolar disorder, although several exome-sequencing studies have used gene-based or pathway-based association testing to find associations between groups of rare pathogenic variants and these disorders [1–8].

Therefore, we used the lowest-frequency SNP that has been significantly associated with schizophrenia or bipolar disorder and estimate power for a range of effect sizes around that point estimate. The A allele at the SNP rs78089757 has an allele frequency of 0.0191 and was found to be associated with bipolar disorder with an odds ratio of 1.358 in a Chinese sample, which has replicated in a larger meta-analysis using a Japanese sample [9]. The proportion of trait variance, Var(X), that is attributable to this SNP is 0.0035085, according to the equation:

$$Var(X) = 2ß^2 \times f(1-f)$$

Where ß, ln(odds ratio), represents the effect size of the SNP, and f represents its minor allele frequency [10] and assumes zero non-additive variance. This equation is also written in the literature as $2pqa^2$ [11], where p is the minor allele frequency, q = 1 -p, and a is the effect size.

Power was estimated using the genetic power calculator [12]. We calculated the sample size required for 95% power, for a range of values for total variance explained at each locus (total QTL variance) between 0.0001 to 0.005. Furthermore, we estimated power using a model with no dominance, a marker allele frequency of 0.01, and a D-prime value of 1, since we are testing causal variants rather than those in linkage disequilibrium.

This indicated that the current sample size (approx. N = 150,000) would have >99% power to detect an association with a total QTL variance of the SNP rs78089757, and that the current sample size would have >95% power to detect associations with a total QTL variance above 0.00038, at the recommended type I error rate of $5x10^{-9}$ for imputed genotype data [13].

Table S1. Power with current sample size, and sample sizes required for 95% power, to detect associated single variants at a range of variances explained

Total QTL variance	Power with sample size of 1.5 x 10^5	Sample size required for 95% power
0.005	1	11,200
0.001	1	56,100
0.0005	0.9976	112,200
0.0004	0.9713	140,300
0.0003	0.8055	187,100
0.0002	0.3558	280,600
0.0001	0.02419	561,300

Gene-based association testing

We searched the literature for genome-wide or exome-wide association studies testing rare variants in gene-based tests for schizophrenia and bipolar disorder. Some studies of exome-sequencing data have identified genes associated with schizophrenia and bipolar disorder using collapsing tests, such as burden tests, including only those rare variants in each gene that are predicted to be damaging. For example, the *SETD1A* gene was significantly associated with schizophrenia at the whole-exome level in a meta analysis of cohorts involving European ancestries[7], and the *RBM12* gene was significantly associated with psychosis at the whole-genome level in an Icelandic sample and replicated in a Finnish sample [14]. In addition, *SLC6A1* was recently found to be significantly associated with de novo mutations in a multinational schizophrenia cohort study[15].

For gene-based burden tests, the PAGEANT power calculator [16] was used to estimate power in three different scenarios: 1) where minor allele frequency is independent of expected variance explained, 2) where minor allele frequency is independent of genetic effects measured in terms of unit per copy of an allele, and 3) where minor allele frequency is negatively correlated with genetic effect (see: https://andrewhaoyu.shinyapps.io/PAGEANT/).

The required alpha was set to 0.0001 and power was calculated for a range of values for expected variance explained (from 0.05% to 0.5%), with an expected 20% of variants in each gene being causal variants.

This indicated that there would be 0.999 power when the expected variance explained by a gene was above 0.16%, in all three scenarios, and that there would be >0.95 power when the expected variance explained by a gene was above 0.1% in all three scenarios.

Table S2. Power to detect associated genes with a range of variances explained in three scenarios, with current sample size

EV(Percent)	Scenario 1 Mean Power	Scenario 2 Mean Power	Scenario 3 Mean Power
0.05	0.52	0.52	0.52
0.075	0.82	0.82	0.82
0.1	0.95	0.95	0.95
0.125	0.989	0.989	0.989
0.1625	0.999	0.999	0.999
0.275	1	1	1
0.3875	1	1	1
0.5	1	1	1

Enrichment analysis

Enrichment analysis is used in three analyses in this study. First, it is used in hypothesis-free testing, to identify pathways that are associated with the latent psychiatric traits. Secondly, it is used to test whether genes associated with matched Mendelian disorders are enriched for genetic associations for latent psychiatric traits. Thirdly, it is used to test whether genes previously associated with schizophrenia and bipolar disorder through common variant tests (GWAS) will also be enriched for rare genetic variants associated with latent psychiatric traits underlying the psychiatric disorders.

We use competitive gene set analysis in MAGMA [17] to test whether the genes in our gene sets are more associated with these traits than all other genes outside the gene set. The competitive gene set analysis controls for confounding features of genes, such as gene length and linkage disequilibrium.

It should be noted that the authors of MAGMA remark that the number of genes in gene set enrichment analysis is related to the statistical power of these analyses. That is, when there is a larger number of genes in gene-set enrichment analyses, the statistical power to detect associations is higher. However, there is no publicly available or standard method to estimate power for enrichment analyses in MAGMA.

Pre-registration details and deviations

Methods were piloted with the analysis for depression, and pre-registered for the analyses for schizophrenia and bipolar disorder (available through the OSF: https://osf.io/w8jyu/).

We made three deviations from the pre-registration analysis:

1. **Search terms used for OMIM database search for matched Mendelian phenotypes**

In our pre-registered methods, we planned to search for Mendelian traits that presented with schizophrenia using the following search terms within the 'clinical features' subsection of entries on OMIM: schiz* OR **mania** OR **manic**.

However, in the actual analysis, we used the following search terms within the 'clinical features' subsection of entries on OMIM: schiz* OR **psychotic** OR **psychosis**. This change was because of an error in the search terms specified in pre-registration (duplication of the search terms used for bipolar disorder).

Also, we planned to search for Mendelian traits that presented with bipolar disorder using the following search terms within the 'clinical features' subsection of entries on OMIM: mania OR manic OR **depress***.

However, in the actual analysis, we narrowed down the search terms for bipolar disorder by searching for the following terms within the 'clinical features' subsection of entries on OMIM: mania OR manic OR **bipolar**, to retain only entries that had a relevant match to bipolar disorder.

2. **Outlier removal for schizophrenia phenotype**

To remove outliers, we used the multivariate measure Robust Mahalanobis Distance [18,19] to exclude individuals who scored at or above the 99th percentile of distance from the centroid of the data. This outlier removal procedure was not performed for schizophrenia, as the prevalence of affirmative responses was very low for the items f.20474.0.0 (strange force; N=343 responded 'yes' after outlier

removal) and f.20468.0.0 (unjust plot; N=845 responded ‘yes’ after outlier removal), which prevented the calculation of a positive definite covariance matrix for factor analysis.

3. Filtering of OMIM results

In our preregistered analysis, we planned to retain all genes associated with each matched Mendelian disorder and grouped them as a "gene set." Competitive gene-set enrichment analysis would be performed (using the MAGMA software) to test whether they exhibit an enrichment of predicted deleterious variants associated with the latent traits we identified.

However, we noticed several matched Mendelian disorders on OMIM were not precisely linked to specific genes or loci but were instead associated with large segments of chromosomes due to the low specificity of linkage studies (these segments occasionally contained >100 genes). Since this would introduce noise into the gene set, as many genes would be unrelated to the matched disorder, we sought to filter these gene sets by length. We plotted the number of genes contained in each associated OMIM segment for each matched phenotype and decided to filter out segments that contained 5 or more genes, as the majority of segments contained <5 genes for matched phenotypes for all three phenotypes (shown in Figure S25).

4. GCTB estimation of heritability, selection and polygenicity

In our preregistered analysis, we planned to estimate the heritability, selection and polygenicity of each of the general latent traits using the GCTB software[20]. However, we found that the computational time and memory required to estimate these parameters for all the traits across all chromosomes was unfeasible, even using the nested model of GCTB with the openMPI parallelised version of the software. We therefore restricted the analysis to data from chromosome 1. The distribution of sigma (selection coefficient) and pi (polygenicity) are not expected to vary between chromosomes and are not additive, so estimates from a single chromosome are expected to be representative of estimates from across the genome.

Further exploratory analyses

Due to the suggestions from reviewers, we also calculated the Lambda 1000 and LDSC intercept using the LDSC software (reported in Table S13).

Supplementary methods

These methods are also available to view through the pre-registration plan available on the OSF (https://osf.io/w8jyu/).

Phenotype creation

Factor analysis for latent trait estimation

A literature search was conducted in order to select the range of questions in the Mental Health Questionnaire that were used for factor analysis, for each disorder. Further, it was used as a guide to determine the theoretical structure of factors and item loadings, and to determine whether to specify a higher-order general factor model. The details of this procedure were as follows:

Studies were identified by searching PubMed (https://pubmed.ncbi.nlm.nih.gov/) for papers relating to factor analysis for either "schizophrenia" or "bipolar disorder" (or "bipolar I disorder", "bipolar II disorder" or "cyclothymia"). Studies must have a sample size ≥300 and be published after 2004. Studies must have used factor analysis and must have used items directly relating to the mental disorder of interest (i.e. the items must relate to the disorder, rather than the disorder being used for external validation or comparison to another trait). Studies must also have reported a summary of factor loadings for individual items.

In order to select the range of questions in the Mental Health Questionnaire to be used for factor analysis, questions that did not ask about signs / symptoms in any of the diagnostic criteria (DSM-5 or ICD-10) were excluded. Questions must have been clinically relevant to the disorder of interest, rather than another outcome or trait that was compared to the psychopathology.

Questions that directly asked about signs / symptoms present on either or both the DSM and ICD criteria for the disorder were included as items. Furthermore, questions that asked about signs and symptoms that moderately-highly load onto the same factors as the direct questions do (>0.5) in the papers matching the criteria of the literature review were included.

For depression, the factor model matched that from Jermy et al.[21] which used the same items and dataset. 18 questions were included, relating to current symptoms of depression and anxiety: 7 questions from the Generalised Anxiety Disorder Assessment (GAD7), 2 questions relating to subjective well-being and 9 questions from the Patient Health Questionnaire (PHQ9). For bipolar disorder, 15 questions were included, relating to lifetime symptoms of bipolar disorder (7 questions from the CIDI: Depression scale and 8 questions from the CIDI: Mania scale). For schizophrenia, 12 questions were included, relating to symptoms of schizophrenia (4 questions from the PHQ-9, 4 lifetime questions from the CIDI: Mania scale and 4 lifetime questions from the CIDI: Psychosis scale).

Items related to symptoms during a potential episode of mania (in schizophrenia and bipolar disorder) were nested as follow-up items to questions that asked participants whether they had experienced a period of irritability or a period of feeling high, excited or hyper for 2 weeks. We coded negative responses to these questions as negative responses to the nested items.

Missing values

For items where individuals responded with "don't know" or "prefer not to answer", missing values were imputed using responses to answered questions. Since items were coded using ordinal scales, individuals' missing values on MHQ items were imputed by predicting them with an ordinal regression model that uses their responses on the other variables, with the regressionImp() function in the VIM package in R. This was done for items relating to each disorder separately.

Individuals who still had any missing values after this procedure (for example, because they had answered "don't know" or "prefer not to answer" to several questions) were removed from further analysis.

Outlier removal

To detect individuals who were likely outliers, we considered individuals' responses on all disorder-related questions of the Mental Health Questionnaire, in a multivariate analysis. The generalised minimum covariance determinant method (GenMCD) was used to detect outliers in this ordinal dataset. This approach is a variant of the minimum covariance determinant method, which randomly samples the data to find a subsample with the lowest covariance (i.e. a cluster that excludes a potential group of outliers), and uses this as the "centroid" [18,19]. GenMCD specifically uses Chi-squared distance and correspondence analysis to detect outliers in ordinal data (instead of Euclidean distance, which is used in Mahalanobis calculations) [22].

The OuRS and GSVD packages in R was used to conduct GenMCD outlier detection, with the function cat.mcd(). 500 subsamples of the data were randomly generated to find a subsample with the smallest covariance; the size of the subsample was set to alpha=0.5 (the smallest subsample size allowable). Individuals scores on this measure (Robust Mahalanobis Distance) that were at or above the 99th percentile will be considered outliers and removed before further analysis. This was done for items relating to each disorder separately.

Exploratory factor analysis

Half the participants with phenotype data were randomly selected to conduct exploratory factor analysis, and the remaining half were used for confirmatory factor analysis subsequently. Kurtosis and skew were computed for all variables.

To assess the suitability of the data for factor analysis, Bartlett's test of sphericity and the Kaiser-Meyer-Olkin (KMO) test were conducted. Bartlett's test of sphericity assesses whether the correlation matrix of items in the data are significantly different from an identity matrix (the null hypothesis of no underlying factor structure). Since Bartlett's chi-squared statistic is also a function of sample size, the KMO test statistic will also be evaluated. The Kaiser-Meyer-Olkin test assesses the size of partial correlations between items (i.e. correlations between items after accounting for the effects of other correlations), where small partial correlations indicate common variance between items and the factorability of the data by extension; a higher KMO test statistic (Measure of Sampling Adequacy, MSA) reflects higher factorability. These must have shown a significant chi-squared statistic (Bartlett's and KMO statistic >0.6).

In order to select the number of factors to retain, eigenvalues were estimated and plotted as a Scree plot to estimate the number of factors that should be retained in dimensionality reduction. Additionally, parallel analysis was conducted using the fa.parallel() function in the psych package, using the weighted least squares (wls) factoring method. Finally, the Minimum Average Partial (MAP) criterion and very simple structure (vss) criterion were calculated using the oblimin rotation method (an oblique rotation method, which allows for slight correlation between factors) and the minimum residual factoring method. The MAP criterion provides a lower bound of the number of factors that the data should be reduced to, while parallel analysis provides an upper bound.

For factor rotation, we used weighted least squares factoring with oblique geomin rotation, and used Thurstone's rules for complexity to select preferable models:

- Each variable should have at least one 0 loading
- Each factor should have the same number of zeroes as there are factors
- Every pair of factors should have several variables that load onto one factor but not on the other
- Whenever more than four factors are extracted, each pair of factors should have a large proportion (>60%) of variables which do not load on either factor
- A minimum of three indicators per latent factor

- Factor loadings should be dropped if they are <0.3 unless the lit review criteria indicates I should keep them for theoretical reasons
- Factors should not load on multiple items highly (>0.5; this is because complex items may indicate a bad item)
- Check percent of variance explained by full model: ≥50% is required
- Check model fit using Tucker-Lewis Index (TLI) and root mean square error of approximation (RMSEA) indices. Select a model with high TLI (must be >0.90 but >0.95 is preferable) and low RMSEA (must be <0.08 but <0.05 is preferable)
- If model fits or factors are not theoretically coherent after comparing these, adjust the number of factors

In post-hoc analysis, the model solution was adjusted by comparing the Bayesian Information Criterion (BIC) between models with varying number of items that had loadings higher than 0.3 or 0.4.

The factors from the model solution was chosen for retention and extracted using the weighted least squares (wls) factoring method and geominQ rotation (an oblique rotation method), using the fa() function in the psych package.

Confirmatory factor analysis

The model solution from exploratory factor analysis was validated using the remaining half of the dataset. The Comparative Fit Index (CFI) and TLI should be > 0.9 but > 0.95 is preferable, RMSEA and Standardised Root Mean Square (SRMR) should be < 0.08 but < 0.05 is preferable.

General factor solution

If the literature search indicates the inclusion of additional hierarchical general factors, hierarchical factors were retained as phenotypes if CFI and TLI >0.95 and RMSEA and SRMR <0.05. Otherwise, the other estimated latent factors will be used as phenotypes.

Factor scoring

Individuals' factor scores were calculated for each of the selected latent traits for each disorder. That is, individuals' scores were calculated for all the factors in the chosen model (if a hierarchical model was not chosen), or only for the general factors in the chosen model (if a hierarchical model was chosen). Factor scores were calculated using the empirical Bayes method using the lavPredict() function in the lavaan package in R.

Enrichment analysis

We grouped all matched genes (described below) as a gene set and performed competitive gene-set enrichment analysis using MAGMA to test for an enrichment of predicted deleterious variants in these genes.

Matched Mendelian disorders
To identify genes linked to Mendelian disorders exhibiting relevant clinical features, we conducted an advanced search on OMIM: tx_clinical_features:X, where X was depress* (for depression); schiz* OR psychosis OR psychotic (for schizophrenia); and bipolar OR mania OR manic (for bipolar disorder). We manually filtered search results to relevant phenotypes and restricted associated regions to those that contained <5 genes each (Figure S24).

Matched common variant GWAS
We obtained gene-level summary data from the three largest GWAS of matched psychiatric illnesses: Wray et al.[23] for major depression, with UK Biobank data excluded and the analysis restricted to variants with a MAF >1% (58 genes); Mullins et al.[24] of 40 000 cases of bipolar disorder (126 genes); and Ripke et al.[25] of 69 000 cases of schizophrenia (360 genes)

Genotype data

Quality control

Out of the 503,328 individuals in the total UK Biobank sample, genetic data was collected from 488,377 participants. DNA was extracted from blood samples that were collected during recruitment and genotyped in 106 sequential batches across collection centres.

Genotyping was carried out using two similar genotyping arrays: 49,950 were genotyped using the Affymetrix UK BiLeve Axiom Array (807,411 markers) as part of the UK BiLeve study. The remaining 438,427 participants were genotyped using the Applied Biosystems UK Biobank Axiom Array, which has 825,927 markers (95% shared marker content with the UK BiLeve Axiom Array) [26]. Individuals were excluded if they had missing covariate information (relating to the batch, collection centre or plate containing their genotype data).

Out of the 825,927 markers present on the UK Biobank Axiom genotype array, 280,838 markers cover low-frequency variation (European MAF 1-5%). The genotype array also covers rare variants (European MAF <1%), including 30,581 markers covering rare protein truncating variants, and 80,581

covering other rare coding variants [27]. This European panel was defined by Affymetrix as the GBR, CEU, FIN, IBS and TSI samples in the 1000 Genomes Consortium [28].

Genotype data was aligned to Build GRCh37 and will be subjected to several steps of quality control: a maximum missing genotype filter of 0.02 for genetic variants and 0.02 for participants, exclusion of non-Caucasians, of related individuals, of gender/sex mismatches, and a Hardy-Weinberg Equilibrium threshold of $1x10^{-8}$. Participants' relatedness was estimated prior to release by the UK Biobank, using the KING software[29]. We removed participants with a relatedness >0.044 with others using a greedy algorithm (GreedyRelate, i.e. removing the child in a child-parent trio)[30].

Individuals' phenotype scores will be adjusted for the first 20 principal components, which will be estimated using fastPCA with a SNP window size of 1500 SNPs, a SNP window shift of 150 SNPs, and a linkage disequilibrium threshold of $r^2 > 0.02$ for pruning. Additionally, their phenotype scores will be adjusted for their collection centre, genotype batch.

Imputation

The UK Biobank uses a multi-step process for imputation. After performing quality control on genotyped markers, pre-phasing is conducted using the SHAPEIT3 software, and imputes variants with the IMPUTE4 software (for fast imputation with the IMPUTE2 method). Two reference panels are used for imputation [31]. Firstly, the 1000 Genomes phase 3 reference panel [32] and the UK10K reference panels [33] were merged: these panels include SNPs, short indels and larger structural variants and totals 12,570 haplotypes. Secondly, the HRC reference panel has only SNPs but includes more haplotypes (64,976) and is more useful for imputing rare variants [34]. Thus, SNPs are imputed using both panels and HRC-imputed SNPs are retained preferentially when imputations differed [31].

Regarding the quality of rare variant imputation, Pistis et al. [35] analysed the IMPUTE software's INFO metric, which is a measure of confidence with which an imputation is called, by their study sample of the SardiNIA and MCTFR cohorts to three reference panels. They found that study-specific reference panels increase the accuracy of imputation and noted that a standard INFO threshold of >0.4 is efficient at discriminating the accuracy of common variant imputations. They found that this threshold is too lenient to remove low-quality rare variant imputations and recommend using an INFO score threshold of >0.7, for variants with a MAF <1%.

However, genotyped variants also have variable validity, especially at lower allele frequencies. For example, Wright et al. [36] evaluate the validity of rare genotyped variants on UK Biobank arrays by plotting clusters of genotype calls and manual evaluation of the clusters' separation. Using this method,

they estimate the number of false positives and true positives of 2,928 variants at various frequencies. For variants with MAF < 0.0005%, 100% of calls are estimated to be false positives (false positive rate by MAF bin: MAF 0-0.0005% = 100%, MAF 0.0005-0.001% = 90.06%, MAF 0.001-0.005% = 78.87%, MAF 0.005-0.01% = 33.25%). This indicates that genotyped variants with a MAF < 0.005% are more likely to be false positives than true positives.

We therefore used an INFO score threshold of >0.7 for all imputed variants, as recommended by Pistis et al. [35] To deal with genotype variant quality, we used a hard-call threshold of >0.9 (i.e. genotype calls with an uncertainty greater than 0.1 are excluded) and used a conservative MAF threshold in this study for association testing, by excluding SNPs with a MAF < 0.05% (a minor allele count ≈ 67) for GWAS and burden tests. For heritability estimation, we excluded SNPs with a MAF of < 0.005%.

Functional prediction of genetic variants

Variants were annotated through the ANNOVAR annotation software according to gene-level and functional-level annotation, with ensGene for gene-level annotation [37] and dbNSFP33a for functional annotation [38].

The functional impact of variants were predicted using several annotation tools: dbscSNV [39], MutationTaster [40], GERP++ [41], FATHMM [42], and SIFT [43].

Variants were considered predicted to be deleterious if they passed the following criteria in any of the following categories, as per the thresholds recommended by the authors of each prediction tool:

- Protein-truncating predicted deleterious variants:
  - Annotated as a frameshift insertion OR frameshift deletion OR stop-gain OR stop-loss OR frameshift block substitution OR splicing variant in ensGene, OR
  - Classifies as "A" (automatically deleterious) in MutationTaster (this refers to variants that are marked as probable-pathogenic or pathogenic in dbSNP ClinVar)
- Splicing predicted deleterious variants:
  - (Note that dbscSNV restricts predictions to single nucleotide variations (SNVs) within splicing consensus regions, -3 to +8 at the 5' splice site and -12 to +2 at the 3' splite site)
  - Scored above a pathogenic level in either dbscSNV 11's random forest (RF) model OR its ADA model (dbscSNV RF > 0.6 OR dbscSNV ADA > 0.6)
- Deleterious missense predicted deleterious variants:

    - Annotated as a non-synonymous single nucleotide variant OR non-frameshift insertion OR non-frameshift deletion OR a non-frameshift block substitution in ensGene AND
    - Scored above a pathogenic level in 4 prediction scoring methods in dbNSFP33a (SIFT score < 0.05 AND MutationTaster prediction: "D" AND GERP++ score ≥ 4.8 AND FATHMM score ≤ 1.5)
- Deleterious non-coding predicted deleterious variants:
    - Did not have score on MutationTaster AND SIFT, AND
    - Scored above a pathogenic level in GERP and FATHMM (GERP ≥ 4.8 AND FATHMM ≤ 1.5)

Heritability estimation

Bayesian approaches have been developed to estimate SNP heritability while jointly estimating other parameters [44,45]. The GCTB software that uses this approach was developed as an alternative to GCTA [20]. This software has a method called BayesS, which estimates SNP heritability (h^2), the polygenicity of a trait (π), and also estimates the relationship between a SNP_j's allele frequency (p) and SNP effect size (β_j) as a parameter (S). This is conducted using a Bayesian mixture prior that is normally distributed with a mean of zero:

$$\beta_j \sim N(0, [2p_j (1-p_j)]^S \sigma_\beta^2)\ \pi + \Phi(1-\pi)$$

GCTB produces similar point estimates of heritability to GREML and can also explicitly model polygenicity and the relationship between SNP effect size and MAF, while being more computationally efficient with large datasets. These qualities make it more suitable for research that aims to estimate the contribution of rare variants to the heritability of a trait [20].

To deal with the large sample size in this study, we used the nested BayesS model for analysis in GCTB (--bayes N, nested BayesC model), with the openMPI parallelised version of the software. As shown in Zeng et al. (2018), this yields indistinguishable outputs as the non-nested method, with reduced computational time. However, its outputs are interpreted differently, as the heritability and polygenicity is estimated for nested windows (rather than individual variants).

We used a nested window size of 1 Mb, with starting values pi = 0.1, $h^2 = 0.1$ and S = 0 (as recommended by [20]). We specify a chain length of 10,000 and a burn-in of 2000 as Markov chain Monte Carlo (MCMC) options.

Heritability estimation will be conducted using first 20 principal components (calculated from genotype data) as numerical covariates. We will estimate 95% high-density intervals around the point estimates, using the HDIntervals package in R.

Enrichment testing

For each psychiatric trait, we searched the OMIM database for entries that mentioned matched phenotypes, manually filtered entries to retain only those that described the intended meaning of the word (e.g. psychological depression rather than ‘depression’ of a parameter). We remapped associated gene loci to build GRCh37 using the NCBI remap tool.[46] These MIM genes/loci are listed in Table S3–S5 below with their associated phenotypes and phenotype MIM numbers.

This resulted in 102 loci for depression, 54 loci for bipolar disorder, and 124 loci for schizophrenia, when accessed via OMIM on 17 Apr 2019, 7 Aug 2020 and 7 Aug 2020 respectively.

We then matched these loci to gene ranges from UCSC’s table browser tool using the hg19 gene list file from Plink v1.9, which accessed the UCSC table browser in May 2014. We plotted the number of genes contained in each locus as shown in Fig S24 and retained only loci containing <5 genes.

This resulted in 97 loci for depression, 40 loci for bipolar disorder, and 108 loci for schizophrenia.

None of the MIM loci overlapped with genes found in common variant GWAS for schizophrenia, bipolar disorder or depression.

OMIM matched phenotypes for depression

Search criteria: Gene Map Search - 'tx_clinical_features:depression (Entries with: gene map locus; Retrieve: gene map)'

Table S3. Matched MIM phenotypes for depression

MIM Gene/Locus	Phenotype	Phenotype MIM number
PPT1, CLN1	Ceroid lipofuscinosis, neuronal, 1	256730
MMACHC	Methylmalonic aciduria and homocystinuria, cblC type	277400

PRDX1, PRXI, PAGA, NKEFA	Methylmalonic aciduria and homocystinuria, cblC type, digenic	277400
GBA	Gaucher disease, type III	231000
GBA	{Lewy body dementia, susceptibility to}	127750
GBA	{Parkinson disease, late-onset, susceptibility to}	168600
MSTO1, MMYAT	Myopathy, mitochondrial, and ataxia	617675
FMO3, TMAU	Trimethylaminuria	602079
XPR1, SYG1, IBGC6	Basal ganglia calcification, idiopathic, 6	616413
PSEN2, AD4, STM2, CMD1V	Alzheimer disease-4	606889
SPAST, SPG4	Spastic paraplegia 4, autosomal dominant	182601
DCTN1, HMN7B	Perry syndrome	168605
ZEB2, ZFHX1B, SMADIP1, SIP1	Mowat-Wilson syndrome	235730
CASR, HHC1, PCAR1, FIH, EIG8, HYPOC1	Hypocalcemia, autosomal dominant	601198
CASR, HHC1, PCAR1, FIH, EIG8, HYPOC1	Hypocalcemia, autosomal dominant, with Bartter syndrome	601198
HTT, HD, IT15, LOMARS	Huntington disease	143100
WFS1, WFRS, WFS, DFNA6, DFNA14, DFNA38, WFSL, CTRCT41	Wolfram syndrome 1	222300
WFS1, WFRS, WFS, DFNA6, DFNA14, DFNA38, WFSL, CTRCT41	Wolfram-like syndrome, autosomal dominant	614296
SNCA, NACP, PARK1, PARK4	Dementia, Lewy body	127750
SNCA, NACP, PARK1, PARK4	Parkinson disease 1	168601
CISD2, WFS2, ZCD2, ERIS	Wolfram syndrome 2	604928
AMACR, CBAS4, AMACRD	Alpha-methylacyl-CoA racemase deficiency	614307
LMNB1, ADLD	Leukodystrophy, adult-onset, autosomal dominant	169500
CSF1R, FMS, HDLS	Leukoencephalopathy, diffuse hereditary, with spheroids	221820
PDGFRB, PDGFR, IBGC4, IMF1, PENTT, KOGS	Basal ganglia calcification, idiopathic, 4	615007
PDGFRB, PDGFR, IBGC4, IMF1, PENTT, KOGS	Kosaki overgrowth syndrome	616592
SNCB	Dementia, Lewy body	127750
TBC1D7, PIG51, TBC7, MGCPH	Macrocephaly/megalencephaly syndrome, autosomal recessive	248000
TBP, SCA17, HDL4	Spinocerebellar ataxia 17	607136
TBP, SCA17, HDL4	{Parkinson disease, susceptibility to}	168600
SGCE, DYT11	Dystonia-11, myoclonic	159900
SLC20A2, MLVAR, GLVR2, IBGC1	Basal ganglia calcification, idiopathic, 1	213600
C9orf72, FTDALS1, FTDALS, ALSFTD	Frontotemporal dementia and/or amyotrophic lateral sclerosis 1	105550
VPS13A, CHAC	Choreoacanthocytosis	200150
IKBKAP, IKAP	Dysautonomia, familial	223900
DYT1, TOR1A	Dystonia-1, torsion	128100

DYT1, TOR1A	{Dystonia-1, modifier of}	
KCNT1, KIAA1422, EIEE14, ENFL5	Epilepsy, nocturnal frontal lobe, 5	615005
TWNK, C10orf2, TWINKLE, PEOA3, IOSCA, MTDPS7, PRLTS5	Progressive external ophthalmoplegia with mitochondrial DNA deletions, autosomal dominant 3	609286
SLC18A2, VAT2, SVMT, PKDYS2	?Parkinsonism-dystonia, infantile, 2	618049
ELP4, PAX6NEB, AN2	?Aniridia 2	617141
CTSF, CLN13	Ceroid lipofuscinosis, neuronal, 13, Kufs type	615362
GTS	Tourette syndrome	137580
CACNA1C, CACNL1A1, CCHL1A1, TS	Timothy syndrome	601005
TPH2, NTPH, ADHD7	{Attention deficit-hyperactivity disorder, susceptibility to, 7}	613003
TPH2, NTPH, ADHD7	{Unipolar depression, susceptibility to}	608516
ATXN8	Spinocerebellar ataxia 8	608768
ATXN8OS, SCA8, KLHL1AS	Spinocerebellar ataxia 8	608768
ATXN8OS, SCA8, KLHL1AS	{Parkinson disease, susceptibility to}	168600
SLITRK1, KIAA1910, TTM	?Trichotillomania	613229
SLITRK1, KIAA1910, TTM	Tourette syndrome	137580
FGF14, FHF4, SCA27	Spinocerebellar ataxia 27	609307
COL4A1, BSVD1, HANAC, ICH, BSVD, RATOR	Brain small vessel disease with or without ocular anomalies	175780
GCH1, DYT5, HPABH4B	Dystonia, DOPA-responsive, with or without hyperphenylalaninemia	128230
PSEN1, AD3, ACNINV3	Alzheimer disease, type 3	607822
PSEN1, AD3, ACNINV3	Alzheimer disease, type 3, with spastic paraparesis and apraxia	607822
PSEN1, AD3, ACNINV3	Alzheimer disease, type 3, with spastic paraparesis and unusual plaques	607822
PSEN1, AD3, ACNINV3	Dementia, frontotemporal	600274
CBG, SERPINA6	Corticosteroid-binding globulin deficiency	611489
AUTS4	{Autism susceptibility 4}	608636
HDC	{Gilles de la Tourette syndrome, susceptibility to}	137580
USP8, HUMORF8, PITA4	Pituitary adenoma 4, ACTH-secreting, somatic	219090
ENFL2	Epilepsy, nocturnal frontal lobe, type 2	603204
POLG, POLG1, POLGA, PEO, SANDO, SCAE, MTDPS4A, MTDPS4B, MIRAS	Mitochondrial recessive ataxia syndrome (includes SANDO and SCAE)	607459
POLG, POLG1, POLGA, PEO, SANDO, SCAE, MTDPS4A, MTDPS4B, MIRAS	Progressive external ophthalmoplegia, autosomal dominant 1	157640
POLG, POLG1, POLGA, PEO, SANDO, SCAE, MTDPS4A, MTDPS4B, MIRAS	Progressive external ophthalmoplegia, autosomal recessive 1	258450
CLN3, BTS	Ceroid lipofuscinosis, neuronal, 3	204200
ASPG2	{Asperger syndrome susceptibility 2}	608631
TTC19, MC3DN2	Mitochondrial complex III deficiency, nuclear type 2	615157

COASY, NBIA6, PCH12	Neurodegeneration with brain iron accumulation 6	615643
GFAP, ALXDRD	Alexander disease	203450
MAPT, MTBT1, DDPAC, MSTD	Dementia, frontotemporal, with or without parkinsonism	600274
MAPT, MTBT1, DDPAC, MSTD	{Parkinson disease, susceptibility to}	168600
KANSL1, KIAA1267, MSL1V1, KDVS	Koolen-De Vries syndrome	610443
CACNA1G, SCA42, SCA42ND	Spinocerebellar ataxia 42	616795
MAFD1, BPAD, MD1	{Major affective disorder 1}	125480
PPNAD4	Pigmented nodular adrenocortical disease, primary, 4	615830
ABCA7, ABCX, AD9	{Alzheimer disease 9, susceptibility to}	608907
DNMT1, MCMT, HSN1E, ADCADN	Cerebellar ataxia, deafness, and narcolepsy, autosomal dominant	604121
CACNA1A, CACNL1A4, SCA6, EIEE42	Episodic ataxia, type 2	108500
PRKACA	Cushing syndrome, ACTH-independent adrenal, somatic	615830
NOTCH3, CADASIL1, CASIL, IMF2, LMNS	Cerebral arteriopathy with subcortical infarcts and leukoencephalopathy 1	125310
C19orf12, NBIA4, SPG43	Neurodegeneration with brain iron accumulation 4	614298
ATP1A3, DYT12, RDP, AHC2, CAPOS	Alternating hemiplegia of childhood 2	614820
ATP1A3, DYT12, RDP, AHC2, CAPOS	Dystonia-12	128235
PANK2, NBIA1, PKAN, HARP	Neurodegeneration with brain iron accumulation 1	234200
PRNP, PRIP, KURU, CJD	Cerebral amyloid angiopathy, PRNP-related	137440
PRNP, PRIP, KURU, CJD	Gerstmann-Straussler disease	137440
PRNP, PRIP, KURU, CJD	Huntington disease-like 1	603218
PRNP, PRIP, KURU, CJD	Insomnia, fatal familial	600072
PRNP, PRIP, KURU, CJD	Prion disease with protracted course	606688
RTEL1, C20orf41, NHL, KIAA1088, DKCB5, DKCA4, PFBMFT3	Dyskeratosis congenita, autosomal dominant 4	615190
RTEL1, C20orf41, NHL, KIAA1088, DKCB5, DKCA4, PFBMFT3	Dyskeratosis congenita, autosomal recessive 5	615190
DNAJC5, DNAJC5A, CSP, CLN4B	Ceroid lipofuscinosis, neuronal, 4, Parry type	162350
KCTD17	Dystonia 26, myoclonic	616398
PLA2G6, IPLA2, INAD1, NBIA2B, NBIA2A, PARK14	Parkinson disease 14, autosomal recessive	612953
PDGFB, SIS, IBGC5	Basal ganglia calcification, idiopathic, 5	615483
CYP2D6, CPD6, P450DB1	{Codeine sensitivity}	608902
CYP2D6, CPD6, P450DB1	{Debrisoquine sensitivity}	608902
ATXN10, SCA10	Spinocerebellar ataxia 10	603516
SHANK3, PSAP2, PROSAP2, KIAA1650, DEL22q13.3, SCZD15	{Schizophrenia 15}	613950
CLCN4, MRX49, MRX15, MRXSRC	Raynaud-Claes syndrome	300114

IL1RAPL1, IL1R8, MRX21, MRX34	Mental retardation, X-linked 21/34	300143
MAOA, BRNRS	Brunner syndrome	300615
MAOA, BRNRS	{Antisocial behavior}	300615
FMR1, FRAXA, POF1	Fragile X syndrome	300624
MECP2, RTT, PPMX, MRX16, MRX79, AUTSX3, MRXSL, MRXS13	Mental retardation, X-linked syndromic, Lubs type	300260
MECP2, RTT, PPMX, MRX16, MRX79, AUTSX3, MRXSL, MRXS13	Mental retardation, X-linked, syndromic 13	300055

OMIM matched phenotypes for bipolar disorder

Search criteria: Gene Map Search - 'tx_clinical_features:mania OR tx_clinical_features:manic OR tx_clinical_features:bipolar (Search in: Entries with: Genemap; Retrieve: gene map)'

Table S4. Matched MIM phenotypes for bipolar disorder

MIM Gene/Locus	Phenotype	Phenotype MIM number
C9orf72, FTDALS1, FTDALS, ALSFTD	Frontotemporal dementia and/or amyotrophic lateral sclerosis 1	105550
NOTCH3, CADASIL1, CASIL, IMF2, LMNS	Cerebral arteriopathy with subcortical infarcts and leukoencephalopathy 1	125310
MAFD1, BPAD, MD1	{Major affective disorder 1}	125480
DYT1, TOR1A, AMC5	Dystonia-1, torsion	128100
HTT, HD, IT15, LOMARS	Huntington disease	143100
PRKAR1A, TSE1, CNC1, CAR, PPNAD1, ACRDYS1	Carney complex, type 1	160980
GBA	{Parkinson disease, late-onset, susceptibility to}	168600
SCZD12	{Schizophrenia 12}	181500
MTHFR	{Schizophrenia, susceptibility to}	181500
CHI3L1, GP39, YKL40, ASRT7	{Schizophrenia, susceptibility to}	181500
DISC2	Schizophrenia	181500
SYN2	{Schizophrenia, susceptibility to}	181500
DRD3, ETM1, FET1	{Schizophrenia, susceptibility to}	181500
SCZD1	{Schizophrenia}	181500
SCZD3	{Schizophrenia}	181500
SCZD5	{Schizophrenia}	181500
SCZD6	{Schizophrenia}	181500
SCZD11	{Schizophrenia}	181500
SCZD2	{?Schizophrenia}	181500
HTR2A	{Schizophrenia, susceptibility to}	181500
SCZD7	{Schizophrenia}	181500
DAOA, G72	{Schizophrenia}	181500

SCZD8	{Schizophrenia}	181500
COMT	{Schizophrenia, susceptibility to}	181500
RTN4R, NOGOR	{Schizophrenia, susceptibility to}	181500
APOL4	{Schizophrenia}	181500
APOL2	{Schizophrenia}	181500
SLC20A2, MLVAR, GLVR2, IBGC1, IBGC2	Basal ganglia calcification, idiopathic, 1	213600
ASS1, ASS	Citrullinemia	215700
GBA	Gaucher disease, type III	231000
MECP2, RTT, PPMX, MRX16, MRX79, AUTSX3, MRXSL, MRXS13	Mental retardation, X-linked, syndromic 13	300055
ABCD1, ALD, AMN	Adrenoleukodystrophy	300100
ABCD1, ALD, AMN	Adrenomyeloneuropathy, adult	300100
CLCN4, MRX49, MRX15, MRXSRC	Raynaud-Claes syndrome	300114
FMR1, FRAXA, POF1	Fragile X tremor/ataxia syndrome	300623
PRNP, PRIP, KURU, CJD	Huntington disease-like 1	603218
SLC25A13, CTLN2, NICCD	Citrullinemia, adult-onset type II	603471
ALG12, CDG1G	Congenital disorder of glycosylation, type Ig	607143
FKBP5, FKBP51	{Major depressive disorder and accelerated response to antidepressant drug treatment}	608516
TPH2, NTPH, ADHD7	{Unipolar depression, susceptibility to}	608516
MDD1	Major depressive disorder 1	608516
HTR2A	{Major depressive disorder, response to citalopram therapy in}	608516
HTR2A	{Seasonal affective disorder, susceptibility to}	608516
MDD2	Major depressive disorder 2	608516
TWNK, C10orf2, TWINKLE, PEOA3, IOSCA, MTDPS7, PRLTS5	Progressive external ophthalmoplegia with mitochondrial DNA deletions, autosomal dominant 3	609286
DEL3q29, MICRODEL3q29	Chromosome 3q29 microdeletion syndrome	609425
EHMT1, EUHMTASE1, KMT1D, DEL9q34, KLEFS1	Kleefstra syndrome 1	610253
DEL15q13.3, MICRODEL15q13.3	Chromosome 15q13.3 microdeletion syndrome	612001
DUP16p11.2, C16DUPp11.2, AUTS14B	{Autism, susceptibility to, 14B}	614671
DUP16p11.2, C16DUPp11.2, AUTS14B	Chromosome 16p11.2 duplication syndrome	614671
PDGFRB, PDGFR, IBGC4, IMF1, PENTT, KOGS	Basal ganglia calcification, idiopathic, 4	615007
CHD2, EEOC	Epileptic encephalopathy, childhood-onset	615369
DUP22q13, C22DUPq13	Chromosome 22q13 duplication syndrome	615538
RBM12, KIAA0765, SCZD19	{Schizophrenia 19, susceptibility to}	617629

CEP85L, C6orf204, LIS10	Lissencephaly 10	618873
TANC2, KIAA1636, IDDALDS	Intellectual developmental disorder with autistic features and language delay, with or without seizures	618906

OMIM matched phenotypes for schizophrenia

Search criteria: Gene Map Search - 'tx_clinical_features:schiz* OR tx_clinical_features:psychotic OR tx_clinical_features:psychosis (Search in: Entries with: Genemap; Retrieve: gene map)'

Table S5. Matched MIM phenotypes for schizophrenia

OMIM Gene/Locus	Phenotype	Phenotype MIM number
C9orf72, FTDALS1, FTDALS, ALSFTD	Frontotemporal dementia and/or amyotrophic lateral sclerosis 1	105550
ITM2B, BRI, ABRI, FBD, RDGCA	Dementia, familial Danish	117300
ATP2A2, ATP2B, DAR	Darier disease	124200
NOTCH3, CADASIL1, CASIL, IMF2, LMNS	Cerebral arteriopathy with subcortical infarcts and leukoencephalopathy 1	125310
ATP1A3, DYT12, RDP, AHC2, CAPOS	Dystonia-12	128235
PRNP, PRIP, KURU, CJD	Cerebral amyloid angiopathy, PRNP-related	137440
PRNP, PRIP, KURU, CJD	Gerstmann-Straussler disease	137440
CACNA1A, CACNL1A4, SCA6, EIEE42	Migraine, familial hemiplegic, 1	141500
CACNA1A, CACNL1A4, SCA6, EIEE42	Migraine, familial hemiplegic, 1, with progressive cerebellar ataxia	141500
HTT, HD, IT15, LOMARS	Huntington disease	143100
SGCE, DYT11	Dystonia-11, myoclonic	159900
DMPK, DM, DMK	Myotonic dystrophy 1	160900
HMBS, PBGD, UPS	Porphyria, acute intermittent	176000
HMBS, PBGD, UPS	Porphyria, acute intermittent, nonerythroid variant	176000
PPOX	Porphyria variegata	176200
HFE, HLA-H, HFE1, MVCD7, TFQTL2	{Porphyria variegata, susceptibility to}	176200
NDN	Prader-Willi syndrome	176270
SNRPN	Prader-Willi syndrome	176270
SCZD12	{Schizophrenia 12}	181500
MTHFR	{Schizophrenia, susceptibility to}	181500
CHI3L1, GP39, YKL40, ASRT7	{Schizophrenia, susceptibility to}	181500
DISC2	Schizophrenia	181500
SYN2	{Schizophrenia, susceptibility to}	181500

DRD3, ETM1, FET1	{Schizophrenia, susceptibility to}	181500
SCZD1	{Schizophrenia}	181500
SCZD3	{Schizophrenia}	181500
SCZD5	{Schizophrenia}	181500
SCZD6	{Schizophrenia}	181500
SCZD11	{Schizophrenia}	181500
SCZD2	{?Schizophrenia}	181500
HTR2A	{Schizophrenia, susceptibility to}	181500
SCZD7	{Schizophrenia}	181500
DAOA, G72	{Schizophrenia}	181500
SCZD8	{Schizophrenia}	181500
COMT	{Schizophrenia, susceptibility to}	181500
RTN4R, NOGOR	{Schizophrenia, susceptibility to}	181500
APOL4	{Schizophrenia}	181500
APOL2	{Schizophrenia}	181500
TBX1, DGS, CTHM, CAFS, TGA, DORV, VCFS, DGCR	DiGeorge syndrome	188400
TBX1, DGS, CTHM, CAFS, TGA, DORV, VCFS, DGCR	Velocardiofacial syndrome	192430
CLN6, CLN4A	Ceroid lipofuscinosis, neuronal, Kufs type, adult onset	204300
ASS1, ASS	Citrullinemia	215700
TBX1, DGS, CTHM, CAFS, TGA, DORV, VCFS, DGCR	Conotruncal anomaly face syndrome	217095
WFS1, WFRS, WFS, DFNA6, DFNA14, DFNA38, WFSL, CTRCT41	Wolfram syndrome 1	222300
MTHFR	Homocystinuria due to MTHFR deficiency	236250
NAGS	N-acetylglutamate synthase deficiency	237310
DCAF17, C20orf37	Woodhouse-Sakati syndrome	241080
TBC1D7, PIG51, TBC7, MGCPH	Macrocephaly/megalencephaly syndrome, autosomal recessive	248000
ARSA	Metachromatic leukodystrophy	250100
NHLRC1, EPM2A, EPM2B	Epilepsy, progressive myoclonic 2B (Lafora)	254780
EPM2A, MELF, EPM2	Epilepsy, progressive myoclonic 2A (Lafora)	254780
NPC1, NPC	Niemann-Pick disease, type C1	257220
NPC1, NPC	Niemann-Pick disease, type D	257220
ZBTB20, ZNF288, DPZF, PRIMS	Primrose syndrome	259050
GSS, GSHS	Glutathione synthetase deficiency	266130
HEXB	Sandhoff disease, infantile, juvenile, and adult forms	268800
TWNK, C10orf2, TWINKLE, PEOA3, IOSCA, MTDPS7, PRLTS5	Mitochondrial DNA depletion syndrome 7 (hepatocerebral type)	271245

ALDH5A1, SSADH	Succinic semialdehyde dehydrogenase deficiency	271980
HEXA, TSD	[Hex A pseudodeficiency]	272800
HEXA, TSD	GM2-gangliosidosis, several forms	272800
HEXA, TSD	Tay-Sachs disease	272800
SPG20	Troyer syndrome	275900
MYO7A, USH1B, DFNB2, DFNA11	Usher syndrome, type 1B	276900
MMACHC	Methylmalonic aciduria and homocystinuria, cblC type	277400
PRDX1, PRXI, PAGA, NKEFA	Methylmalonic aciduria and homocystinuria, cblC type, digenic	277400
MECP2, RTT, PPMX, MRX16, MRX79, AUTSX3, MRXSL, MRXS13	Mental retardation, X-linked, syndromic 13	300055
PCDH19, KIAA1313, EFMR, EIEE9	Epileptic encephalopathy, early infantile, 9	300088
ABCD1, ALD, AMN	Adrenoleukodystrophy	300100
ABCD1, ALD, AMN	Adrenomyeloneuropathy, adult	300100
NR0B1, DAX1, AHC, AHX, SRXY2	Adrenal hypoplasia, congenital	300200
MECP2, RTT, PPMX, MRX16, MRX79, AUTSX3, MRXSL, MRXS13	Mental retardation, X-linked syndromic, Lubs type	300260
PAK3, MRX30, MRX47	Mental retardation, X-linked 30/47	300558
MAOA, BRNRS	{Antisocial behavior}	300615
MAOA, BRNRS	Brunner syndrome	300615
ZDHHC9, DHHC9, MRXSZ	Mental retardation, X-linked syndromic, Raymond type	300799
RPL10, DXS648, QM, AUTSX5, MRXS35	{Autism, susceptibility to, X-linked 5}	300847
DUPXq25	Xq25 duplication syndrome	300979
RPS6KA3, RSK2, MRX19	Coffin-Lowry syndrome	303600
MED12, TNRC11, TRAP230, HOPA, KIAA0192, OKS, FGS1, OHDOX	Lujan-Fryns syndrome	309520
AFF2, FMR2, FRAXE, MRX2	Mental retardation, X-linked, FRAXE type	309548
PRNP, PRIP, KURU, CJD	Insomnia, fatal familial	600072
PSEN1, AD3, ACNINV3	Dementia, frontotemporal	600274
MAPT, MTBT1, DDPAC, MSTD	Dementia, frontotemporal, with or without parkinsonism	600274
CLN6, CLN4A	Ceroid lipofuscinosis, neuronal, 6	601780
ENFL2	Epilepsy, nocturnal frontal lobe, type 2	603204
DEPDC5, KIAA0645, FFEVF1	Epilepsy, familial focal, with variable foci 1	604364
SCZD10	{Schizophrenia 10}	605419

PINK1, PARK6	Parkinson disease 6, early onset	605909
FTL, NBIA3, LFTD	Neurodegeneration with brain iron accumulation 3	606159
DJ1, PARK7	Parkinson disease 7, autosomal recessive early-onset	606324
ATP13A2, PARK9, KRPPD, SPG78	Kufor-Rakeb syndrome	606693
IDUA, IDA	Mucopolysaccharidosis Ih/s	607015
PSEN1, AD3, ACNINV3	Alzheimer disease, type 3	607822
PSEN1, AD3, ACNINV3	Alzheimer disease, type 3, with spastic paraparesis and apraxia	607822
PSEN1, AD3, ACNINV3	Alzheimer disease, type 3, with spastic paraparesis and unusual plaques	607822
APOE, AD2, LPG, LDLCQ5	{?Alzheimer disease, protection against, due to APOE3-Christchurch}	607822
HTR2A	{Major depressive disorder, response to citalopram therapy in}	608516
HTR2A	{Seasonal affective disorder, susceptibility to}	608516
CYP2D6, CPD6, P450DB1	{Codeine sensitivity}	608902
CYP2D6, CPD6, P450DB1	{Debrisoquine sensitivity}	608902
DEL3q29, MICRODEL3q29	Chromosome 3q29 microdeletion syndrome	609425
CFH, HF1, HUS, ARMD4, AHUS1	Complement factor H deficiency	609814
DFNB47	Deafness, neurosensory, autosomal recessive 47	609946
EHMT1, EUHMTASE1, KMT1D, DEL9q34, KLEFS1	Kleefstra syndrome 1	610253
DEL15q13.3, MICRODEL15q13.3	Chromosome 15q13.3 microdeletion syndrome	612001
DEL1q21, C1DELq21	Chromosome 1q21.1 deletion syndrome	612474
DUP1q21, C1DUPq21	Chromosome 1q21.1 duplication syndrome	612475
FIG4, KIAA0274, SAC3, ALS11, YVS, BTOP	?Polymicrogyria, bilateral temporooccipital	612691
SOBP, JXC1, MRAMS	Mental retardation, anterior maxillary protrusion, and strabismus	613671
SHANK3, PSAP2, PROSAP2, KIAA1650, DEL22q13.3, SCZD15	{Schizophrenia 15}	613950
VPS35, MEM3, PARK17	{Parkinson disease 17}	614203
WFS1, WFRS, WFS, DFNA6, DFNA14, DFNA38, WFSL, CTRCT41	Wolfram-like syndrome, autosomal dominant	614296
HARS1, HARS, USH3B, CMT2W	Usher syndrome type 3B	614504
BCKDK, BDK, BCKDKD	Branched-chain ketoacid dehydrogenase kinase deficiency	614923

KCNT1, KIAA1422, EIEE14, ENFL5	Epilepsy, nocturnal frontal lobe, 5	615005
CHD8, DUPLIN, KIAA1564, AUTS18	{Autism, susceptibility to, 18}	615032
TTC19, MC3DN2	Mitochondrial complex III deficiency, nuclear type 2	615157
SLC1A1, EAAC1, SCZD18, DCBXA	{?Schizophrenia susceptibility 18}	615232
PDGFB, SIS, IBGC5	Basal ganglia calcification, idiopathic, 5	615483
DNAJC6, DJC6, KIAA0473, PARK19	Parkinson disease 19a, juvenile-onset	615528
DNAJC6, DJC6, KIAA0473, PARK19	Parkinson disease 19b, early-onset	615528
CLCN2, EGMA, ECA2, EGI11, EJM8, LKPAT, HALD2	Leukoencephalopathy with ataxia	615651
PDGFRB, PDGFR, IBGC4, IMF1, PENTT, KOGS	Kosaki overgrowth syndrome	616592
DNAJC12, JDP1, HPANBH4	Hyperphenylalaninemia, mild, non-BH4-deficient	617384
RBM12, KIAA0765, SCZD19	{Schizophrenia 19, susceptibility to}	617629
MSTO1, MMYAT	Myopathy, mitochondrial, and ataxia	617675
MCM3AP, MAP80, GANP, PNRIID	Peripheral neuropathy, autosomal recessive, with or without impaired intellectual development	618124
MYORG, NET37, KIAA1161, IBGC7	Basal ganglia calcification, idiopathic, 7, autosomal recessive	618317

Supplementary results and figures

Raw responses to items

Depression phenotype

Participants' answers on 18 depression-related questions in the UK Biobank (7 questions from the Generalised Anxiety Disorder Assessment (GAD7), 2 questions relating to subjective well-being and 9 questions from the Patient Health Questionnaire (PHQ9; see supplementary materials) were used to create a general internalising score through hierarchical factor analysis. This was used to create a continuous phenotype for genetic association testing.

Fig S1. Responses to depression-related questions in the Mental Health Questionnaire of the UK Biobank

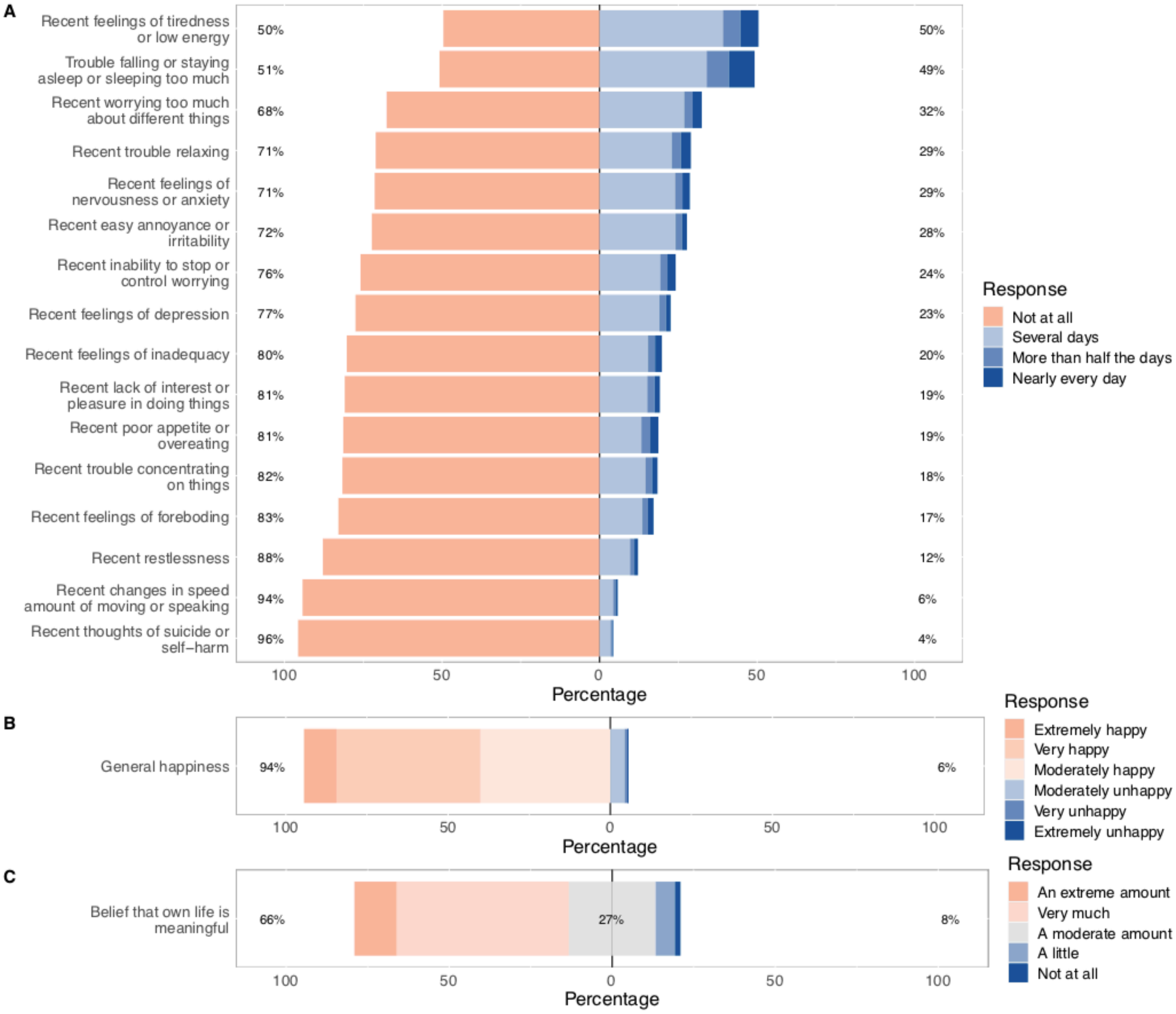

Raw responses to questions in the Mental Health Questionnaire are shown ("don't know" and "prefer not to answer" responses are not shown). 16 of these questions had the same coding for responses (504 coding in UK Biobank), and are displayed together as panel A. Two questions had different coding, and are displayed as panel B and C. Colours, percentages and alignment are used only for illustrative purposes in this figure; they represent the presence of depressive symptoms (orange for absence of depressive symptoms; blue for presence of depressive symptoms). Questions in panel A are in descending order of the proportion of responses that indicate the presence of depressive symptoms. Percentages labelled on the left and right side of the chart represent the total proportion of participants who responded with an orange (e.g. "not at all") or blue response respectively.

Fig S2. Non-responses to depression-related questions in the Mental Health Questionnaire of the UK Biobank

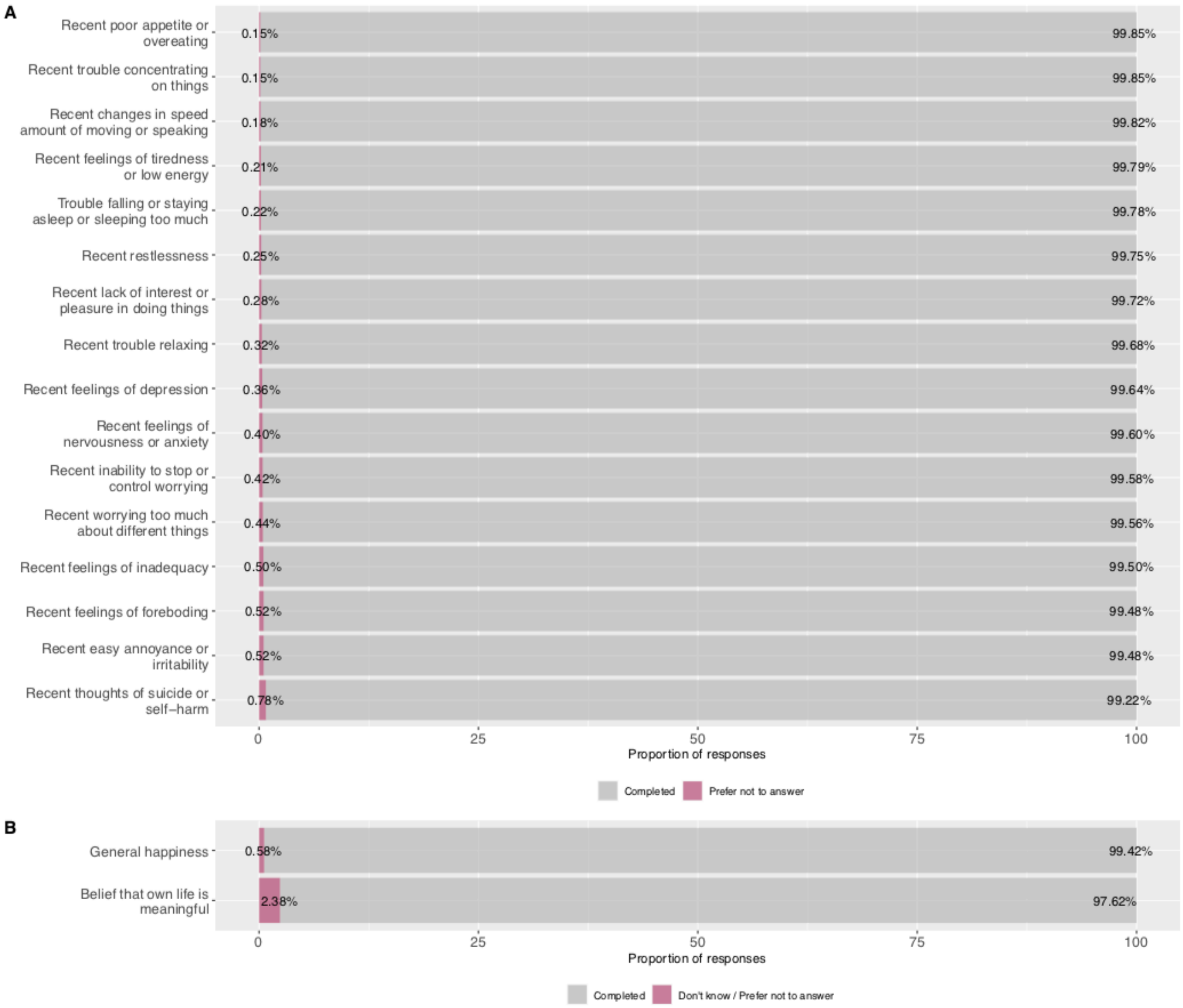

Raw NA responses to questions in the Mental Health Questionnaire ("don't know" and "prefer not to answer") are shown. 16 of these questions had the same coding for responses (504 coding in UK Biobank), and are displayed together as panel A; these 16 questions have an option for "prefer not to answer". Two questions had different coding, and are displayed as panel B and C; these 2 questions have an option for "don't know" and an option for "prefer not to answer."

Figure S2 and S3 display the responses to depression-related questions in the Mental Health Questionnaire. These two figures visualise the responses to questions about frequency of depressive symptoms in the last two weeks, and questions about general happiness and whether participants feel their life is meaningful. Figure S3 displays the proportion of responses that were considered missing values for each question ("don't know" or "prefer not to answer"), while figure 1 displays responses to questions that were not considered missing values.

Across all questions about depressive symptoms, the majority of participants responded that they had experienced the depressive symptoms "not at all" in the last two weeks. Participants responded that they experienced depressive symptoms with varying frequencies. The question asking whether

participants experienced “recent feelings of tiredness or low energy” had the highest proportion of participants who responded in the affirmative (50%), while the question asking whether participants experienced “recent thoughts of suicide or self-harm” had the lowest proportion of participants who responded in the affirmative (4%).

NA responses were most common (2.3%) for the question asking participants whether they believed their own life was meaningful, and least common (0.15%) for the question asking participants whether they had experienced recent poor appetite or overeating.

Schizophrenia phenotype

Participants’ answers on 12 schizophrenia-related questions in the UK Biobank (4 questions from the PHQ-9, 4 questions from the CIDI: Mania scale and 4 questions from the CIDI: Psychosis scale) were used to create a general schizophrenia factor score through hierarchical factor analysis. This was used to create a continuous phenotype for genetic association testing.

Fig S3. Responses to schizophrenia-related questions in the Mental Health Questionnaire of the UK Biobank

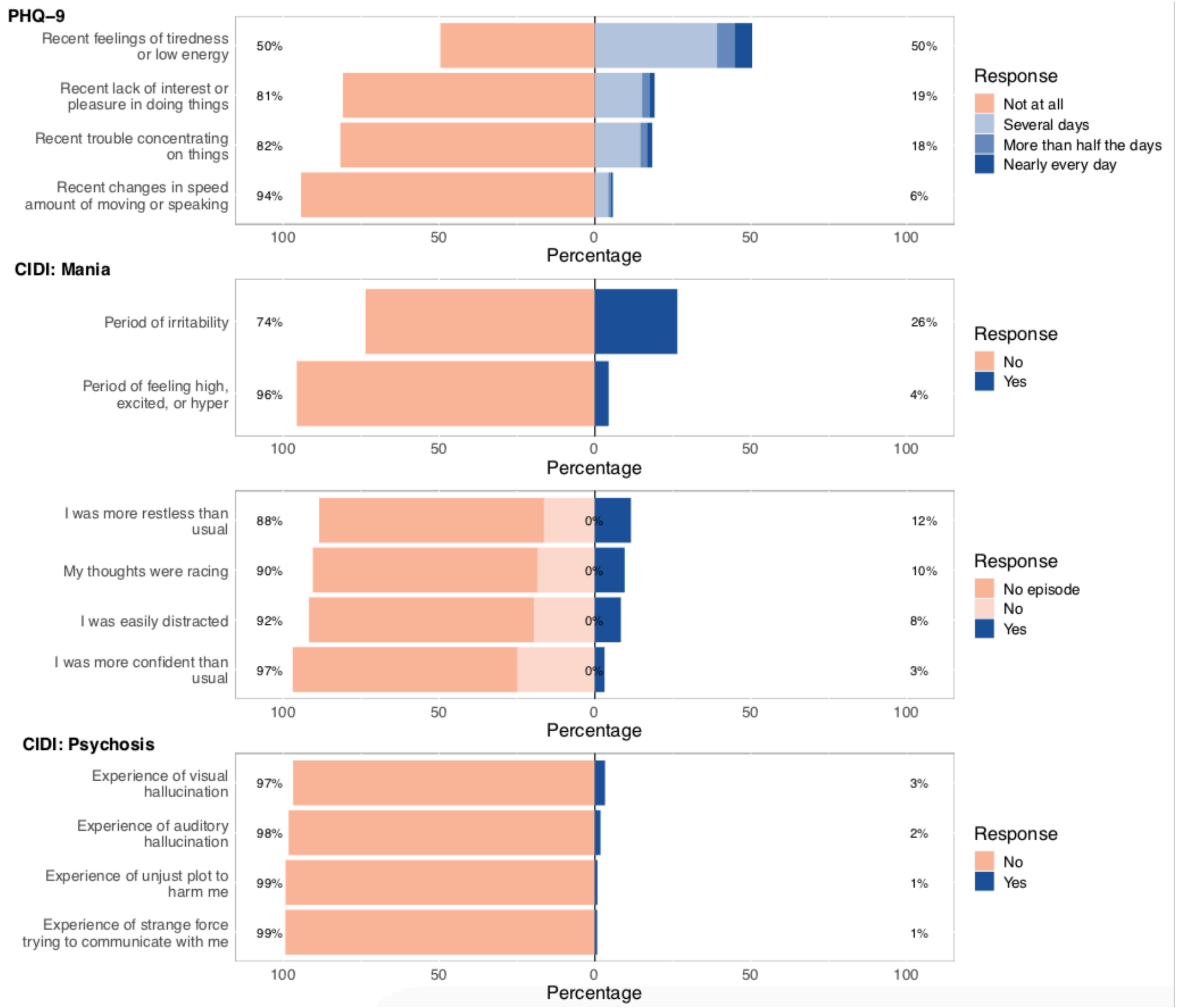

Raw responses to questions in the Mental Health Questionnaire are shown ("don't know" and "prefer not to answer" responses are not shown). Negative answers to either of the leading CIDI: Mania questions (experience of a period of irritability or a period of feeling high, excited or hyper) result in a coding of "no episode" for the following questions, which ask about symptoms experienced during that period. The no episode coding is treated equivalently to a no response in further analysis. Colours, percentages and alignment are used only for illustrative purposes in this figure; they represent the presence of symptoms (orange for absence of symptoms; blue for presence of symptoms). Questions in panel A are in descending order of the proportion of responses that indicate the presence of symptoms. Percentages labelled on the left and right side of the chart represent the total proportion of participants who responded with an orange (e.g. "not at all") or blue response respectively.

Fig S4. Non-responses to schizophrenia-related questions in the Mental Health Questionnaire of the UK Biobank

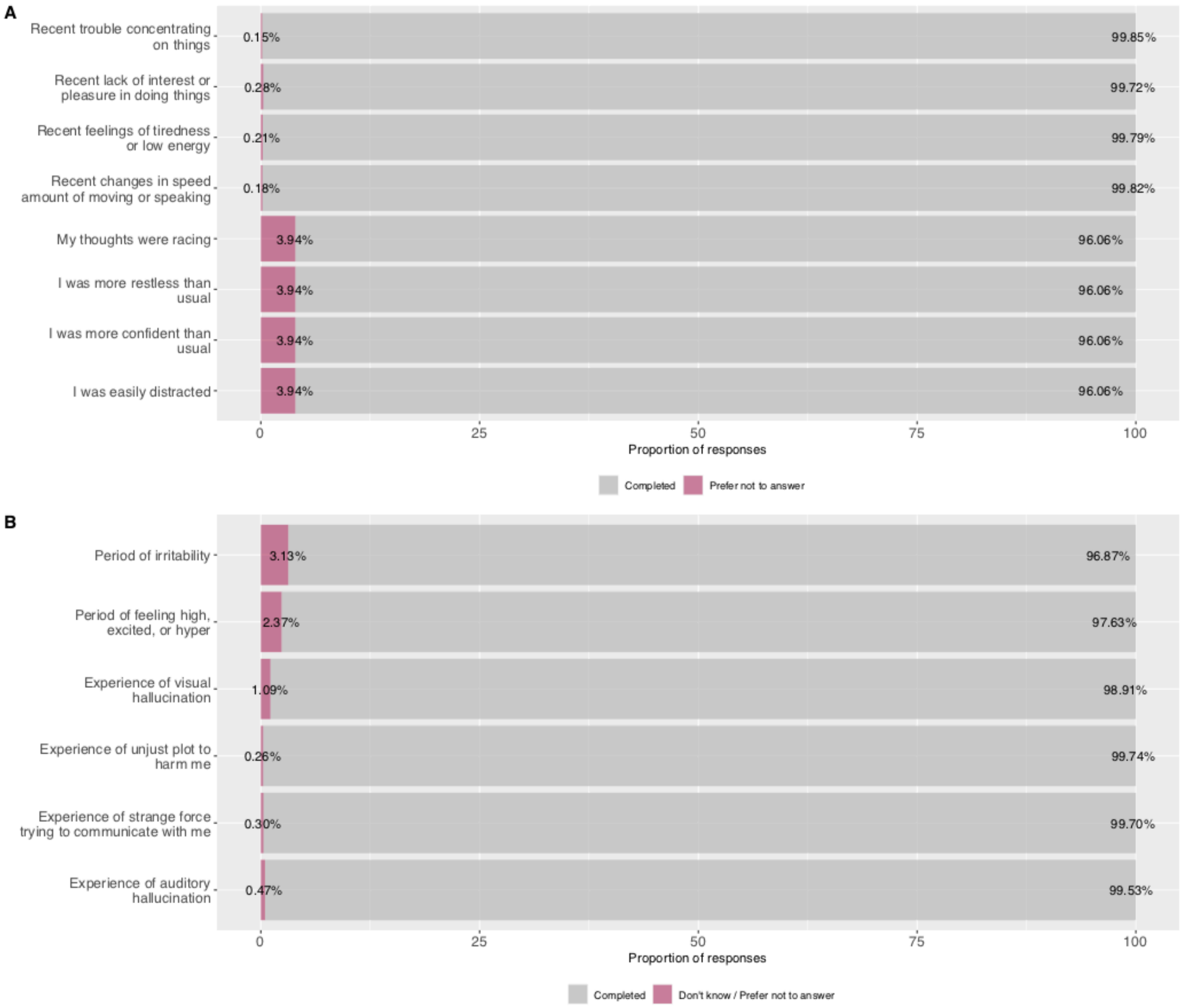

Raw NA responses to questions in the Mental Health Questionnaire ("don't know" and "prefer not to answer") are shown. 8 of these questions had the same coding for responses (504 coding in UK Biobank), and are displayed together as panel A; these 16 questions have an option for "prefer not to answer". 6 questions had different coding, and are described in panel B; these 6 questions have an option for "don't know" and an option for "prefer not to answer."

Figure S4 and S5 display the responses to schizophrenia-related questions in the Mental Health Questionnaire. These two figures visualise the responses to questions about frequency of schizophrenia symptoms in the last two weeks; symptoms experienced during a period of irritability or a period of feeling high, excited or hyper; and symptoms related to hallucinations and delusions that were ever experienced. Figure S5 displays the proportion of responses that were considered missing values for each question ("don't know" or "prefer not to answer"), while figure S4 displays responses to questions that were not considered missing values.

Across all questions about schizophrenia symptoms, the majority of participants responded that they had experienced the schizophrenia symptoms "not at all" in the last two weeks, or that they had not experienced symptoms of hallucinations or delusions, or that they had not experienced a period of irritability or of feeling high, excited or hyper.

Participants responded that they experienced schizophrenia symptoms with varying frequencies. The question asking whether participants experienced “recent feelings of tiredness or low energy” had the highest proportion of participants who responded in the affirmative (50%), while the question asking whether participants experienced “a strange force trying to communicate with me” had the lowest proportion of participants who responded in the affirmative (1%).

NA responses were most common (3.54%) for questions nested in the period of irritability or of feeling high, excited or hyper (i.e. experiencing racing thoughts, feeling more restless than usual, feeling more confident than usual, feeling easily distracted), and least common (0.15%) for the question asking participants whether they had experienced recent trouble concentrating on things.

Bipolar disorder phenotype

Participants’ answers on 15 bipolar disorder-related questions in the UK Biobank (7 questions from the CIDI: Depression scale and 8 questions from the CIDI: Mania scale) were used to create a general bipolar disorder factor score through hierarchical factor analysis. This was used to create a continuous phenotype for genetic association testing.

Fig S5. Responses to bipolar disorder-related questions in the Mental Health Questionnaire of the UK Biobank

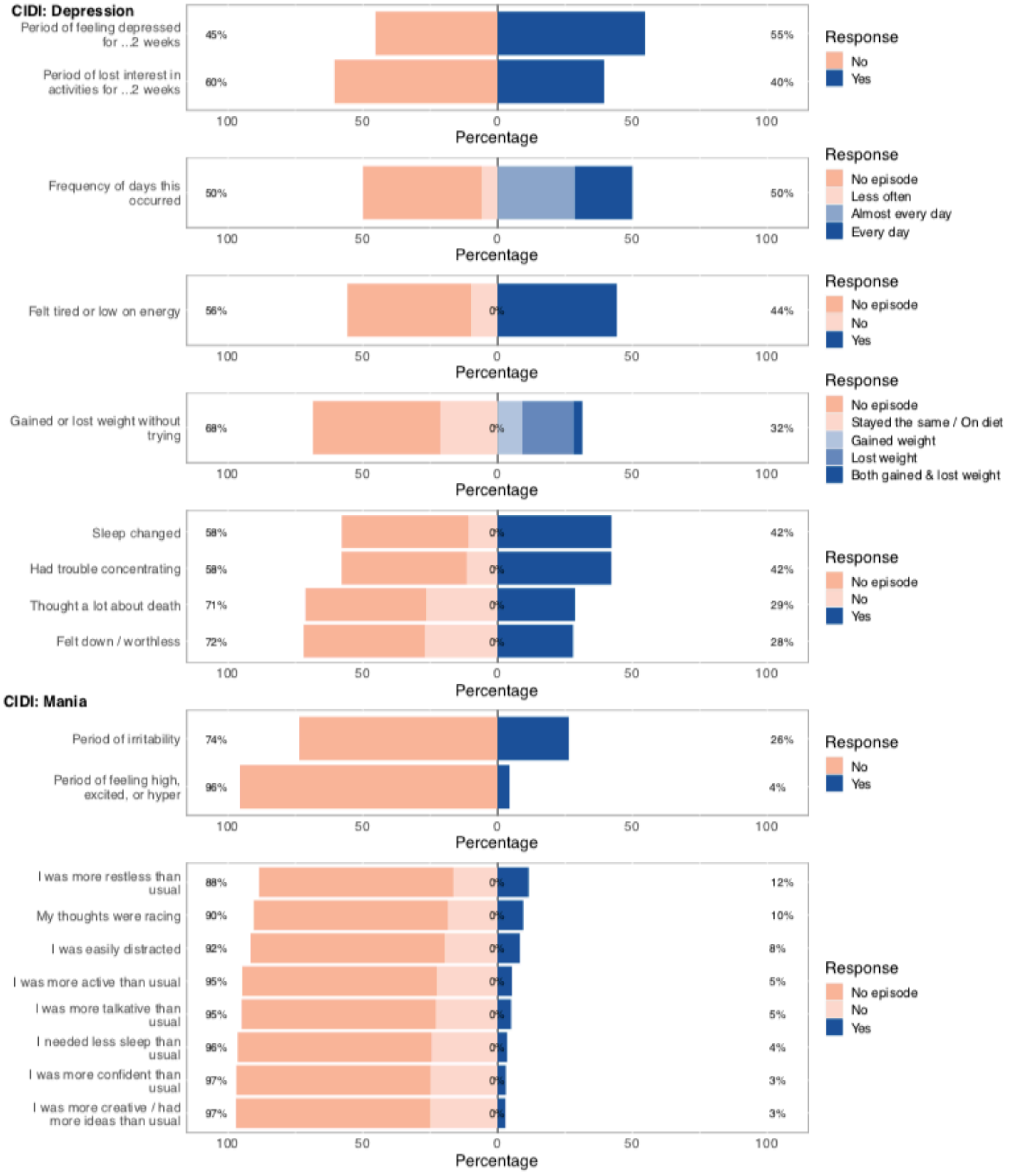

Raw responses to questions in the Mental Health Questionnaire are shown ("don't know" and "prefer not to answer" responses are not shown). Negative answers to either of the leading CIDI: Mania questions (experience of a period of irritability or a period of feeling high, excited or hyper) result in a coding of "no episode" for the following questions. The no episode coding is treated equivalently to a no response in further analysis. The same coding was applied to negative answers to either of the leading CIDI: Depression questions (period of feeling depressed for 2 weeks or period of lost interest for 2 weeks) which ask about symptoms experienced during that period. Colours, percentages and alignment are used only for illustrative purposes in this figure; they represent the presence of symptoms (orange for absence of symptoms; blue for presence of symptoms). Percentages labelled on the left and right side of the chart represent the total proportion of participants who responded with an orange (e.g. "not at all") or blue response respectively.

Fig S6. Non-responses to bipolar disorder-related questions in the Mental Health Questionnaire of the UK Biobank

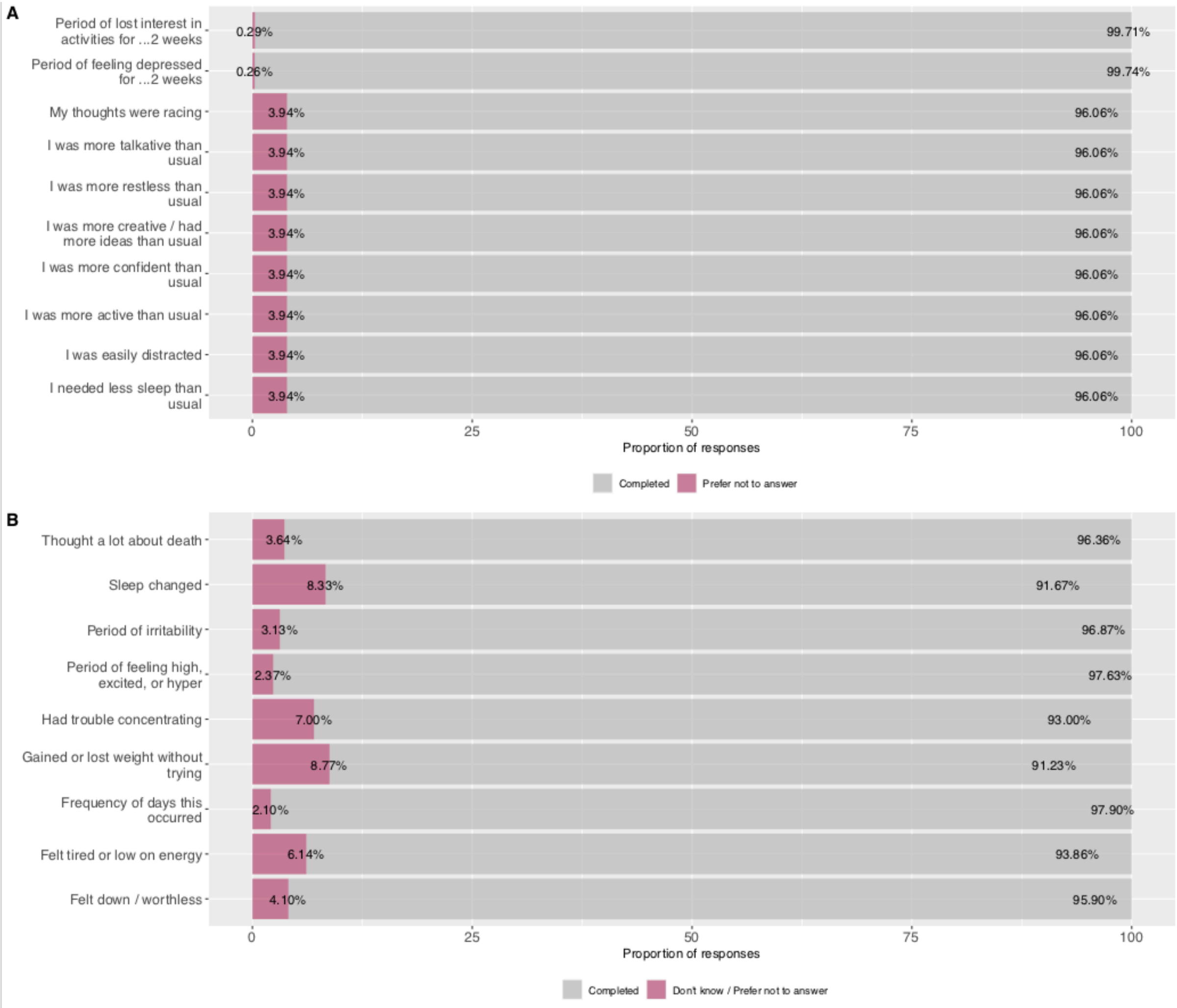

Raw NA responses to questions in the Mental Health Questionnaire ("don't know" and "prefer not to answer") are shown. 10 of these questions had the same coding for responses (504 coding in UK Biobank), and are displayed together as panel A; these 10 questions have an option for "prefer not to answer". 9 questions had different coding, and are described in panel B; these 9 questions have an option for "don't know" and an option for "prefer not to answer."

Figure S6 and S7 display the responses to bipolar disorder-related questions in the Mental Health Questionnaire. These two figures visualise the responses to questions about symptoms experienced during a period of irritability or a period of feeling high, excited or hyper; and symptoms experienced during a period of lost interest or feeling depressed for 2 weeks. Figure 7 displays the proportion of responses that were considered missing values for each question ("don't know" or "prefer not to answer"), while figure 6 displays responses that were not considered missing values.

A majority of participants (55%) responded that they had experienced a period of feeling depressed for 2 weeks, though fewer (40%) responded that they had experienced a period of lost interest for 2

weeks. The majority of participants responded that they had not experienced a period of irritability or a period of feeling high, excited or hyper.

Participants responded that they experienced bipolar disorder symptoms with varying frequencies. The question asking whether participants experienced “felt tired or low on energy” had the highest proportion of participants who responded in the affirmative (44%), while the question asking whether participants agreed with the statement “I was more creative / had more ideas than usual” (during a period of irritability or a period of feeling high, excited or hyper) had the lowest proportion of participants who responded in the affirmative (3%).

NA responses were most common for questions nested in the period of feeling depressed or a loss of interest for 2 weeks (8.77% for experiencing a gain or loss in weight without trying during this period), and least common (0.15%) for the question asking whether they had experienced a period of feeling depressed for 2 weeks (0.26%).

Missing value imputation

Depression phenotype

Out of the total sample in the Mental Health Questionnaire (157,358 individuals), 8401 individuals responded “don’t know” or “prefer not to answer” to one or more depression-related questions. For items where individuals responded with "don't know" or "prefer not to answer", an individual’s missing values were imputed using responses to other questions they had answered, using the function regressionImp() in the VIM package in R.

Individuals who still had any missing values after this procedure (for example, because they had answered "don't know" or "prefer not to answer" to several questions and their values for a particular question could not be imputed) were removed from further analysis. This resulted in N=155,246 individuals with phenotype data, 6,289 of whom had their missing responses imputed.

Figure S8 displays the number of missing responses to depression-related questions after missing values were imputed using this procedure.

Fig S7. Non-responses to depression-related questions in the Mental Health Questionnaire of the UK Biobank after imputation of missing values

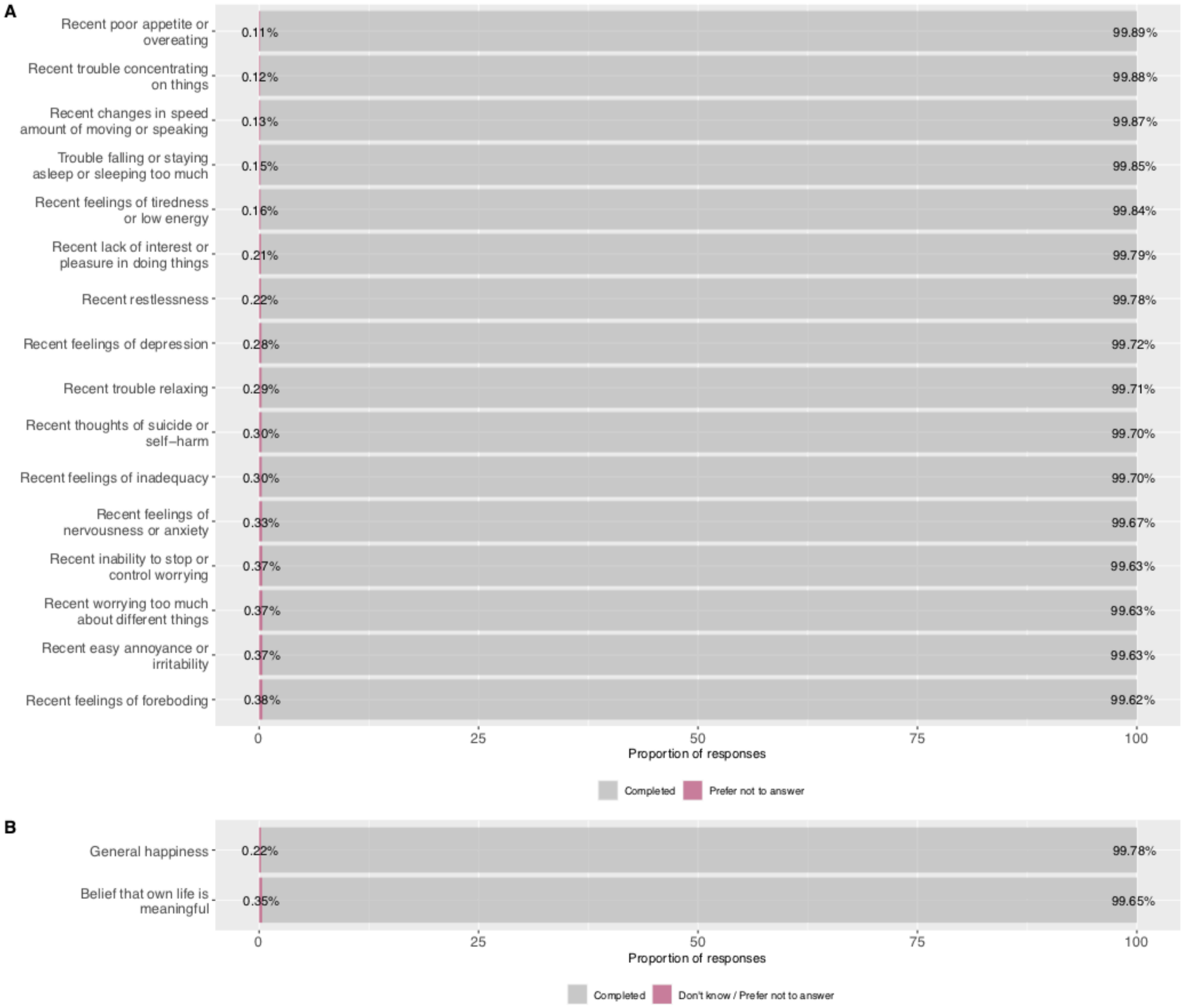

NA responses to questions in the Mental Health Questionnaire ("don't know" and "prefer not to answer") are shown after missing values were imputed. 16 of these questions had the same coding for responses (504 coding in UK Biobank), and are displayed together as panel A; these 16 questions have an option for "prefer not to answer". Two questions had different coding, and are displayed as panel B and C; these 2 questions have an option for "don't know" and an option for "prefer not to answer."

Schizophrenia phenotype

Out of the total sample in the Mental Health Questionnaire (157,366 individuals), 10,938 individuals responded "don't know" or "prefer not to answer" to one or more schizophrenia-related questions. For items where individuals responded with "don't know" or "prefer not to answer", an individual's missing values were imputed using responses to other questions they had answered, using the function regressionImp() in the VIM package in R.

Individuals who still had any missing values after this procedure (for example, because they had answered "don't know" or "prefer not to answer" to several questions and their values for a particular question could not be imputed) were removed from further analysis. This resulted in N=148,681 individuals with phenotype data, 2,253 of whom had their missing responses imputed.

Figure S9 displays the number of missing responses to schizophrenia-related questions after missing values were imputed using this procedure.

Fig S8. Non-responses to schizophrenia-related questions in the Mental Health Questionnaire of the UK Biobank after imputation of missing values

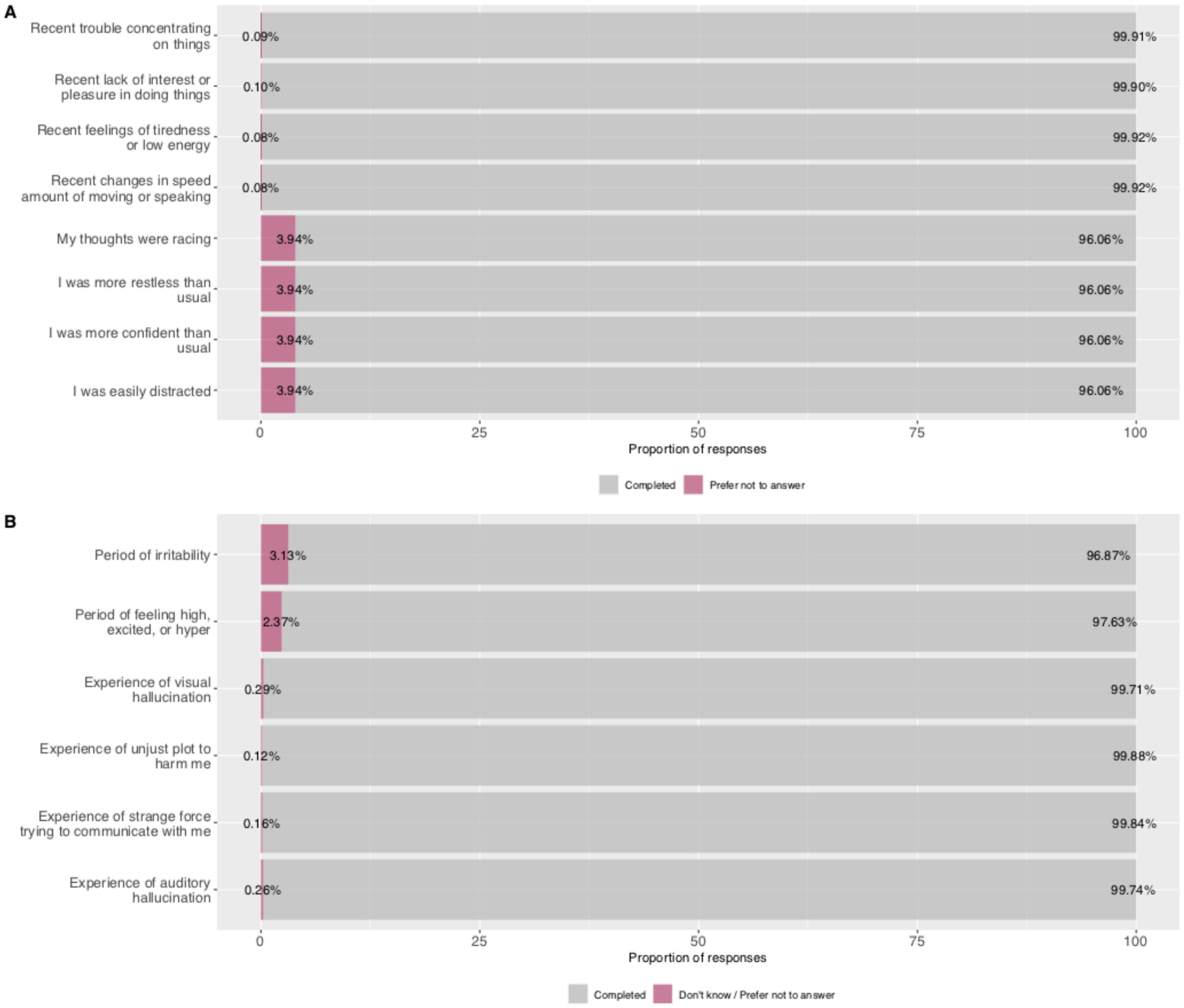

NA responses to questions in the Mental Health Questionnaire ("don't know" and "prefer not to answer") are shown after missing values were imputed. 8 of these questions had the same coding for responses (504 coding in UK Biobank), and are displayed together as panel A; these 16 questions have an option for "prefer not to answer". 6 questions had different coding, and are described in panel B; these 6 questions have an option for "don't know" and an option for "prefer not to answer."

Bipolar disorder phenotype

Out of the total sample in the Mental Health Questionnaire (157,366 individuals), 41,493 individuals responded “don’t know” or “prefer not to answer” to one or more bipolar disorder-related questions. For items where individuals responded with "don't know" or "prefer not to answer", an individual’s missing values were imputed using responses to other questions they had answered, using the function regressionImp() in the VIM package in R.

Individuals who still had any missing values after this procedure (for example, because they had answered "don't know" or "prefer not to answer" to several questions and their values for a particular question could not be imputed) were removed from further analysis. This resulted in N=135,606 individuals with phenotype data, 19,733 of whom had their missing responses imputed.

Figure S10 displays the number of missing responses to bipolar disorder-related questions after missing values were imputed using this procedure.

Fig S9. Non-responses to bipolar disorder-related questions in the Mental Health Questionnaire of the UK Biobank after imputation of missing values

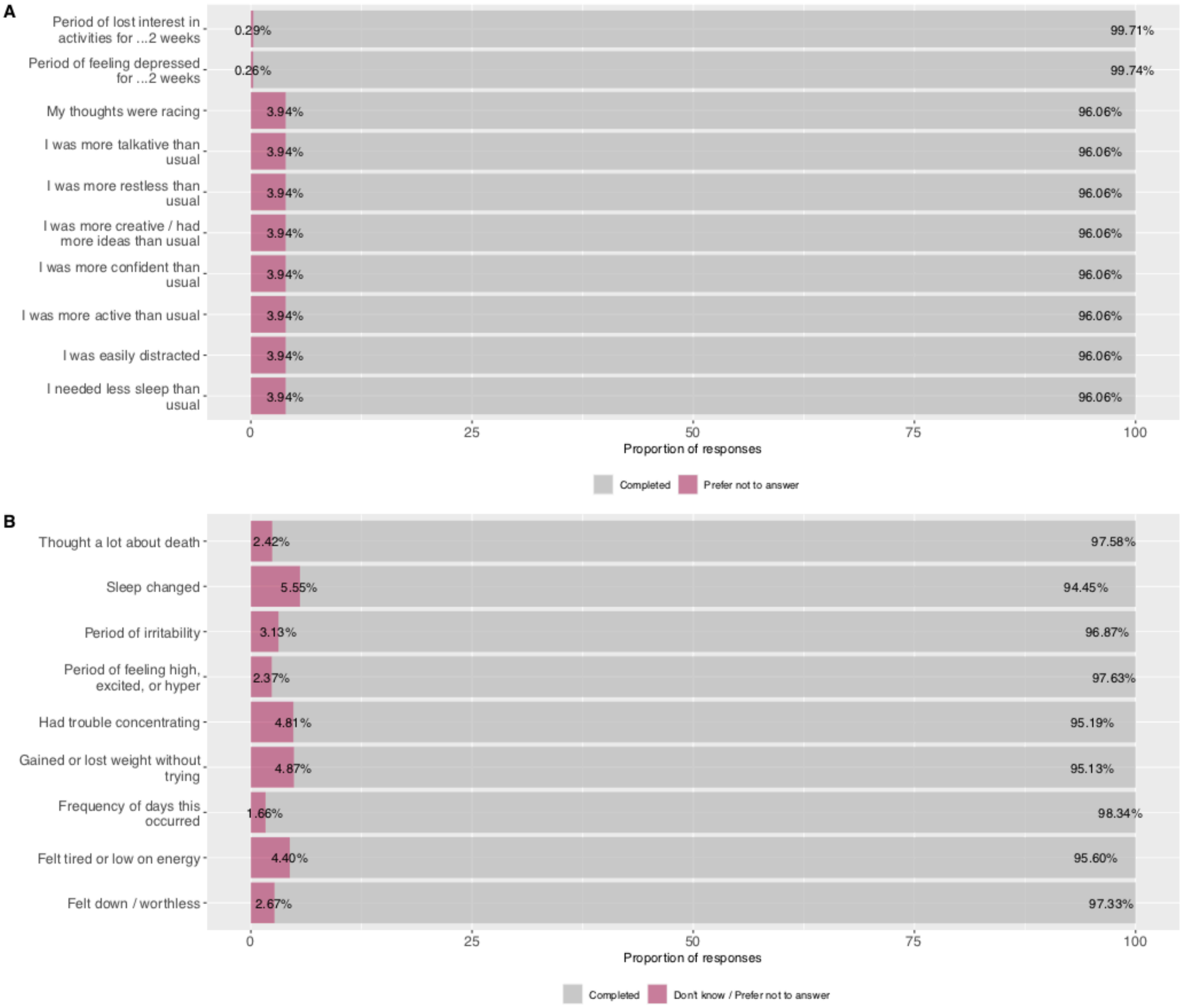

NA responses to questions in the Mental Health Questionnaire ("don't know" and "prefer not to answer") are shown after missing values were imputed. 8 of these questions had the same coding for responses (504 coding in UK Biobank), and are displayed together as panel A; these 16 questions have an option for "prefer not to answer". 6 questions had different coding, and are described in panel B; these 6 questions have an option for "don't know" and an option for "prefer not to answer."

Outlier removal

Depression phenotype

Individuals' scores were calculated for a multivariate outlier detection measure, Robust Mahalanobis Distance [22]. Those who scored at or above the 99th percentile on this measure were considered outliers and were removed before further analysis. In the depression data, 1,553 individuals exceeded this threshold, which left N=153,693 individuals with phenotype data.

Fig S10. Responses to depression-related questions in the Mental Health Questionnaire of the UK Biobank after imputation of missing values and removal of outliers

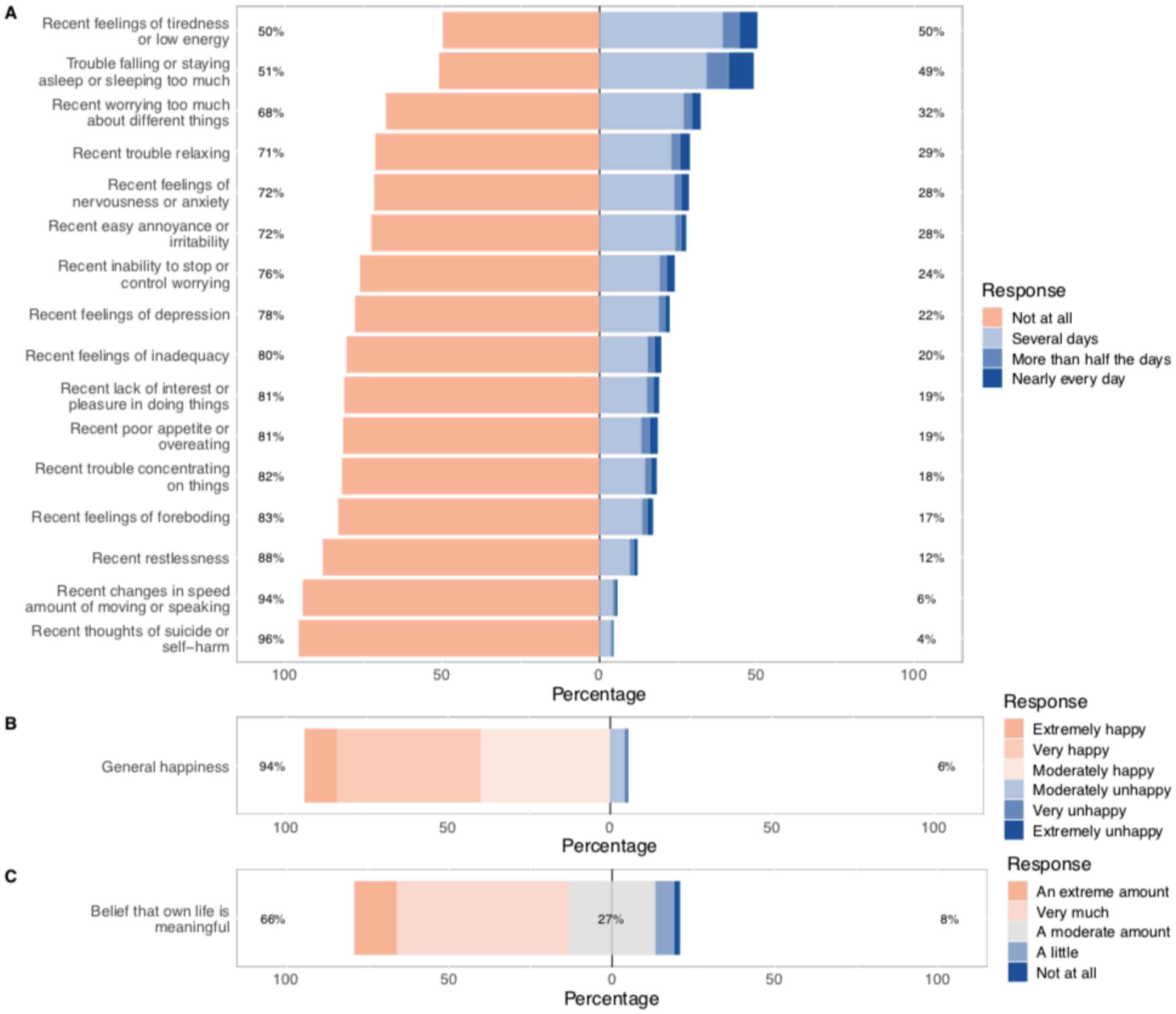

Responses to questions in the Mental Health Questionnaire are shown ("don't know" and "prefer not to answer" responses are not shown) after missing values were imputed and outliers were removed. 16 of these questions had the same coding for responses (504 coding in UK Biobank), and are displayed together as panel A. Two questions had different coding, and are displayed as panel B and C. Colours, percentages and alignment are used only for illustrative purposes in this figure; they represent the presence of depressive symptoms (orange for absence of depressive symptoms; blue for presence of depressive symptoms). Questions in panel A are in descending order of the proportion of responses that indicate the presence of depressive symptoms. Percentages labelled on the left and right side of the chart represent the total proportion of participants who responded with an orange (e.g. "not at all") or blue response respectively.

Schizophrenia phenotype

In a deviation from the pre-registered method, individuals were not removed if they were considered outliers. This was because two of the symptoms (experience of strange force trying to harm me and experience of unjust plot to harm me) had very low prevalence (1%), meaning that most individuals who responded with those symptoms would be removed during the outlier removal procedure, and the correlations between these two items could not be computed.

Bipolar disorder phenotype

Individuals' scores were calculated for a multivariate outlier detection measure, Robust Mahalanobis Distance [22]. Those who scored at or above the 99th percentile on this measure were considered outliers and were removed before further analysis. In the bipolar-disorder data, 1,356 individuals exceeded this threshold, which left N=134,250 individuals with phenotype data.

Fig S11. Responses to bipolar disorder-related questions in the Mental Health Questionnaire of the UK Biobank after imputation of missing values and removal of outliers

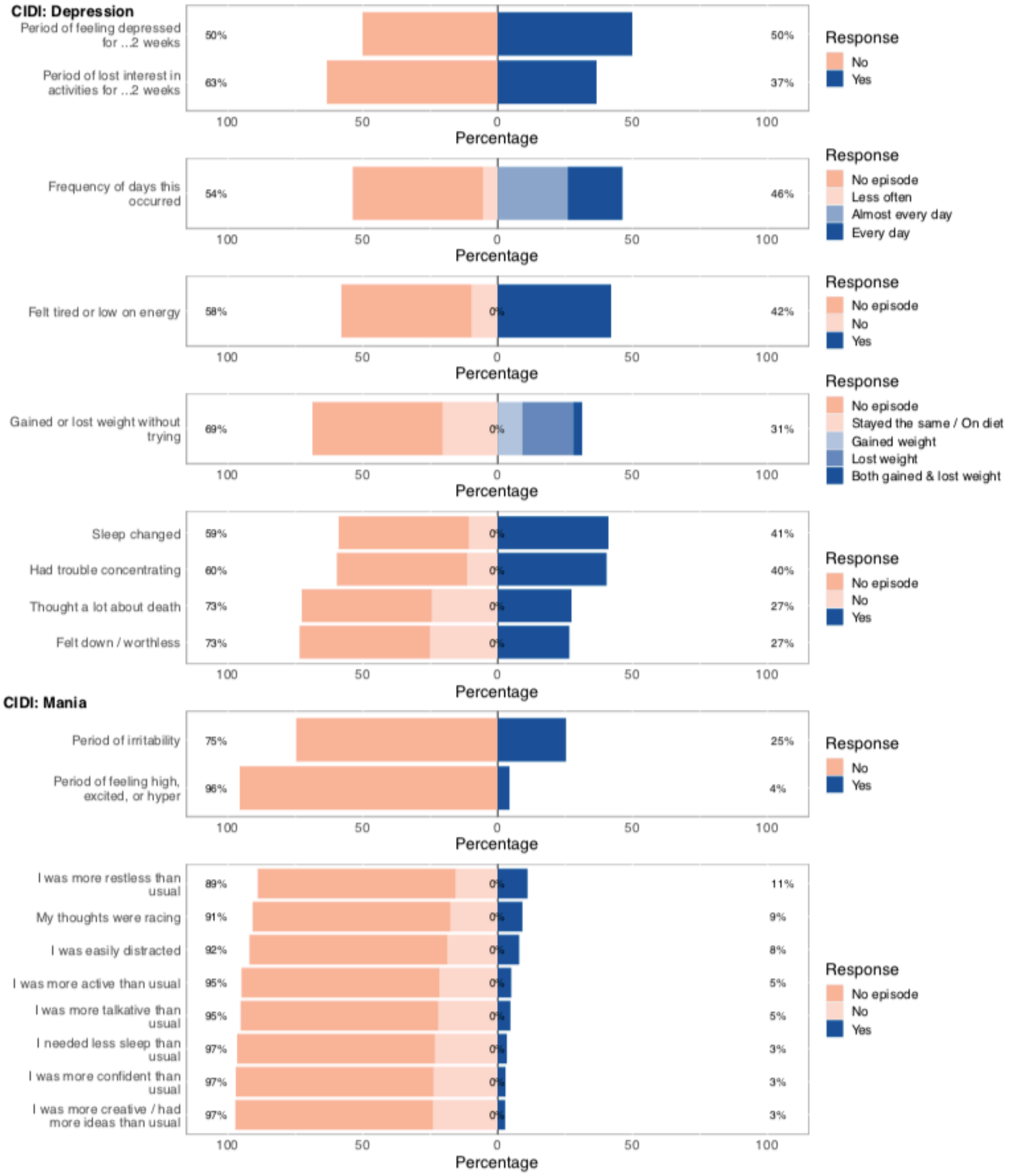

Responses to questions in the Mental Health Questionnaire are shown ("don't know" and "prefer not to answer" responses are not shown) after missing values were imputed and outliers were removed.

Negative answers to either of the leading CIDI: Mania questions (experience of a period of irritability or a period of feeling high, excited or hyper) result in a coding of “no episode” for the following questions. The no episode coding is treated equivalently to a no response in further analysis. The same coding was applied to negative answers to either of the leading CIDI: Depression questions (period of feeling depressed for 2 weeks or period of lost interest for 2 weeks) which ask about symptoms experienced during that period.

Exploratory factor analysis

Depression phenotype

Half the dataset (N = 76,846) was randomly selected to conduct ordinal exploratory factor analysis.

To assess the normality of the data, Mardia’s test of multivariate normality was conducted on a random subsample of 5,000 individuals from the dataset. The assumption of normality was rejected because the test showed the data exhibited skew (Mardia skewness=141,235, p=0) and kurtosis (Mardia kurtosis=791, p=0), so the responses were treated as ordinal in subsequent analyses.

To assess the factorability of the data, Bartlett's test of sphericity and the Kaiser-Meyer-Olkin (KMO) test were conducted. Bartlett's test of sphericity assesses whether the correlation matrix of items in the data were significantly different from an identity matrix (the null hypothesis of no underlying factor structure). The chi-squared statistic from Bartlett’s test was 1,228,597 (p = 0, df = 153), indicating factorability for the dataset. As the overall MSA from the KMO test was 0.95, this indicated factorability of the data.

In order to select the number of factors to retain, Eigenvalues were estimated and plotted as a Scree plot to estimate the number of factors that should be retained in dimensionality reduction. This suggested 3 factors should be retained (figure S6). Additionally, parallel analysis was conducted using the fa.parallel() function in the psych package, using the weighted least squares (wls) factoring method. This suggested a maximum of 6 factors should be retained. Finally, the Minimum Average Partial (MAP) criterion was calculated using the oblimin rotation method (an oblique rotation method, which allows for slight correlation between factors) and the minimum residual factoring method. The Velicer MAP criterion suggested a minimum of 3 factors to retain.

Fig S12. Scree plot showing the estimated eigenvalues of principal components calculated from the depression related questions dataset

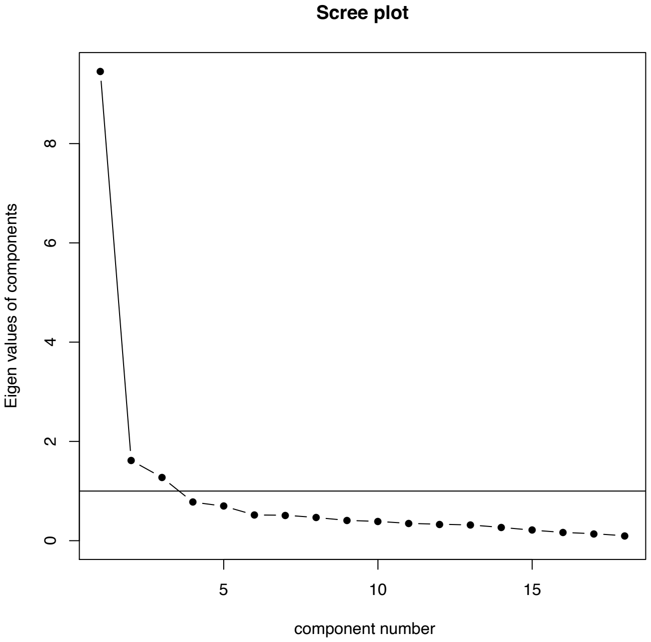

The first 18 principal components are shown, in order of the components. The eigenvalues of components decrease as the component number increases. The point of inflection (after 3 components) suggests the number of components to be retained.

In post-hoc analysis, the 6-factor model solution was adjusted by comparing the Bayesian Information Criterion (BIC) between models with varying number of items that had loadings higher than 0.3 or 0.4. The solution adjustment that was chosen was a 6-factor model solution adjusted for items that have ≥3 loadings above 0.3 or no single loading above 0.4 but retaining the items that were depression related in the PHQ-9.

The maximum Tucker-Lewis Index (TLI) and minimum Root Mean Square Error of Approximation (RMSEA) were found in a model that was reduced to 5 factors, where items with low loadings had been removed, thus removing a 6th factor. These 5 factors were therefore chosen for retention and extracted using the weighted least squares (wls) factoring method and geominQ rotation (an oblique rotation method), using the fa function in the psych package.

In this model solution, the TLI was 0.98, the RMSEA was 0.048, the BIC was 6591.66, the mean item complexity was 1.3, and the cumulative variance explained by the model was 0.72.

Figure S13. Factor structure of the chosen model solution for depression-related questions

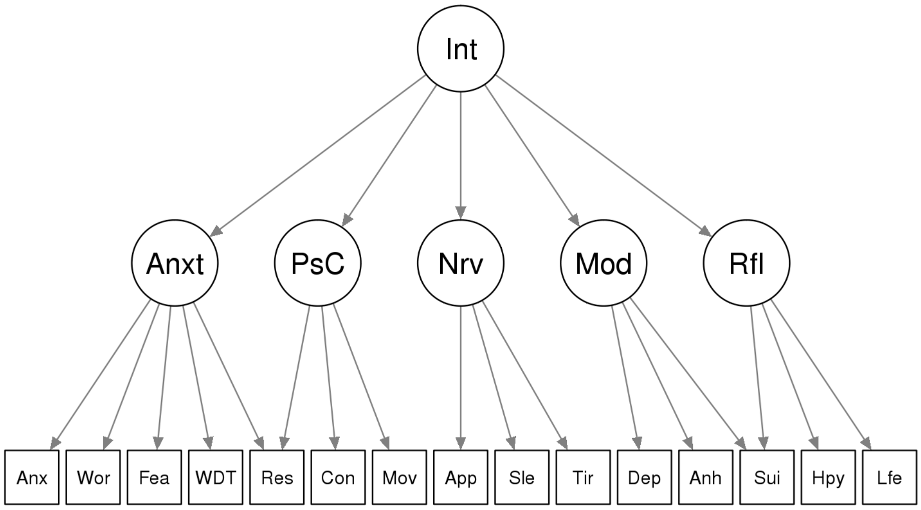

Item loadings and residuals are not depicted. Abbreviations for factor labels are as follows: Anxt = anxiety, PsC = psychomotor, Nrv = neurovegetative, Mod = mood, Rfl = reflective, Int = internalising. Abbreviations for items are as follows, described with the factors that they load onto:
Anxiety ~ Anxious/Nervous (Anx) + Worrying (Wor) + Fear awful happening (Fea) + Restless/Hard to sit still (Res) + Worrying Different Things (WDT)
Psychomotor ~ Concentration (Con) + Restless/Hard to sit still (Res) + Slow moving (Mov)
Neurovegetative ~ Poor Appetite/Overeating (App) + Falling/Staying Asleep (Sle) + Tired/Fatigue (Tir)
Mood ~ Depressed Mood (Dep) + Suicide (Sui) + Anhedonia (Anh)
Reflective ~ General Happiness (Hpy) + Unmeaningful Life (Lfe) + Suicide (Sui)

Schizophrenia phenotype

Half the dataset (N = 74,341) was randomly selected to conduct ordinal exploratory factor analysis.

To assess the factorability of the data, Bartlett's test of sphericity and the Kaiser-Meyer-Olkin (KMO) test were conducted. Bartlett's test of sphericity assesses whether the correlation matrix of items in the data were significantly different from an identity matrix (the null hypothesis of no underlying factor structure). The chi-squared statistic from Bartlett's test was 139,604.4 (p = 0, df = 66), indicating factorability for the dataset. Since Bartlett's chi-squared statistic is also a function of sample size, the KMO test statistic was evaluated as well. As the overall MSA from the KMO test was 0.77, this indicated factorability of the data.

In order to select the number of factors to retain, Eigenvalues were estimated and plotted as a Scree plot to estimate the number of factors that should be retained in dimensionality reduction. This suggested 3 or 4 factors should be retained (figure 6). Additionally, parallel analysis was conducted using the fa.parallel() function in the psych package, using the weighted least squares (wls) factoring method. This suggested a maximum of 3 factors should be retained. Finally, the Minimum Average

Partial (MAP) criterion was calculated using the oblimin rotation method (an oblique rotation method, which allows for slight correlation between factors) and the minimum residual factoring method. The Velicer MAP criterion suggested a minimum of 3 factors to retain.

Fig S14. Scree plot showing the estimated eigenvalues of principal components calculated from the schizophrenia-related questions dataset

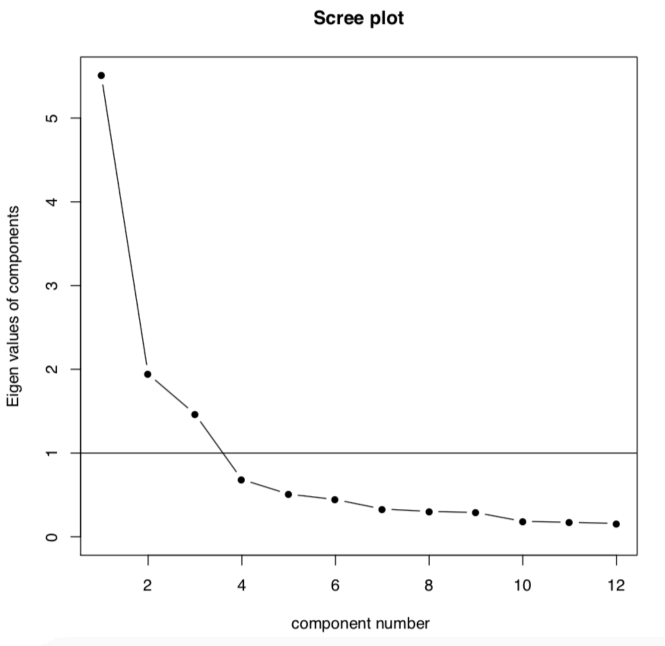

The first 12 principal components are shown, in order of the components. The eigenvalues of components decrease as the component number increases. The point of inflection (after 3-4 components) suggests the number of components to be retained.

In post-hoc analysis, the 3-factor model solution was adjusted by comparing the Bayesian Information Criterion (BIC) between models with varying number of items that had loadings higher than 0.3 or 0.4 or items that had moderate-high loadings on multiple factors. The solution adjustment that was chosen was a 3-factor model solution without the item ("I was more confident than usual"), as it had moderate-high loadings on two factors.

These 3 factors were therefore chosen for retention and extracted using the weighted least squares (wls) factoring method and geominQ rotation (an oblique rotation method), using the fa function in the psych package.

In this model solution, the TLI was 0.934, the RMSEA was 0.093, the BIC was 15969.43, the mean item complexity was 1, and the cumulative variance explained by the model was 0.7.

Figure S15. Factor structure of the chosen model solution for schizophrenia-related questions

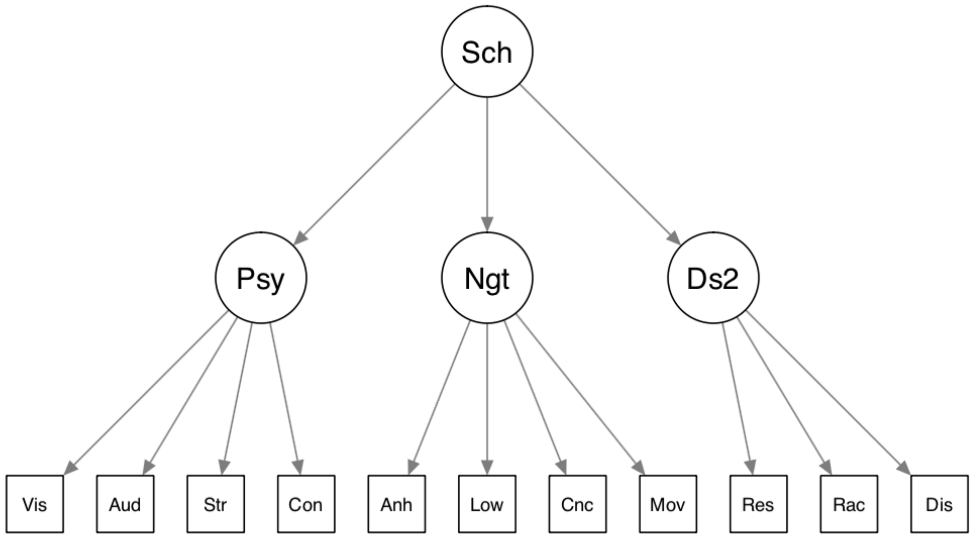

Item loadings and residuals are not depicted. Abbreviations for factor labels are as follows: Psy = psychotic, Ngt = negative, Ds2 = disorganised, Sch = general schizophrenia factor. Abbreviations for items are as follows, described with the factors that they load onto:
Psychotic ~ Visual hallucinations (Vis) + Auditory hallucinations (Aud) + Strange force (Str) + Conspiracy (Con)
Negative ~ Anhedonia (Anh) + Low energy (Low) + Trouble concentrating (Cnc) + Slow moving (Mov)
Disorganised ~ Restless/Hard to sit still (Res) + Racing thoughts (Rac) + Distracted (Dis)

Bipolar disorder phenotype

Half the dataset (N = 67,125) was randomly selected to conduct ordinal exploratory factor analysis.

To assess the factorability of the data, Bartlett's test of sphericity and the Kaiser-Meyer-Olkin (KMO) test were conducted. Bartlett's test of sphericity assesses whether the correlation matrix of items in the data were significantly different from an identity matrix (the null hypothesis of no underlying factor structure). The chi-squared statistic from Bartlett’s test was 658,159.4 (p = 0, df = 66), indicating factorability for the dataset. As the overall MSA from the KMO test was 0.93, this indicated factorability of the data.

In order to select the number of factors to retain, Eigenvalues were estimated and plotted as a Scree plot to estimate the number of factors that should be retained in dimensionality reduction. This suggested 3 or 4 factors should be retained (figure 21). Additionally, parallel analysis was conducted using the fa.parallel() function in the psych package, using the weighted least squares (wls) factoring method. This suggested a maximum of 3 factors should be retained. Finally, the Minimum Average Partial (MAP) criterion was calculated using the oblimin rotation method (an oblique rotation method, which allows for slight correlation between factors) and the minimum residual factoring method. The Velicer MAP criterion suggested a minimum of 3 factors to retain.

Fig S16. Scree plot showing the estimated eigenvalues of principal components calculated from the bipolar disorder-related questions dataset

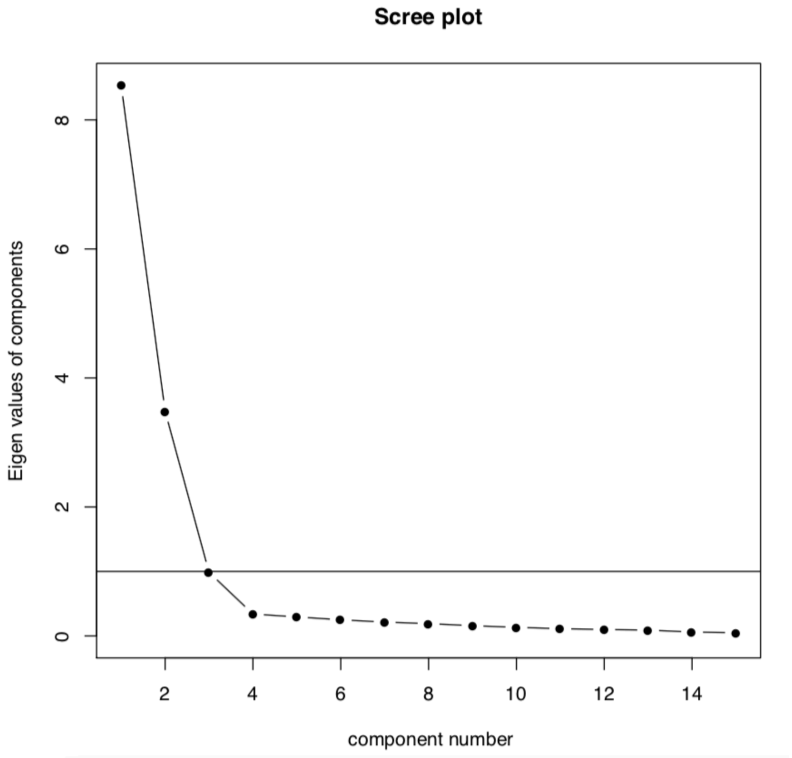

The first 15 principal components are shown, in order of the components. The eigenvalues of components decrease as the component number increases. The point of inflection (after 3-4 components) suggests the number of components to be retained.

A 3-factor model solution showed the best fit to the data, and no post-hoc adjustments were justified according to Thurstone's rules.

These 3 factors were therefore chosen for retention and extracted using the weighted least squares (wls) factoring method and geominQ rotation (an oblique rotation method), using the fa function in the psych package.

In this model solution, the TLI was 0.941, the RMSEA was 0.108, the BIC was 45880.73, the mean item complexity was 1.1, and the cumulative variance explained by the model was 0.84.

Figure S17. Factor structure of the chosen model solution for bipolar disorder-related questions

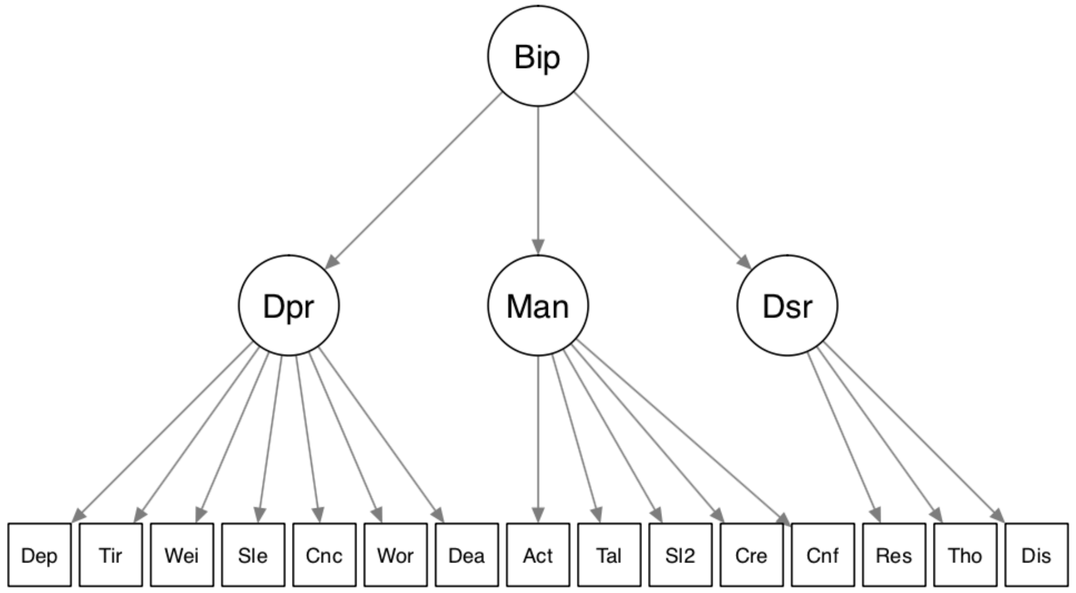

Item loadings and residuals are not depicted. Abbreviations for factor labels are as follows: Dpr = depressive, Man = manic, Dsr = disorganised, Bip = general bipolar disorder factor. Abbreviations for items are as follows, described with the factors that they load onto:
Depressive ~ Frequency of depressive symptoms (Dep) + Tired (Tir) + Weight change (Wei) + Sleep change (Sle) + Trouble concentrating (Conc) + Worthlessness (Wor) + Thoughts of death (Dea)
Manic ~ Active (Act) + Talkative (Tal) + Needed less sleep (Sl2) + Creative (Cre) + Confident (Conf)
Disorganised ~ Restless (Res) + Racing thoughts (Tho) + Distracted (Dis)

Confirmatory and hierarchical factor analysis

Depression phenotype

The model solution from exploratory factor analysis was validated using the remaining half of the dataset (N = 76,847).

In the confirmatory factor analysis, the model fit statistics were as follows. The CFI was 0.999 and the TLI was 0.998. The RMSEA was 0.023 and the Standardised Root Mean Square Residual (SRMR) was 0.026. This indicated a good model fit.

A hierarchical general factor was specified to correlate with all of the 5 factors.

Figure S18. Factor structure of the hierarchical model solution with item loadings and residual variances

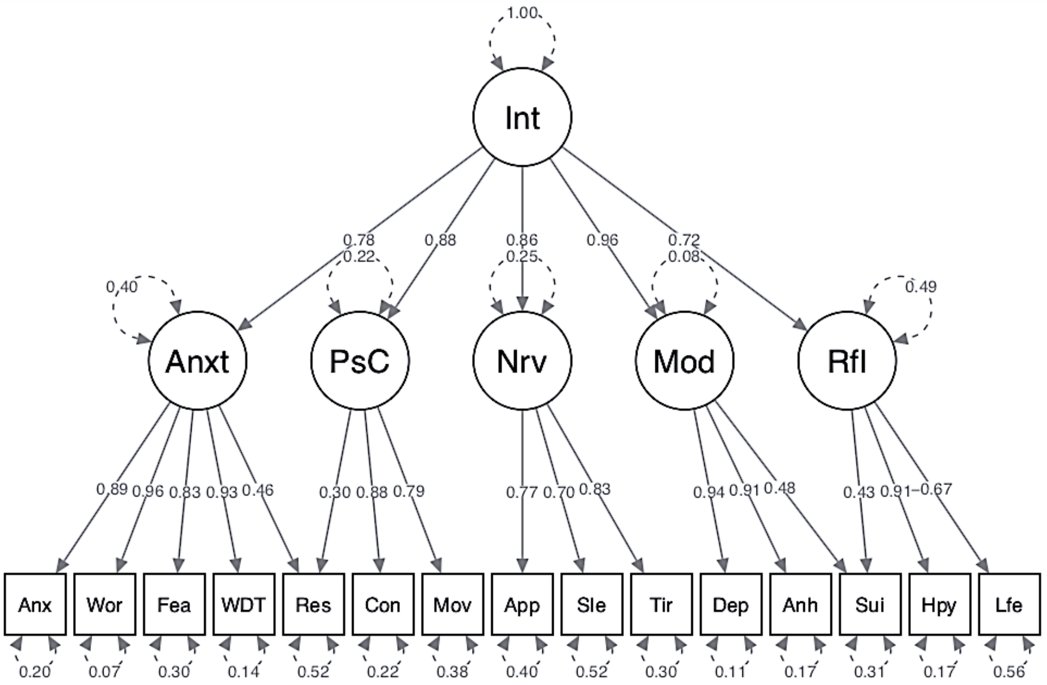

Numbers beside solid arrows depict the loadings of items/factors on other factors, while numbers beside dotted arrows depict the residual variances of items/factors. Abbreviations for factor labels are as follows: Anxt = anxiety, PsC = psychomotor, Nrv = neurovegetative, Mod = mood, Rfl = reflective, Int = internalising.

In the hierarchical model, the omega total was 0.9, and the model fit statistics were as follows. The CFI was 0.992 and the TLI was 0.990. The RMSEA was 0.039 and the SRMR was 0.031. This indicated a good model fit.

Schizophrenia phenotype

The model solution from exploratory factor analysis was validated using the remaining half of the dataset (N = 74,340).

In the confirmatory factor analysis, the model fit statistics were as follows. The CFI was 0.996 and the TLI was 0.995. The RMSEA was 0.018 and the Standardised Root Mean Square Residual (SRMR) was 0.048. This indicated a good model fit.

A hierarchical general factor was specified to correlate with all of the 3 factors.

Figure S19. Factor structure of the hierarchical model solution with item loadings and residual variances

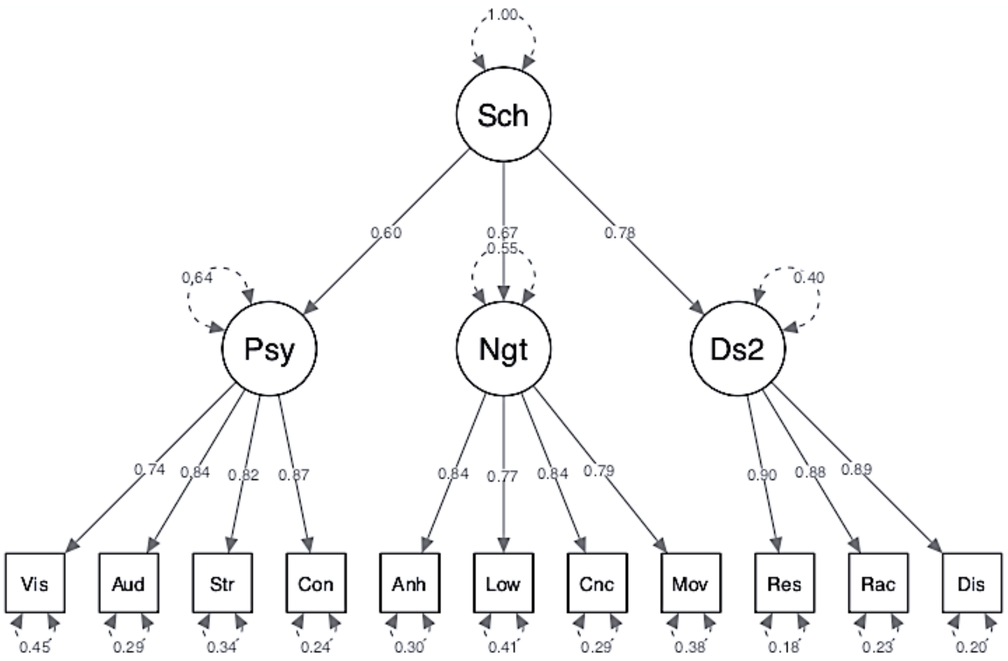

Numbers beside solid arrows depict the loadings of items/factors on other factors, while numbers beside dotted arrows depict the residual variances of items/factors. Abbreviations for factor labels are as follows: Psy = psychotic, Ngt = negative, Ds2 = disorganised, Sch = general schizophrenia factor.

In the hierarchical model, the omega total was 0.88, the explained common variance of the general factor was 0.41, and the model fit statistics were as follows. The CFI was 0.996 and the TLI was 0.995. The RMSEA was 0.018 and the SRMR was 0.048. This indicated a good model fit.

Bipolar disorder phenotype

The model solution from exploratory factor analysis was validated using the remaining half of the dataset (N = 67,124).

In the confirmatory factor analysis, the model fit statistics were as follows. The CFI was 0.999 and the TLI was 0.999. The RMSEA was 0.024 and the Standardised Root Mean Square Residual (SRMR) was 0.045. This indicated a good model fit.

A hierarchical general factor was specified to correlate with all of the 3 factors.

Figure S20. Factor structure of the hierarchical model solution with item loadings and residual variances

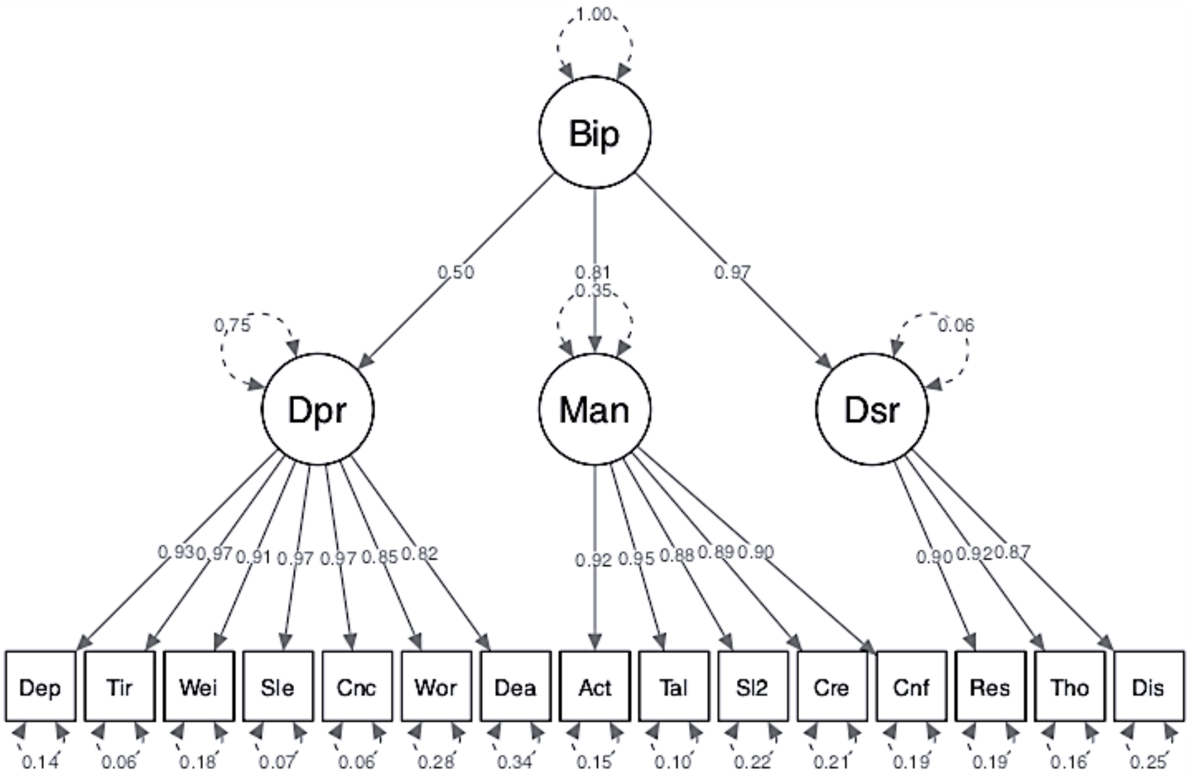

Numbers beside solid arrows depict the loadings of items/factors on other factors, while numbers beside dotted arrows depict the residual variances of items/factors. Abbreviations for factor labels are as follows: Dpr = depressive, Man = manic, Dsr = disorganised, Bip = general bipolar disorder factor.

In the hierarchical model, the omega total was 0.93, the explained common variance of the general factor was 0.73, and the model fit statistics were as follows. The CFI was 0.999 and the TLI was 0.999. The RMSEA was 0.023 and the SRMR was 0.044. This indicated a good model fit.

Individuals' scores on all the factors were calculated and plotted (shown below). Scores on the hierarchical factor (termed the “internalising factor”) were used as the continuous phenotype in genetic association testing that followed.

Factor scoring

Depression phenotype

Figure S21. Distribution of individuals’ scores on all factors in the hierarchical model solution for depression

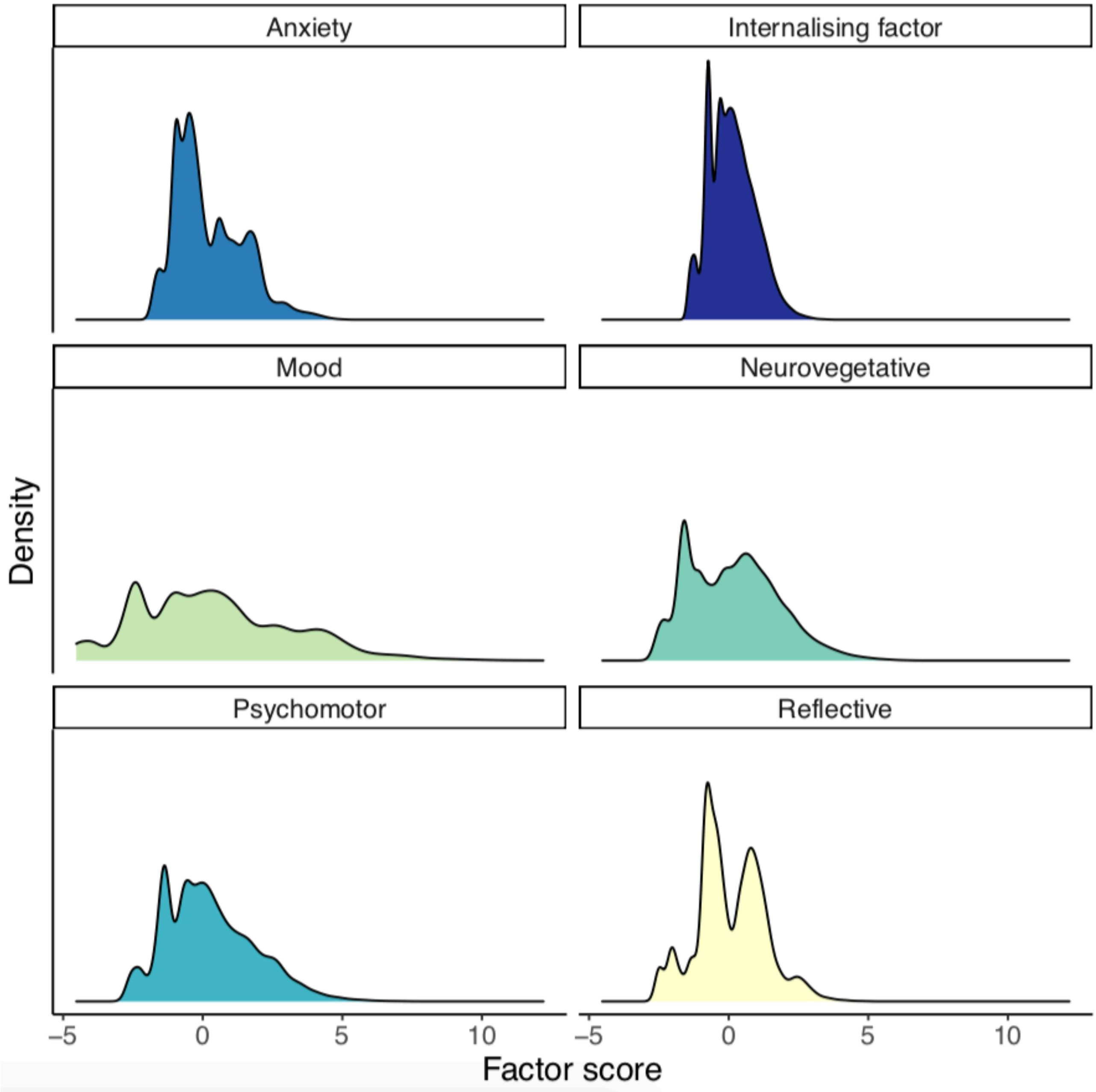

Density plots are shown to illustrate the distributions of individuals' scores on each factor of the hierarchical model solution. Latent factor scores for individuals were estimated using the empirical Bayes method in Lavaan.

Individuals' scores on all the factors were calculated and plotted (shown below). Scores on the hierarchical factor (termed the general schizophrenia factor) were used as the continuous phenotype in genetic association testing that followed.

Schizophrenia phenotype

Figure S22. Distribution of individuals' scores on all factors in the hierarchical model solution for schizophrenia

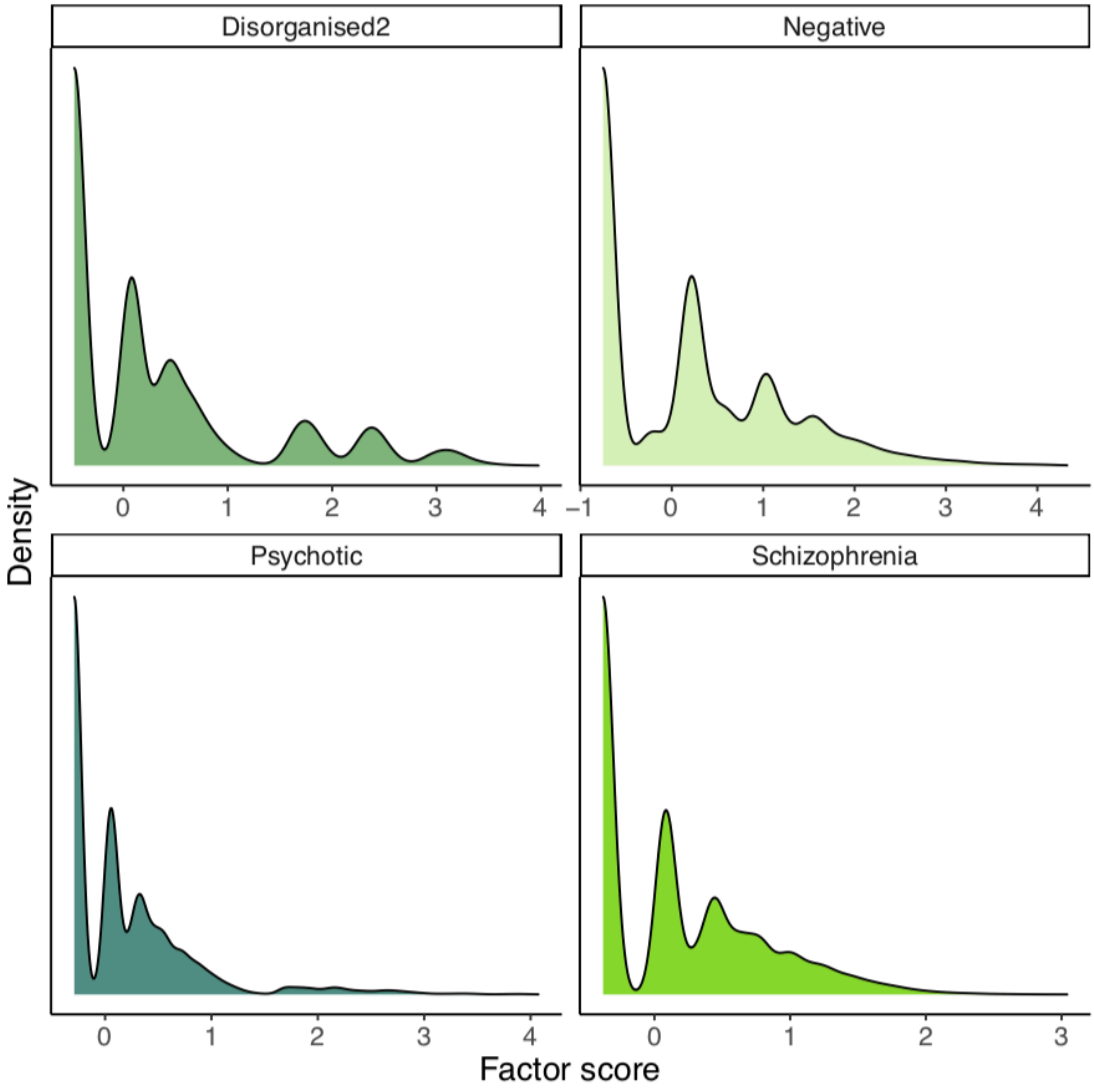

Density plots are shown to illustrate the distributions of individuals' scores on each factor of the hierarchical model solution. Latent factor scores for individuals were estimated using the empirical Bayes method in Lavaan.

Individuals' scores on all the factors were calculated and plotted (shown below). Scores on the hierarchical factor (termed the general bipolar disorder factor) were used as the continuous phenotype in genetic association testing that followed.

Bipolar disorder phenotype

Figure S23. Distribution of individuals' scores on all factors in the hierarchical model solution for bipolar disorder

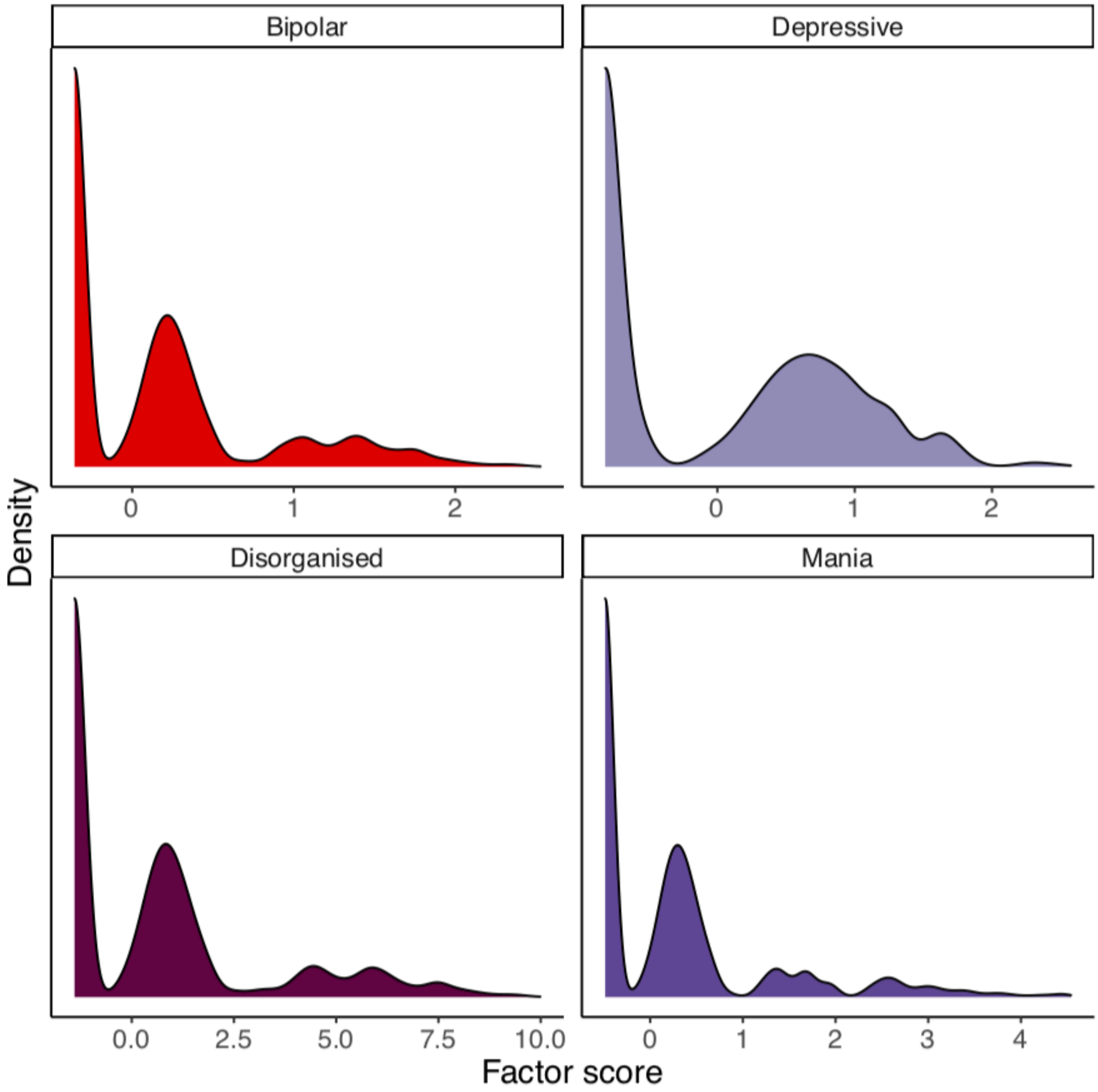

Density plots are shown to illustrate the distributions of individuals' scores on each factor of the hierarchical model solution. Latent factor scores for individuals were estimated using the empirical Bayes method in Lavaan.

Sample size and removals due to exclusions and genotype QC procedures

Table S6. Sample size and removals due to missing values, outlier exclusions, genotype QC procedure and missing covariate information

	Major depression	Bipolar disorder	Schizophrenia
Initial sample size of Mental Health Questionnaire	157 366		
Removed due to remaining missing values after imputation	2 120	21 760	8 685
Removed due to outlier removal procedure	1 553	1 357	0
Sample size used in factor analysis	*153 693*	*134 249*	*148 681*
Removed due to genotype QC procedure	19 225	16 868	18 663
Removed due to missing covariate information	5	5	5
Final sample size used in genetic analysis	***134 463***	***117 376***	***130 013***

Table S7. Sample demographics of final sample used in genetic analysis

	**Major depression**	**Bipolar disorder**	**Schizophrenia**
Age at recruitment (range)	38 – 72	38 – 72	38 – 72
Age at recruitment (median)	57	57	57
N female (%)	75 451 (56.1%)	65 166 (55.6%)	73 194 (56.3%)
N male (%)	59 012 (43.9%)	52 210 (44.4%)	56 819 (43.7%)
Townsend deprivation index (range)	-6.258 – 11.001	-6.258 – 11.001	-6.258 – 11.001
Townsend deprivation index (median)	-2.487	-2.487	-2.509

Table S8. Number of genes, single variants and pathways tested

	Major depression	Bipolar disorder	Schizophrenia
Number of genes tested in gene-based association analysis	9 964	9 912	9 954
Number of single variants tested in GWAS	13 921 407	13 911 892	13 933 876
Number of GO pathways tested in pathway analysis	5 916	5 915	5 916

Table S9. Number of single variants retained at each step of QC

Initial number of variants	**39 248 847**	
Removed due to missing genotype data	--geno 0.02	1 678 298
Removed due to Hardy Weinberg equilibrium test	--hwe 0.00000001	18 790
Remaining for annotation and GWAS	**37 551 759**	
Removed due to MAF (0.0005) in GWAS for single variant association testing	Depression	23 630 352
	Bipolar disorder	23 639 867
	Schizophrenia	23 617 883

Figure S24. Number of genes contained in each OMIM locus associated with matched phenotypes

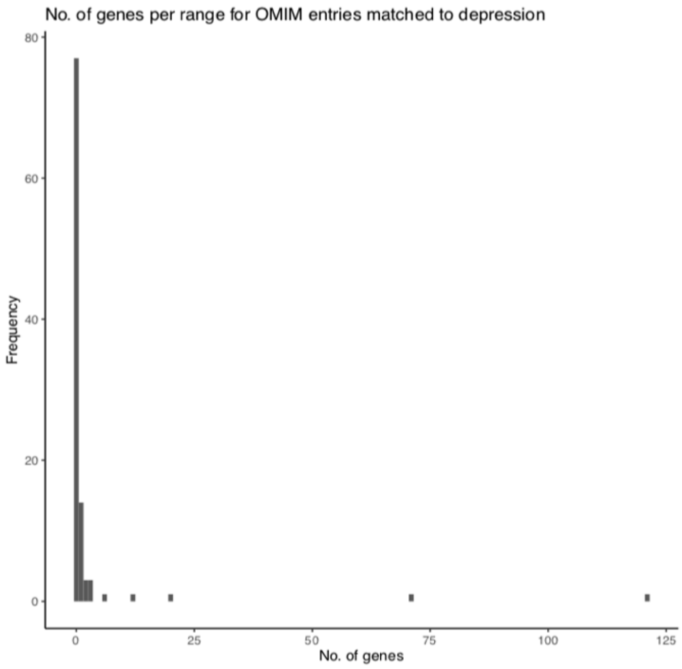

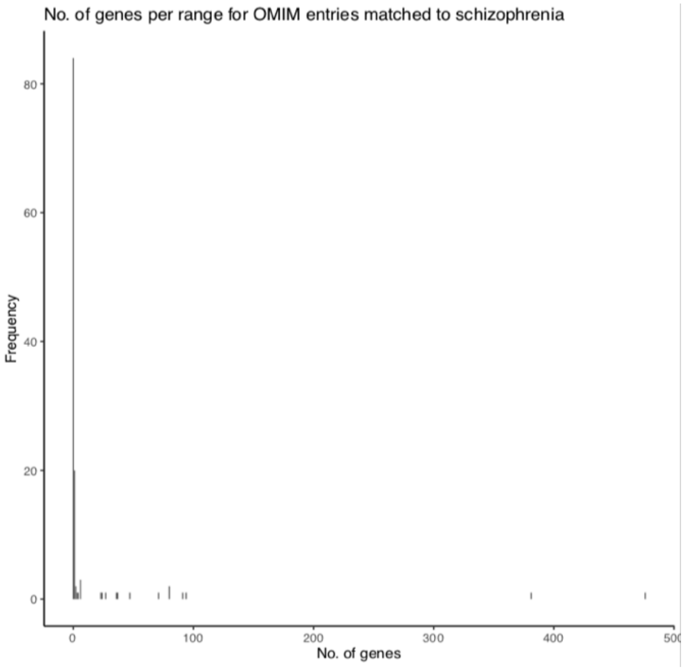

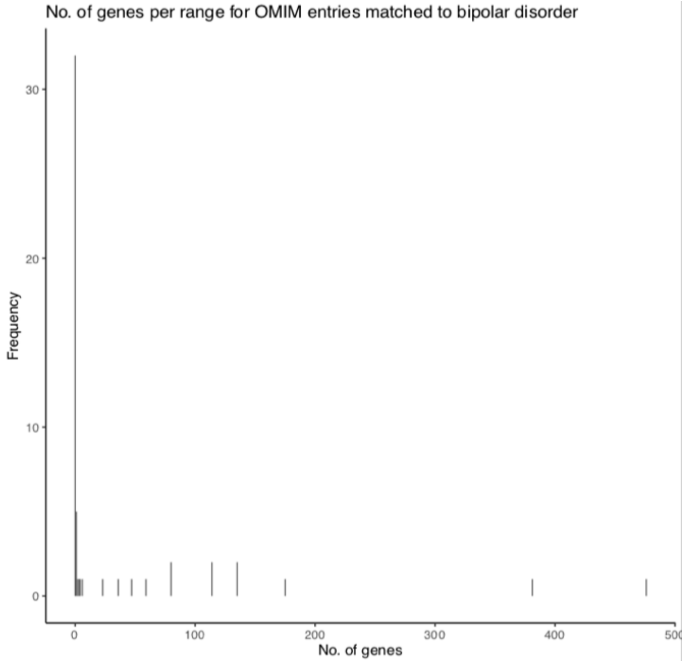

GWAS of single nucleotide variants

Table S10. Top 30 single variant associations with the general internalising factor of depression

CHR	SNP	POS	A1	A2	N	AF1	BETA	SE	P
8	rs7843140	4924197	A	C	132095	0.493758	-0.0176741	0.00313946	1.81E-08
3	rs12492113	50521402	A	G	132632	0.121818	0.0269267	0.00478315	1.81E-08
19	rs138834118	18595822	T	C	133577	0.00559602	0.115939	0.0209031	2.91E-08

3	rs1467915	50523498	C	G	132036	0.130711	0.0257624	0.00465022	3.02E-08
10	rs4748978	25052109	C	T	133220	0.353322	0.0180819	0.00326941	3.19E-08
9	rs116524077	9908082	A	C	132551	0.0191473	0.0630338	0.0114345	3.54E-08
3	rs35684775	50527549	G	T	132685	0.131797	0.0252221	0.00462232	4.85E-08
8	rs6558961	4916964	C	T	133051	0.469632	0.0170389	0.0031329	5.37E-08
11	rs7127006	16379226	A	G	133719	0.276917	0.0189437	0.00349011	5.70E-08
3	rs1919565	82135381	T	A	134402	0.10594	-0.0274096	0.00505295	5.81E-08
3	rs28393435	82139065	C	T	134372	0.106179	-0.0273793	0.00504898	5.87E-08
3	rs58432784	50527218	A	G	132722	0.13176	0.0250647	0.00462229	5.87E-08
3	rs35829757	50528212	T	C	132721	0.131758	0.0250319	0.00462233	6.11E-08
10	rs2152432	25048987	T	G	133501	0.352937	0.0176788	0.00326603	6.20E-08
3	rs6807916	50532006	G	A	132727	0.131755	0.0250057	0.00462226	6.31E-08
3	rs34445262	82142675	GT	G	134257	0.10598	-0.0273383	0.00505494	6.36E-08
3	rs9849917	82148677	C	T	134340	0.106063	-0.0273065	0.00505228	6.49E-08
5	rs186566061	9689689	T	C	133866	0.0020655	0.185406	0.0343629	6.83E-08
3	rs1467916	50523494	G	T	132723	0.131993	0.0248929	0.00461764	7.01E-08
3	rs35053615	50534129	T	G	132747	0.131649	0.024909	0.00462344	7.14E-08
10	10:25036373_CTTTT_C	25036373	CTTTT	C	132744	0.325491	0.0179907	0.00333985	7.18E-08
3	rs28482395	82153545	G	C	134344	0.105915	-0.0272262	0.00505489	7.20E-08
3	rs35986136	50535221	T	C	132750	0.131642	0.0249001	0.00462362	7.23E-08
3	rs7613931	50534350	G	A	132747	0.131705	0.0248926	0.0046226	7.25E-08
3	rs73835511	50532900	T	C	132748	0.131712	0.0248853	0.00462237	7.30E-08
12	rs71079554	109816107	CT	C	133217	0.299215	0.018357	0.00341008	7.32E-08
8	rs4875439	4918350	A	G	133224	0.46942	0.0168452	0.0031307	7.42E-08
3	rs9857763	82145288	C	T	134360	0.106081	-0.0271708	0.00505149	7.50E-08
10	rs10828698	25039389	C	T	134463	0.353971	0.017486	0.00325276	7.63E-08
3	rs7613786	82156524	T	C	134012	0.105311	-0.0272595	0.00507235	7.70E-08

Table S11. Top 30 single variant associations with the general factor of schizophrenia

CHR	SNP	POS	A1	A2	N	AF1	BETA	SE	P
1	rs72657988	35688541	T	G	130013	0.0823841	0.0259828	0.00425436	1.01E-09
12	rs4759275	57525756	A	G	130013	0.421139	-0.013538	0.00237253	1.16E-08
12	rs12322902	57513866	G	A	127924	0.422352	-0.0134887	0.00239094	1.68E-08
12	rs10876963	57514554	C	T	127914	0.422682	-0.0134698	0.0023907	1.76E-08

12	rs11172111	57515804	G	T	127956	0.42236	-0.0134505	0.00239066	1.84E-08
12	rs12298170	57515363	G	A	127960	0.422327	-0.0134363	0.00239065	1.91E-08
12	rs10467124	57519694	C	G	127990	0.422217	-0.0133653	0.00239074	2.26E-08
18	rs568027440	41430789	C	T	129414	0.00369357	-0.10703	0.0193586	3.22E-08
1	rs77648004	35765084	G	A	129982	0.0392054	0.0332083	0.0060245	3.54E-08
1	rs78201023	35771901	T	C	130013	0.0393499	0.0330162	0.00601352	4.01E-08
1	rs112802957	35745678	T	A	129003	0.0472935	0.0298311	0.00552659	6.75E-08
12	rs4759276	57526646	A	G	128831	0.387438	-0.0130014	0.00241655	7.44E-08
1	rs113859277	35932718	C	A	129458	0.0483555	0.0290705	0.00545705	9.98E-08
1	rs2279681	201861016	G	C	130013	0.344481	-0.0130916	0.00246148	1.05E-07
12	rs324013	57510661	C	T	127768	0.481451	0.0125705	0.00236384	1.05E-07
3	rs1467915	50523498	C	G	127661	0.130772	0.0185822	0.0035013	1.11E-07
12	rs2122692	57510511	C	G	127747	0.481467	0.0125363	0.00236397	1.14E-07
9	rs117689876	138012630	T	G	129256	0.0156666	-0.0497384	0.00943768	1.36E-07
5	rs35514087	136032553	G	A	128417	0.0348124	0.0336128	0.00642969	1.72E-07
12	rs11172106	57512875	G	C	127823	0.449258	-0.0123366	0.00237348	2.02E-07
5	rs201737663	136035261	G	GTGTGCATATGC	127415	0.0352588	0.0330475	0.00641376	2.57E-07
3	rs12492113	50521402	A	G	128232	0.121869	0.0185337	0.00359971	2.62E-07
12	rs12312693	57511734	C	T	127881	0.449258	-0.012212	0.00237283	2.65E-07
3	rs35908181	50541915	C	T	128438	0.133598	0.0176868	0.00345852	3.15E-07
3	rs34762184	50541769	A	G	128451	0.133572	0.0176803	0.00345855	3.19E-07
3	rs35684775	50527549	G	T	128280	0.131841	0.0177658	0.00348058	3.32E-07
14	rs6572124	42486150	T	C	129964	0.328641	0.0126804	0.00249239	3.63E-07
3	rs58432784	50527218	A	G	128316	0.131804	0.0177038	0.00348055	3.65E-07
3	rs61423002	50537048	G	A	128299	0.133559	0.0175957	0.00346107	3.70E-07
3	rs35829757	50528212	T	C	128315	0.131801	0.0176733	0.00348058	3.82E-07

Table S12. Top 30 single variant associations with the general factor of bipolar disorder

CHR	SNP	POS	A1	A2	N	AF1	BETA	SE	P
2	rs62124876	22661763	C	A	116925	0.0809964	-0.0266541	0.0047861	2.56E-08
1	rs11809819	190672185	C	G	115806	0.371211	0.0146822	0.00271343	6.27E-08
1	rs16832656	190675536	A	G	115796	0.371269	0.0146835	0.00271369	6.27E-08
1	rs1408838	190671289	C	T	115788	0.371226	0.0146679	0.00271365	6.47E-08
1	rs6675319	190671240	C	T	115788	0.371226	0.0146676	0.00271365	6.48E-08
1	rs7514760	190667609	C	A	115803	0.371161	0.0146222	0.0027137	7.11E-08
4	rs62340589	176875795	C	G	115860	0.200777	0.0175803	0.00327351	7.85E-08

4	rs11733419	176863487	T	C	117061	0.203992	0.017371	0.00323885	8.17E-08
20	rs117271682	55034866	A	G	116590	0.00640278	0.0880293	0.0164287	8.40E-08
4	rs62334821	176865038	A	G	116851	0.1923	0.0177055	0.0033142	9.18E-08
2	rs62124875	22659091	A	G	117376	0.0800036	-0.0256685	0.00480475	9.18E-08
4	rs41533650	176869252	A	G	116788	0.21247	0.0170517	0.00319278	9.26E-08
4	rs62340585	176871122	A	G	116727	0.212487	0.0170034	0.00319353	1.01E-07
4	rs2333321	176859992	A	G	117002	0.204902	0.0172153	0.00323544	1.03E-07
6	rs574288543	141810262	A	T	117195	7.51E-04	0.249107	0.047675	1.74E-07
4	4:176874326_CAAAT_C	176874326	C	CAAAT	116043	0.21137	0.016768	0.00321	1.75E-07
4	rs62334820	176855221	T	C	116384	0.198846	0.0170267	0.00328025	2.10E-07
9	rs186207413	119396441	T	A	116713	0.00460103	0.100231	0.019338	2.18E-07
6	rs543185062	140798866	A	G	117220	6.44E-04	0.266529	0.0514651	2.23E-07
7	rs57506017	12245225	T	A	116495	0.291283	0.0148772	0.0028798	2.39E-07
17	rs2435204	43988205	G	A	116696	0.229074	0.016055	0.00310781	2.39E-07
7	rs17165701	12239274	C	T	116025	0.290511	0.0148768	0.00288812	2.59E-07
4	rs78825642	65473340	G	C	117376	0.123185	-0.0203508	0.00395813	2.73E-07
12	rs61935210	92817310	T	C	115934	0.276105	0.0150679	0.00293397	2.81E-07
7	rs56761518	12252119	G	GT	115105	0.387398	0.0138287	0.00269876	2.99E-07
4	4:176866840_CTT_C	176866840	C	CTT	116511	0.202543	0.0166792	0.00325604	3.01E-07
17	rs916888	44863133	C	T	117376	0.245945	0.0154524	0.00302413	3.23E-07
17	rs62063264	44033401	T	C	116626	0.220414	0.0160329	0.00315405	3.71E-07
17	rs9891103	44091886	T	C	117007	0.227085	0.015817	0.00311331	3.77E-07
8	rs76483837	136009464	T	C	116883	0.00167261	0.16196	0.0320157	4.22E-07

Genomic inflation and QQ plots

To derive the number of cases in the continuous phenotypes (to calculate the lambda 1000), we calculated the number of participants with a phenotype score above one standard deviation of the mean.

Table S13. Genomic inflation factor for single variant association tests (standard errors are shown in brackets)

	Int factor	Bip factor	Sch factor
Lambda	1.089795	1.077558	1.087371
Lambda 1000	1.002397	1.002295	1.002334
LDSC intercept	1.0045 (0.0081)	1.0073 (0.007)	1.0074 (0.0086)

Figure S25. QQ plot depicting the expected versus observed $-\log_{10}$(p-value) of association test statistics for single variants with (a) the internalising factor of depression (Int), (b) the general factor of schizophrenia (Sch), (c) the general factor of bipolar disorder (Bip)

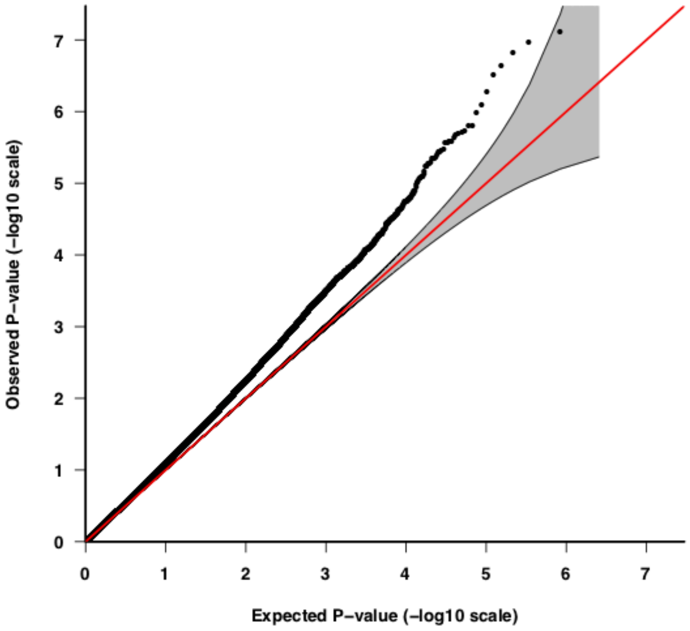
Observed P−value (−log10 scale)
Expected P−value (−log10 scale)
0
1
2
3
4
5
6
7

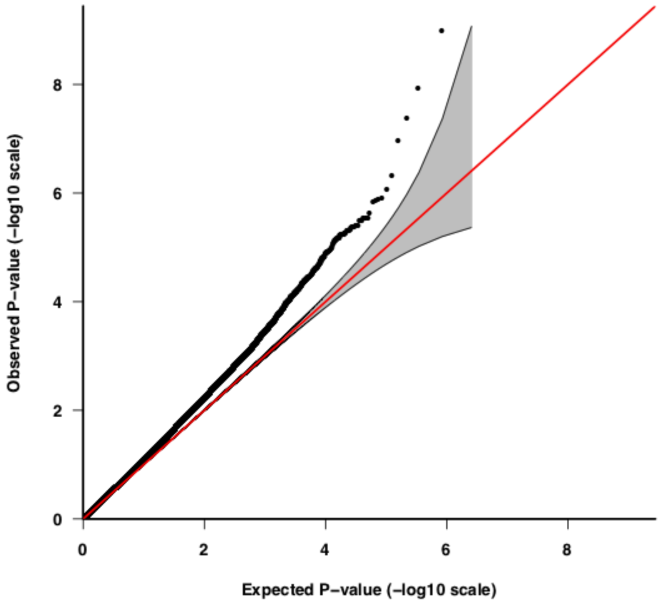
Observed P−value (−log10 scale)
Expected P−value (−log10 scale)
0
2
4
6
8

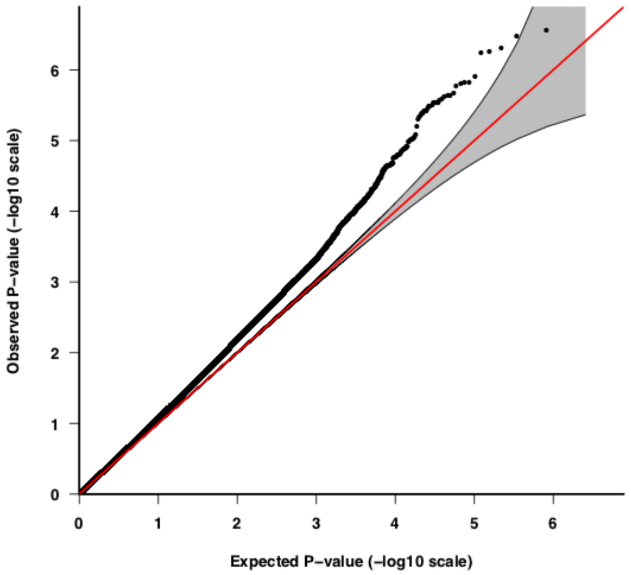
Observed P−value (−log10 scale)
Expected P−value (−log10 scale)
0
1
2
3
4
5
6

The QQ plot depicts the deviation of observed p-values from their distribution under a null hypothesis (shown as the red diagonal line), with a 95% confidence interval depicted in grey bounds.

QQ plots stratified by MAF

Figure S26. QQ plot depicting the expected versus observed –log$_{10}$(p-value) of association test statistics for single variants with the general internalising factor of depression (stratified by MAF)

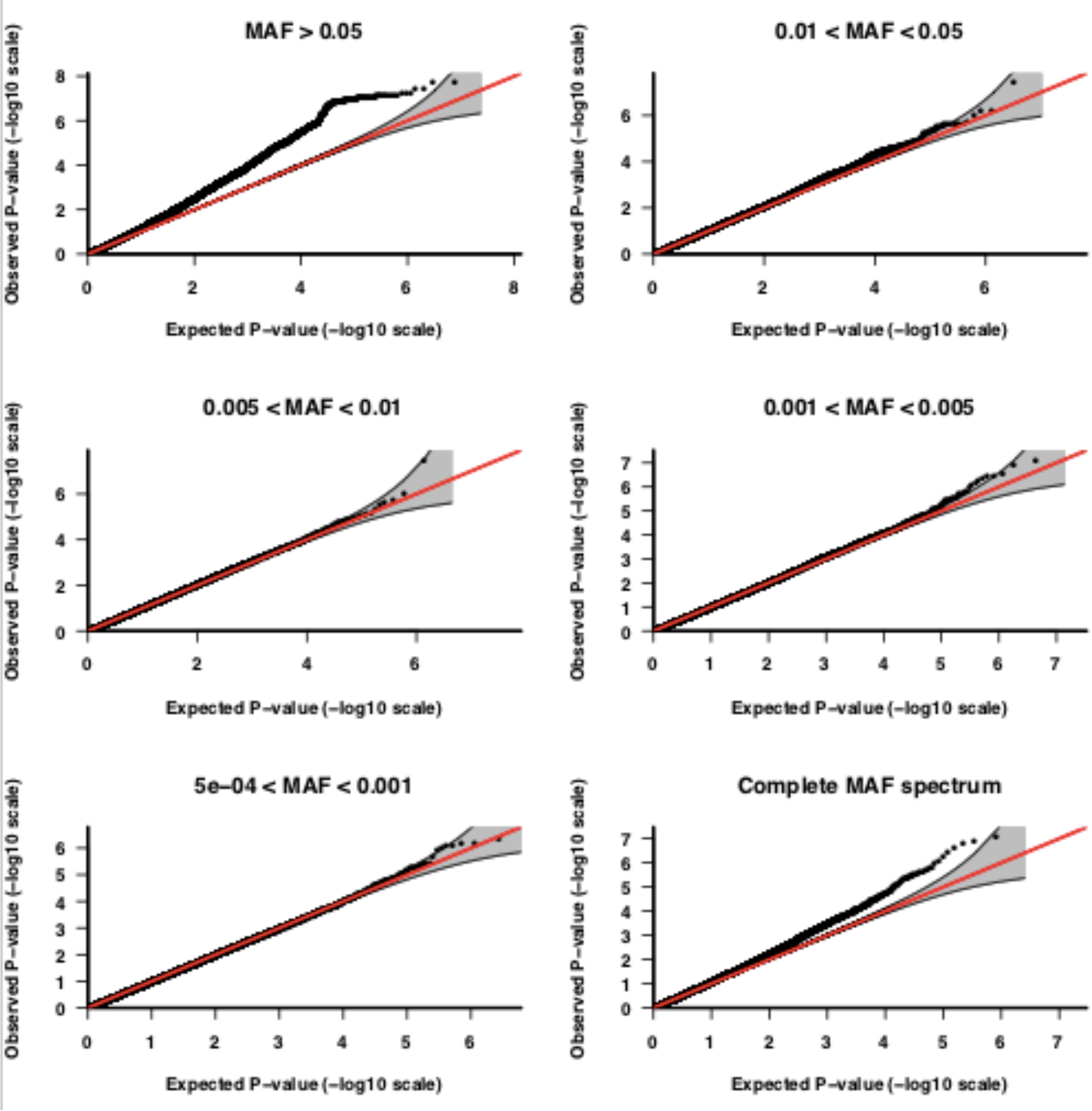

Figure S27. QQ plot depicting the expected versus observed –log$_{10}$(p-value) of association test statistics for single variants with the general factor of schizophrenia (stratified by MAF)

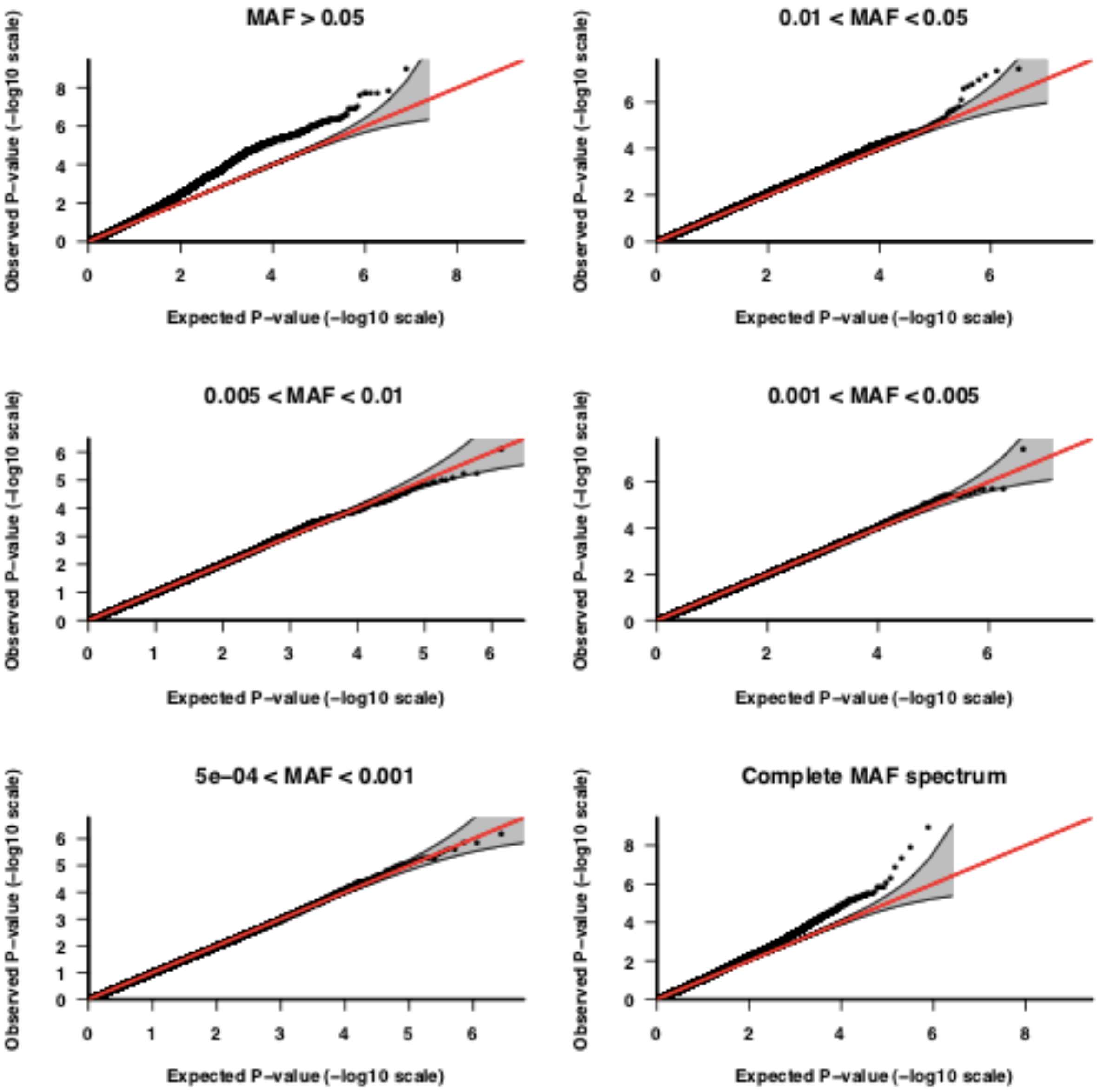

Figure S28. QQ plot depicting the expected versus observed –$\log_{10}$(p-value) of association test statistics for single variants with the general factor of bipolar disorder (stratified by MAF)

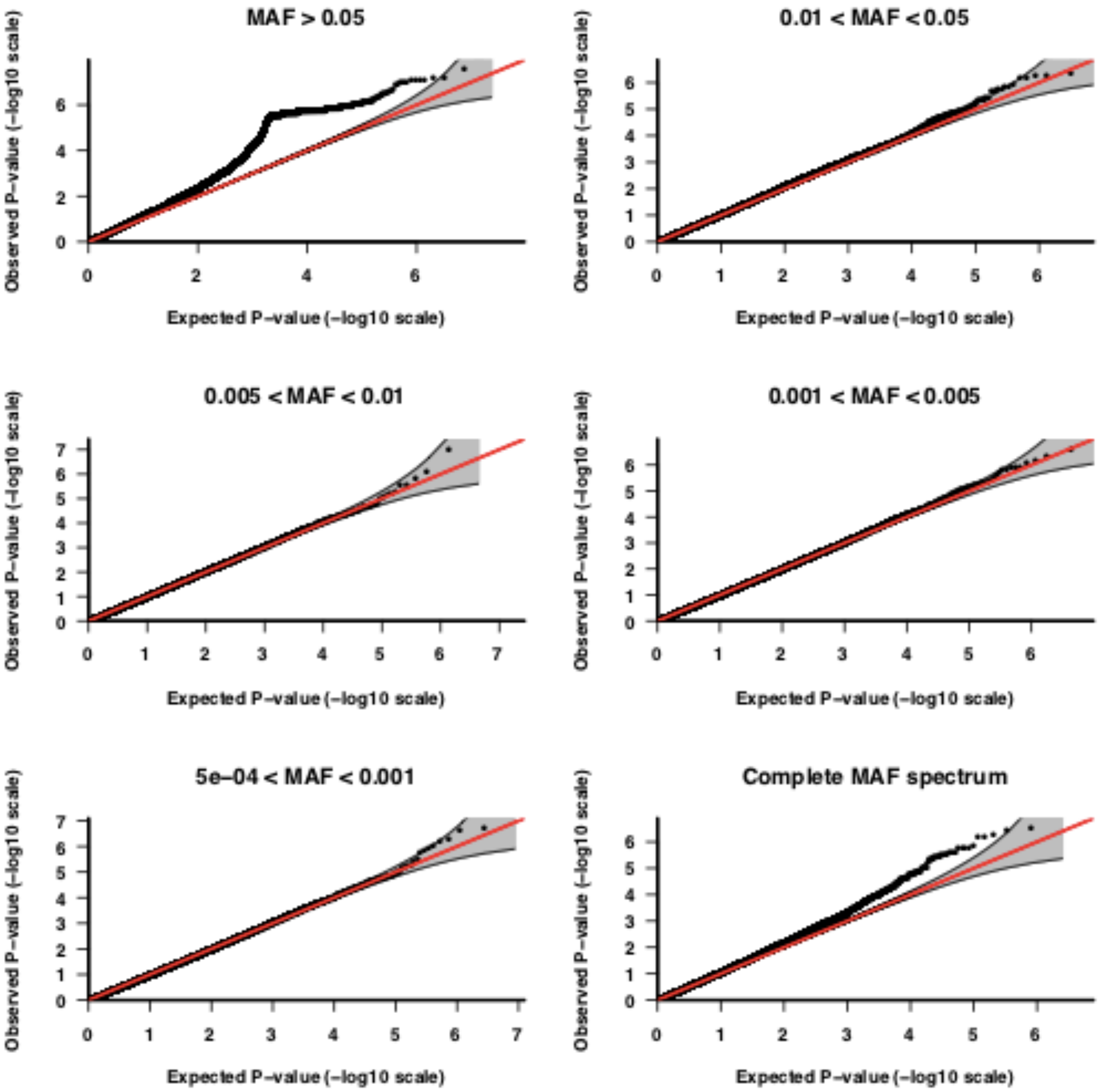

Functional annotations of variants

Tables S14-16. Tables describing the number and percentages of functional annotations of variants

MutationTaster prediction	Number of variants	Percentage of variants
A (disease causing automatic – known to be deleterious in dbSNP)	1,388	0.0061550%
D (disease causing)	50,420	0.2235837%
N (polymorphism; probably harmless)	46,702	0.2070965%

P (polymorphism automatic; known to be harmless in dbSNP)	7,338	0.0325398%
NA	22,444,990	99.5306250%

**Prediction score**	**Min.**	**1st Qu.**	**Median**	**Mean**	**3rd Qu.**	**Max**	**NA's**
SIFT score	0.0	0.008	0.0830	0.2124697	0.3090	1.00	22,450,339
GERP++	-12.3	0.688	3.4400	2.3196194	4.9600	6.17	22,442,561
FATHMM	0.0	0.000	0.0030	0.1357585	0.1420	1.00	22,463,293
dbscSNV ADA	0.0	0.000	0.0002	0.1576923	0.0078	1.00	22,529,016
dbscSNV RF	0.0	0.002	0.0240	0.1711592	0.1620	1.00	22,529,016

**Deleterious category**	**Number of variants**	**Percentage of variants**
Missense / indel	14,099	0.0625199%
Non-coding	30	0.0001330%
None	22,529,990	99.9057967%
Protein-truncating	3,774	0.0167352%
Splicing	3,341	0.0148152%

**Exonic ensGene**	**Number of variants**	**Percentage of variants**
Frameshift deletion	741	0.0032859%
Frameshift insertion	321	0.0014235%
Non-frameshift deletion	631	0.0027981%
Non-frameshift insertion	265	0.0011751%
Non-synonymous single nucleotide variant	106,881	0.4739558%
Startloss	389	0.0017250%
Stopgain	2,227	0.0098755%
Stoploss	207	0.0009179%
Synonymous single nucleotide variant	77,049	0.3416680%
Unknown	1,912	0.0084786%
NA	22,360,215	99.1546966%

Numbers (n) and percentages (n_pct) of variants with each functional prediction (Exonic_ensGene, SIFT score, GERP++, FATHMM, dbscSNV ADA and dbscSNV RF) are shown, along with the final number and percentages of variants that were categorised as predicted deleterious variants in each category (variants labelled with 'none' refer to variants that were not predicted to be predicted deleterious variants).

Figure S29. Density plot illustrating the distribution of functional annotations of variants

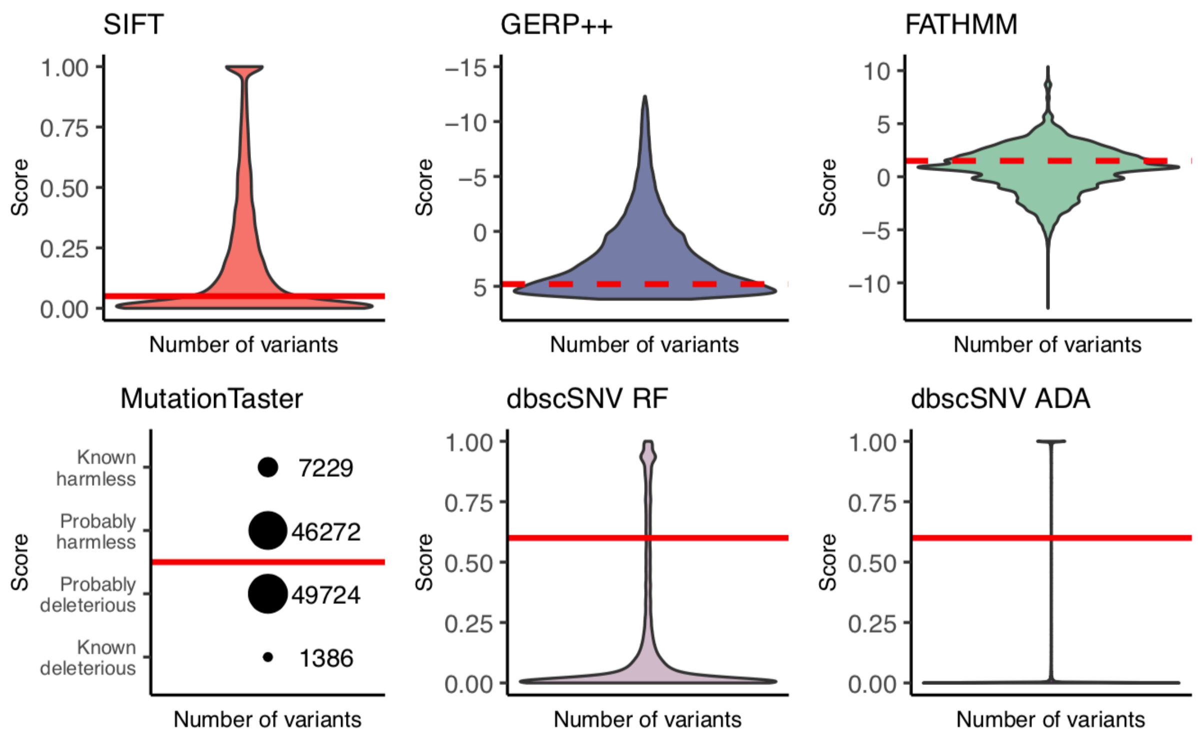

The distribution of scores on 6 functional annotation tools are depicted above: MutationTaster [40], *GERP++* [41], *FATHMM* [42], *SIFT* [43], *dbscSNV RF and dbscSNV ADA* [39]. *MutationTaster annotates variants into categories, while the other tools annotate variants with numerical scores. Authors of respective papers suggest that variants with scores ≥4.8 on GERP++, ≤1.5 on FATHMM, <0.05 on SIFT, <0.6 on dbscSNV RF or ADA, and "probably deleterious" or "known deleterious" on MutationTaster should be considered deleterious. These thresholds are depicted with a red line (dashed if the threshold is inclusive of the threshold score). For each annotation tool, variants below that threshold are considered deleterious.*

Gene-based association testing

Figure S30. Number of SNPs per gene tested in gene-based burden tests

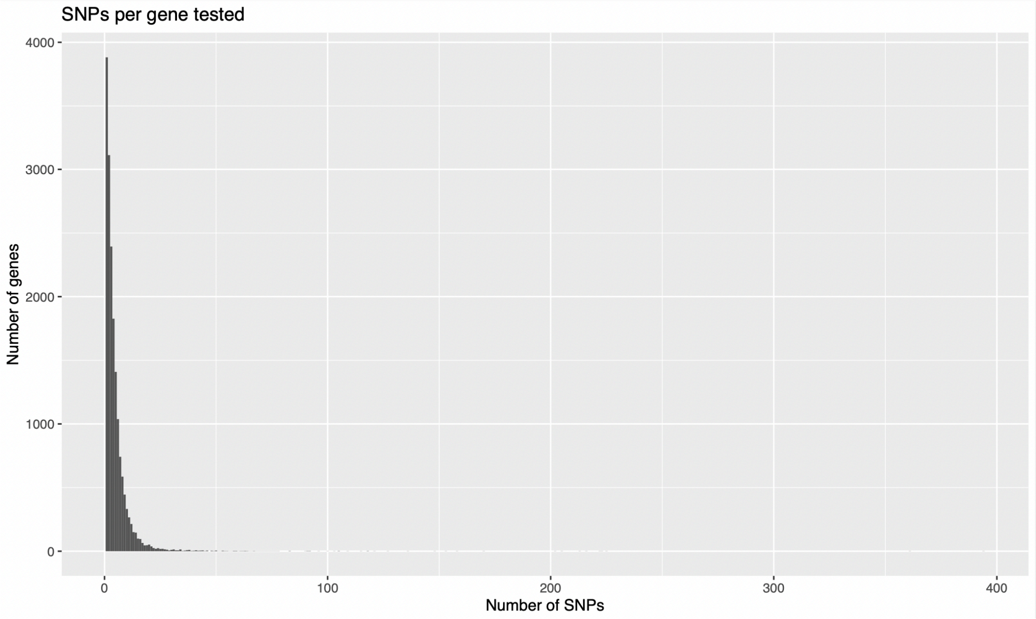

The number of SNPs per gene we tested in burden analyses is shown here as a histogram with a binwidth of 1. This shows a median of 3 SNPs per gene, a mode of 1 SNP per gene, and a maximum of 394 SNPs in the LDLR gene.

The genomic inflation factor was calculated using p-values from results of the burden analyses: 1.014893 for the internalising factor of depression, 1.044106 for the general factor of schizophrenia and 1.021464 for the general factor of bipolar disorder.

Figure S31. QQ plot depicting the expected versus observed –$\log_{10}$(p-value) of association test statistics for genes with predicted deleterious variants with (a) the internalising factor of depression (Int), (b) the general factor of schizophrenia (Sch), (c) the general factor of bipolar disorder (Bip)

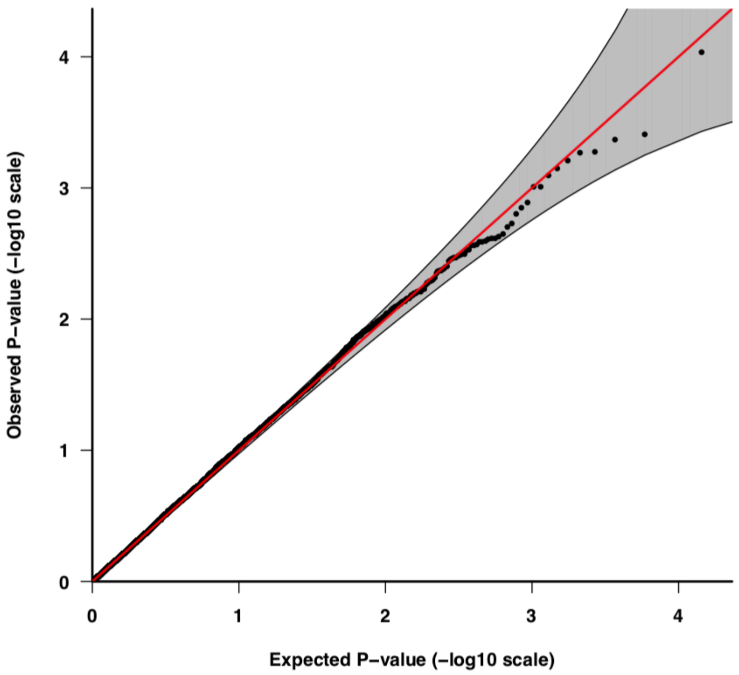

Observed P−value (−log10 scale)
Expected P−value (−log10 scale)
0
1
2
3
4

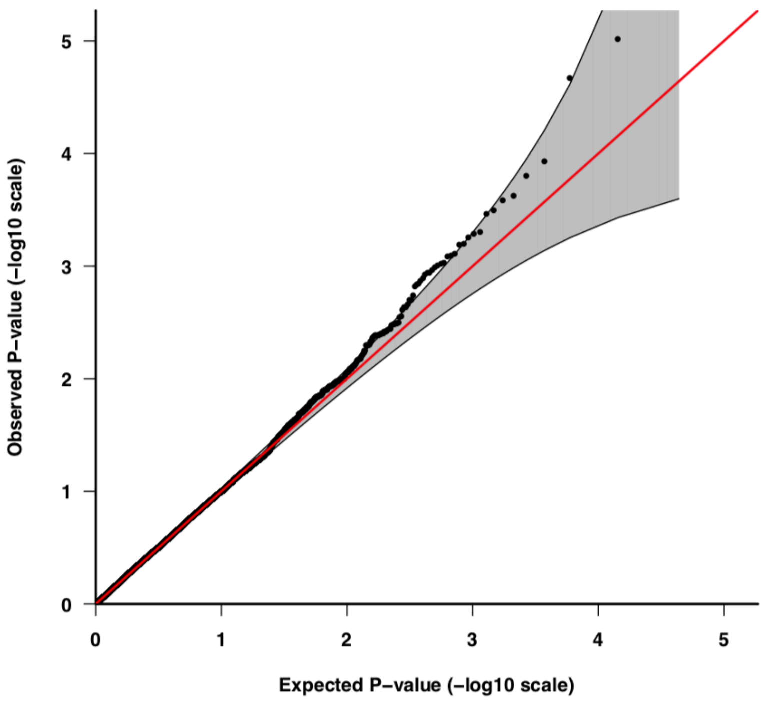

Observed P−value (−log10 scale)
Expected P−value (−log10 scale)
0
1
2
3
4
5

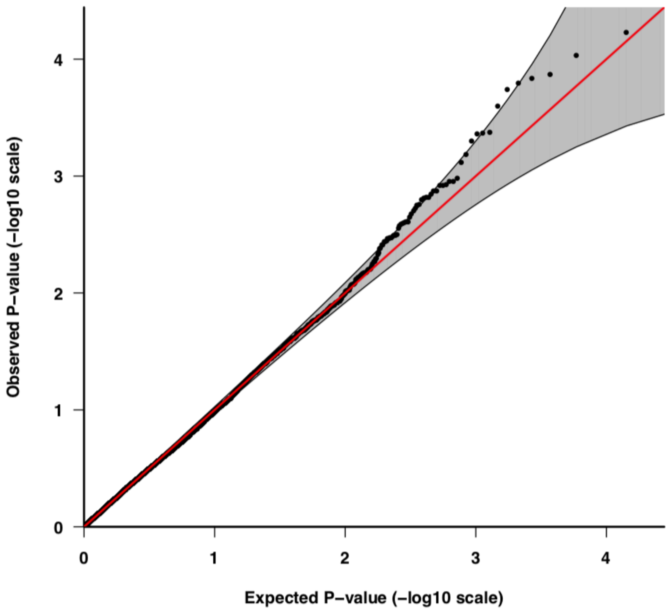

The QQ plot depicts the deviation of observed p-values from their distribution under a null hypothesis (shown as the red diagonal line). The p-values are plotted in descending order from left to right.

Table S17. Top 30 gene associations with the general internalising factor of depression

GENE	CHR	START	STOP	NSNPS	NRARE	N	ZSTAT	P	PERMP	NPERM	RSQ	RSQ_ADJ
ZNFX1	20	47862437	47894756	2	2	134463	3.7429	9.10E-05	0.001	1000	1.14E-04	1.06E-04
TRIM47	17	73870242	73874683	2	2	134463	3.3614	3.88E-04	0.001	1000	9.36E-05	8.62E-05
PIGT	20	44044707	44054885	2	2	134463	3.3372	4.23E-04	0.001	1000	9.24E-05	8.49E-05
NADSYN1	11	71164217	71212586	3	3	134463	3.2768	5.25E-04	0.001	1000	8.94E-05	8.20E-05
CCDC102B	18	66382466	66756517	4	4	134463	3.2731	5.32E-04	0.001	1000	8.92E-05	8.18E-05
FYB	5	39105354	39270759	1	1	134463	3.2336	6.11E-04	0.001	1000	8.73E-05	7.98E-05
CAMTA2	17	4871287	4890960	2	2	134463	3.1922	7.06E-04	0.001	1000	8.53E-05	7.78E-05
USP46	4	53457126	53525502	1	1	134463	3.1582	7.94E-04	0.003	1000	8.37E-05	7.62E-05
MTOR	1	11166588	11322614	1	1	134463	3.0985	9.73E-04	0.001	1000	8.09E-05	7.34E-05
ANGPTL7	1	11249346	11256038	1	1	134463	3.0985	9.73E-04	0.001	1000	8.09E-05	7.34E-05
GDAP2	1	118406107	118472302	2	2	134463	3.0158	0.0012815	0.002	1000	7.71E-05	6.97E-05
LAD1	1	201349966	201368669	1	1	134463	2.9883	0.0014025	0.002	1000	7.59E-05	6.84E-05
GALC	14	88304164	88460009	2	2	134463	2.9557	0.0015598	0.003	1000	7.44E-05	6.69E-05

ZDHHC16	10	99205900	99217127	2	2	134463	2.9028	0.0018494	0.003	1000	7.21E-05	6.46E-05
PCDHB8	5	140557371	140560081	1	1	134463	2.8812	0.0019806	0.001	1000	7.12E-05	6.37E-05
RFX3	9	3218297	3525992	1	1	134463	2.8457	0.0022159	0.001	1000	6.96E-05	6.22E-05
ID1	20	30193086	30194318	1	1	134463	2.8293	0.0023324	0.001	1000	6.89E-05	6.15E-05
RNFT1	17	58029723	58042117	2	2	134463	2.8186	0.0024115	0.001	1000	6.85E-05	6.10E-05
HCN3	1	155247218	155259639	5	5	134463	2.8186	0.002412	0.003	1000	6.85E-05	6.10E-05
CKAP5	11	46765084	46867859	5	5	134463	2.8145	0.002443	0.003	1000	6.83E-05	6.09E-05
CYB561D2	3	50388265	50391500	2	2	134463	2.8059	0.0025086	0.004	1000	6.79E-05	6.06E-05
ATRN	20	3451665	3631769	1	1	134463	2.7993	0.002561	0.003	1000	6.76E-05	6.02E-05
OR13C3	9	107298051	107299094	1	1	134463	2.7992	0.0025613	0.007	1000	6.76E-05	6.02E-05
GABRG1	4	46037786	46126082	5	5	134463	2.7839	0.0026858	0.002	1000	6.70E-05	5.96E-05
LACTBL1	1	23279536	23299524	4	4	134463	2.779	0.0027262	0.001	1000	6.68E-05	5.94E-05
FAM187B	19	35715704	35719628	1	1	134463	2.7786	0.0027297	0.004	1000	6.68E-05	5.94E-05
LMBR1L	12	49490334	49504683	2	2	134463	2.7576	0.0029116	0.005	1000	6.59E-05	5.85E-05
KLK7	19	51479735	51487320	2	2	134463	2.7523	0.002959	0.001	1000	6.57E-05	5.82E-05
RIIAD1	1	151694013	151702082	1	1	134463	2.7311	0.0031561	0.003	1000	6.48E-05	5.74E-05
ZNF334	20	45128269	45142198	1	1	134463	2.7296	0.0031706	0.003	1000	6.48E-05	5.73E-05

Table S18. Top 30 gene associations with the general factor of schizophrenia

GENE	CHR	START	STOP	NSNPS	NRARE	N	ZSTAT	P	PERMP	NPERM	RSQ	RSQ_ADJ
C19orf44	19	16607187	16632180	1	1	130013	4.2751	9.55E-06	0.001	1000	1.51E-04	1.43E-04
MRPL36	5	1798499	1799956	1	1	130013	4.0934	2.13E-05	0.001	1000	1.39E-04	1.31E-04
CAPN7	3	15247733	15294423	2	2	130013	3.6802	1.17E-04	0.001	1000	1.14E-04	1.06E-04
ZDHHC16	10	99205900	99217127	2	2	130013	3.605	1.56E-04	0.001	1000	1.10E-04	1.02E-04
ZPR1	11	116649276	116658739	1	1	130013	3.4958	2.36E-04	0.001	1000	1.04E-04	9.63E-05
COBL	7	51083909	51384515	4	4	130013	3.4747	2.56E-04	0.001	1000	1.03E-04	9.51E-05

PKIB	6	122793062	123047518	1	1	130013	3.4177	3.16E-04	0.001	1000	9.98E-05	9.21E-05
IFNA21	9	21165636	21166659	1	1	130013	3.3984	3.39E-04	0.002	1000	9.88E-05	9.11E-05
SS18L1	20	60718816	60757566	2	2	130013	3.2964	4.90E-04	0.002	1000	9.35E-05	8.58E-05
DBI	2	120124504	120130122	2	2	130013	3.2838	5.12E-04	0.001	1000	9.28E-05	8.52E-05
TMEM221	19	17546318	17559376	1	1	130013	3.2618	5.53E-04	0.003	1000	9.17E-05	8.40E-05
FAM200A	7	99143923	99156115	1	1	130013	3.224	6.32E-04	0.001	1000	8.98E-05	8.21E-05
CCRL2	3	46448721	46454488	1	1	130013	3.2208	6.39E-04	0.002	1000	8.97E-05	8.20E-05
CYB561D2	3	50388265	50391500	2	2	130013	3.1685	7.66E-04	0.001	1000	8.71E-05	7.94E-05
KLK7	19	51479735	51487320	2	2	130013	3.1549	8.03E-04	0.001	1000	8.64E-05	7.87E-05
C20orf141	20	2795633	2796476	2	2	130013	3.1499	8.17E-04	0.001	1000	8.62E-05	7.85E-05
SLC10A5	8	82605891	82607207	1	1	130013	3.1144	9.22E-04	0.002	1000	8.44E-05	7.67E-05
MPV17	2	27532360	27545969	1	1	130013	3.1058	9.49E-04	0.002	1000	8.40E-05	7.63E-05
TMEM134	11	67231819	67236748	1	1	130013	3.0987	9.72E-04	0.001	1000	8.37E-05	7.60E-05
NTRK3	15	88419948	88800026	4	4	130013	3.0869	0.0010114	0.001	1000	8.31E-05	7.54E-05
DHRS7	14	60611496	60633034	1	1	130013	3.0669	0.0010813	0.001	1000	8.22E-05	7.44E-05
MTOR	1	11166588	11322614	1	1	130013	3.0539	0.0011295	0.003	1000	8.15E-05	7.39E-05
ANGPTL7	1	11249346	11256038	1	1	130013	3.0539	0.0011295	0.003	1000	8.15E-05	7.39E-05
SORBS2	4	186506598	186877870	3	3	130013	3.0397	0.0011841	0.001	1000	8.09E-05	7.32E-05
NIPAL2	8	99202054	99307444	4	4	130013	3.0188	0.001269	0.001	1000	7.99E-05	7.22E-05
VPS53	17	411908	618096	1	1	130013	3.0015	0.0013433	0.003	1000	7.91E-05	7.14E-05
MAPK6	15	52296377	52358462	1	1	130013	2.9867	0.0014101	0.003	1000	7.84E-05	7.08E-05
UCP3	11	73711326	73720282	4	4	130013	2.9802	0.0014404	0.003	1000	7.81E-05	7.05E-05
ACRBP	12	6747241	6756594	2	2	130013	2.9667	0.0015052	0.004	1000	7.75E-05	6.97E-05
ZC3H12A	1	37940119	37949978	3	3	130013	2.9106	0.0018036	0.001	1000	7.49E-05	6.72E-05

Table S19. Top 30 gene associations with the general factor of bipolar disorder

GENE	CHR	START	STOP	NSNPS	NRARE	N	ZSTAT	P	PERMP	NPERM	RSQ	RSQ_ADJ
CCRL2	3	46448721	46454488	1	1	117376	3.8517	5.86E-05	0.001	1000	1.38E-04	1.29E-04
USP7	16	8985951	9057341	1	1	117376	3.7384	9.26E-05	0.001	1000	1.30E-04	1.22E-04
LRRC6	8	133584201	133687863	1	1	117376	3.6446	1.34E-04	0.001	1000	1.24E-04	1.16E-04
ZNF705G	8	7215498	7220490	1	1	117376	3.6252	1.44E-04	0.001	1000	1.23E-04	1.15E-04
SS18L1	20	60718816	60757566	2	2	117376	3.5982	1.60E-04	0.001	1000	1.21E-04	1.13E-04
C12orf56	12	64660763	64784345	5	5	117376	3.5657	1.81E-04	0.001	1000	1.19E-04	1.11E-04
C20orf141	20	2795633	2796476	2	2	117376	3.4786	2.52E-04	0.001	1000	1.14E-04	1.06E-04
KLK7	19	51479735	51487320	2	2	117376	3.3396	4.19E-04	0.001	1000	1.06E-04	9.75E-05
ZPR1	11	116649276	116658739	1	1	117376	3.3374	4.23E-04	0.003	1000	1.06E-04	9.74E-05
COBL	7	51083909	51384515	4	4	117376	3.3326	4.30E-04	0.002	1000	1.06E-04	9.71E-05
USP46	4	53457126	53525502	1	1	117376	3.293	4.96E-04	0.001	1000	1.03E-04	9.48E-05
ROPN1L	5	10441974	10465138	1	1	117376	3.217	6.48E-04	0.001	1000	9.91E-05	9.06E-05
SWSAP1	19	11485383	11487627	1	1	117376	3.1707	7.60E-04	0.001	1000	9.66E-05	8.80E-05
NT5DC2	3	52558385	52569093	1	1	117376	3.0808	0.0010323	0.001	1000	9.17E-05	8.32E-05
NTRK3	15	88419948	88800026	4	4	117376	3.0626	0.0010972	0.001	1000	9.08E-05	8.23E-05
SLC22A7	6	43265737	43273276	3	3	117376	3.0598	0.0011074	0.002	1000	9.06E-05	8.21E-05
MPV17	2	27532360	27545969	1	1	117376	3.0432	0.0011704	0.003	1000	8.98E-05	8.12E-05
PKIB	6	122793062	123047518	1	1	117376	3.0392	0.001186	0.002	1000	8.96E-05	8.11E-05
RAF1	3	12625100	12705700	5	5	117376	3.0368	0.0011954	0.001	1000	8.94E-05	8.09E-05
WEE1	11	9595197	9615016	1	1	117376	3.0064	0.0013218	0.002	1000	8.79E-05	7.93E-05
LIMA1	12	50569563	50677353	2	2	117376	3.0038	0.0013329	0.001	1000	8.77E-05	7.92E-05
MRPL36	5	1798499	1799956	1	1	117376	2.9848	0.0014188	0.004	1000	8.67E-05	7.82E-05
FAM162A	3	122103023	122128961	1	1	117376	2.9664	0.0015067	0.001	1000	8.58E-05	7.72E-05

TEP1	14	20833826	20882331	13	13	117376	2.9662	0.0015077	0.002	1000	8.58E-05	7.72E-05
MAPK6	15	52296377	52358462	1	1	117376	2.9612	0.0015324	0.002	1000	8.55E-05	7.70E-05
LRRC57	15	42834720	42841002	1	1	117376	2.9495	0.0015913	0.004	1000	8.49E-05	7.64E-05
ZDHHC16	10	99205900	99217127	2	2	117376	2.9237	0.0017297	0.002	1000	8.36E-05	7.51E-05
KCNB2	8	73449626	73850584	1	1	117376	2.9176	0.0017636	0.003	1000	8.33E-05	7.48E-05
USP20	9	132597696	132644121	3	3	117376	2.8983	0.0018757	0.004	1000	8.24E-05	7.38E-05
ATG4C	1	63249777	63330941	5	5	117376	2.8818	0.0019768	0.002	1000	8.15E-05	7.30E-05

Table S20. Top 30 GO pathway associations with the general internalising factor

FULL_NAME	TYPE	NGENES	BETA	BETA_STD	SE	P
GO CYCLIC NUCLEOTIDE PHOSPHODIESTERASE ACTIVITY	SET	13	1.1025	0.039801	0.28003	4.15E-05
GO CYCLIC NUCLEOTIDE CATABOLIC PROCESS	SET	8	1.228	0.034783	0.35695	2.92E-04
GO 3 5 CYCLIC AMP PHOSPHODIESTERASE ACTIVITY	SET	7	1.2261	0.032488	0.38161	6.59E-04
GO 3 5 CYCLIC GMP PHOSPHODIESTERASE ACTIVITY	SET	10	1.0021	0.031732	0.31933	8.53E-04
GO ORGAN GROWTH	SET	32	0.55822	0.031586	0.18038	9.88E-04
GO CYCLIC NUCLEOTIDE BINDING	SET	20	0.67443	0.030187	0.2259	0.0014191
GO RIBONUCLEOTIDE CATABOLIC PROCESS	SET	15	0.77733	0.030139	0.26079	0.0014414
GO TISSUE DEVELOPMENT	SET	827	0.10948	0.030206	0.036733	0.0014423
GO FEAR RESPONSE	SET	16	0.74174	0.029701	0.25254	0.0016605
GO DEVELOPMENTAL GROWTH	SET	182	0.22241	0.029784	0.075949	0.0017078
GO POSITIVE REGULATION OF GLUCONEOGENESIS	SET	6	1.1996	0.029429	0.41214	0.0018077

GO RESPONSE TO INTERLEUKIN 6	SET	17	0.72769	0.030034	0.25056	0.0018446
GO REGULATION OF BLOOD CIRCULATION	SET	168	0.22753	0.029296	0.078424	0.0018625
GO POSITIVE REGULATION OF GLIOGENESIS	SET	27	0.56596	0.029423	0.19642	0.001984
GO CYCLIC NUCLEOTIDE METABOLIC PROCESS	SET	29	0.53624	0.028889	0.18742	0.0021144
GO POSITIVE REGULATION OF GLIAL CELL DIFFERENTIATION	SET	18	0.68785	0.029211	0.24164	0.0022145
GO ANATOMICAL STRUCTURE FORMATION INVOLVED IN MORPHOGENESIS	SET	566	0.12487	0.028904	0.043943	0.0022494
GO CAMP METABOLIC PROCESS	SET	19	0.65663	0.028647	0.23128	0.0022671
GO SPLEEN DEVELOPMENT	SET	17	0.69162	0.028545	0.24502	0.0023854
GO SULFATION	SET	9	0.93174	0.027991	0.33378	0.0026284
GO CELL CELL SIGNALING	SET	376	0.14541	0.02771	0.052823	0.0029596
GO POSITIVE REGULATION OF DENDRITE MORPHOGENESIS	SET	19	0.63672	0.027779	0.23146	0.0029769
GO POSITIVE REGULATION OF CELL MATRIX ADHESION	SET	21	0.59658	0.027361	0.21968	0.0033133
GO ENDODERM FORMATION	SET	24	0.55535	0.027224	0.2063	0.0035569
GO POSITIVE REGULATION OF BLOOD CIRCULATION	SET	52	0.37371	0.026928	0.13889	0.0035725
GO STARTLE RESPONSE	SET	14	0.71818	0.026903	0.26838	0.0037315
GO REGULATION OF GLIOGENESIS	SET	48	0.39537	0.027377	0.14799	0.0037802
GO TRANSCRIPTIONAL ACTIVATOR ACTIVITY RNA POLYMERASE II TRANSCRIPTION	SET	133	0.23651	0.027143	0.089075	0.0039698

REGULATORY REGION SEQUENCE SPECIFIC BINDING						
GO SODIUM AMINO ACID SYMPORTER ACTIVITY	SET	8	0.90469	0.025626	0.3438	0.0042582
GO RECEPTOR INHIBITOR ACTIVITY	SET	6	1.0783	0.026454	0.41223	0.0044577

Table S21. Top 30 GO pathway associations with the general factor of schizophrenia

FULL_NAME	TYPE	NGENES	BETA	BETA_STD	SE	P
GO CELLULAR RESPONSE TO LIPID	SET	232	0.24861	0.037511	0.067069	1.06E-04
GO POSITIVE REGULATION OF GROWTH	SET	115	0.33693	0.036007	0.094037	1.71E-04
GO CALCIUM ION TRANSPORT	SET	137	0.30131	0.035106	0.086101	2.34E-04
GO CIRCADIAN RHYTHM	SET	69	0.4131	0.034276	0.12079	3.14E-04
GO ORGANIC ACID TRANSMEMBRANE TRANSPORT	SET	62	0.42055	0.033089	0.124	3.49E-04
GO DIVALENT INORGANIC CATION TRANSPORT	SET	163	0.26727	0.033922	0.079056	3.63E-04
GO POSITIVE REGULATION OF CELL GROWTH	SET	65	0.4144	0.033379	0.12415	4.24E-04
GO CELLULAR RESPONSE TO FATTY ACID	SET	32	0.59467	0.033665	0.17945	4.62E-04
GO REGULATION OF INTERFERON GAMMA PRODUCTION	SET	39	0.53884	0.033663	0.16449	5.29E-04
GO POSITIVE REGULATION OF PEPTIDYL TYROSINE PHOSPHORYLATION	SET	75	0.38515	0.033308	0.11766	5.33E-04
GO POSITIVE REGULATION OF MULTICELLULAR ORGANISMAL PROCESS	SET	734	0.12387	0.032374	0.038488	6.47E-04

GO TRANSFORMING GROWTH FACTOR BETA RECEPTOR BINDING	SET	24	0.67286	0.033001	0.21151	7.35E-04
GO DNA DIRECTED RNA POLYMERASE II CORE COMPLEX	SET	4	1.5835	0.031738	0.50113	7.92E-04
GO POSITIVE REGULATION OF NEURON DIFFERENTIATION	SET	165	0.24815	0.031685	0.078931	8.36E-04
GO CELLULAR RESPONSE TO CYTOKINE STIMULUS	SET	285	0.18832	0.031407	0.060473	9.25E-04
GO INNER CELL MASS CELL PROLIFERATION	SET	7	1.1708	0.031039	0.37889	0.0010034
GO CELLULAR RESPONSE TO ORGANIC SUBSTANCE	SET	934	0.10607	0.03093	0.034645	0.0011038
GO POSITIVE REGULATION OF DEVELOPMENTAL GROWTH	SET	82	0.34292	0.030998	0.11213	0.0011165
GO REGULATION OF CELL GROWTH	SET	200	0.21428	0.030069	0.070763	0.0012333
GO HEART VALVE DEVELOPMENT	SET	22	0.64632	0.030353	0.21763	0.0014934
GO POSITIVE REGULATION OF CELL DEVELOPMENT	SET	258	0.1866	0.029651	0.063228	0.0015862
GO NEGATIVE REGULATION OF TRANSCRIPTION REGULATORY REGION DNA BINDING	SET	9	0.98154	0.029502	0.3342	0.0016609
GO REGULATION OF CAMP DEPENDENT PROTEIN KINASE ACTIVITY	SET	8	1.0342	0.02931	0.3543	0.0017594
GO ATRIOVENTRICULAR	SET	14	0.78099	0.02927	0.26802	0.0017885

VALVE DEVELOPMENT						
GO MODIFICATION OF MORPHOLOGY OR PHYSIOLOGY OF OTHER ORGANISM	SET	51	0.40702	0.029061	0.13994	0.0018195
GO RESPONSE TO LIPID	SET	462	0.13859	0.029157	0.047961	0.0019332
GO QUATERNARY AMMONIUM GROUP TRANSPORT	SET	15	0.74659	0.028962	0.25844	0.0019378
GO MITOTIC SPINDLE	SET	27	0.53777	0.027971	0.18696	0.002015
GO POSITIVE REGULATION OF EXCITATORY POSTSYNAPTIC POTENTIAL	SET	12	0.81167	0.028166	0.28948	0.0025295
GO ION TRANSMEMBRANE TRANSPORT	SET	500	0.12758	0.027868	0.045522	0.0025392

Table S22. Top 30 GO pathway associations with the general factor of bipolar disorder

FULL_NAME	TYPE	NGENES	BETA	BETA_STD	SE	P
GO INTERACTION WITH HOST	SET	75	0.43334	0.037553	0.11429	5.30E-06
GO MODIFICATION BY SYMBIONT OF HOST MORPHOLOGY OR PHYSIOLOGY	SET	29	0.73534	0.039719	0.18395	3.22E-05
GO REGULATION OF TRANSCRIPTION INVOLVED IN G1 S TRANSITION OF MITOTIC CELL CYCLE	SET	11	1.1419	0.038022	0.30824	1.06E-04
GO REGULATION OF PROTEIN STABILITY	SET	110	0.31778	0.033292	0.094738	3.99E-04
GO INTERSPECIES INTERACTION BETWEEN ORGANISMS	SET	311	0.1861	0.032444	0.056758	5.23E-04

GO SPROUTING ANGIOGENESIS	SET	26	0.64079	0.032777	0.19957	6.64E-04
GO PROTEIN STABILIZATION	SET	73	0.36789	0.031457	0.11618	7.74E-04
GO REGULATION OF TELOMERE MAINTENANCE	SET	35	0.50774	0.03012	0.16361	9.59E-04
GO CARDIOLIPIN METABOLIC PROCESS	SET	7	1.1498	0.030546	0.37304	0.00103
GO MODULATION BY VIRUS OF HOST MORPHOLOGY OR PHYSIOLOGY	SET	23	0.63613	0.030609	0.20671	0.0010471
GO CELLULAR RESPONSE TO INTERLEUKIN 4	SET	10	0.95428	0.030297	0.31215	0.0011204
GO RESPONSE TO GROWTH FACTOR	SET	234	0.2021	0.030685	0.066145	0.0011266
GO MODIFICATION OF MORPHOLOGY OR PHYSIOLOGY OF OTHER ORGANISM	SET	51	0.42055	0.03009	0.13796	0.0011538
GO RESPONSE TO BMP	SET	55	0.40723	0.030252	0.13503	0.0012844
GO TRANSMEMBRANE RECEPTOR PROTEIN SERINE THREONINE KINASE SIGNALING PATHWAY	SET	101	0.29673	0.029802	0.10008	0.001517
GO PROTEIN BINDING INVOLVED IN PROTEIN FOLDING	SET	7	1.0187	0.027064	0.34594	0.0016195
GO HYDROLASE ACTIVITY ACTING ON ETHER BONDS	SET	8	1.012	0.028741	0.3488	0.0018615
GO ACUTE PHASE RESPONSE	SET	20	0.64049	0.028743	0.22301	0.0020435
GO REGULATION OF CHROMATIN SILENCING	SET	8	0.99898	0.028371	0.34886	0.0020989
GO POSITIVE REGULATION OF	SET	33	0.48236	0.027787	0.16857	0.0021124

DNA BIOSYNTHETIC PROCESS						
GO CONDENSED NUCLEAR CHROMOSOME CENTROMERIC REGION	SET	8	0.99847	0.028356	0.34898	0.0021155
GO POSITIVE REGULATION OF TELOMERASE ACTIVITY	SET	17	0.65035	0.026912	0.23172	0.0025081
GO CYTOKINE BINDING	SET	50	0.38878	0.027545	0.13977	0.0027099
GO NEGATIVE REGULATION OF MULTI ORGANISM PROCESS	SET	79	0.30692	0.027292	0.11096	0.0028424
GO CYSTEINE TYPE ENDOPEPTIDASE ACTIVITY	SET	50	0.38197	0.027062	0.13842	0.0029
GO CELL CYCLE DNA REPLICATION	SET	5	1.2153	0.027289	0.44137	0.0029547
GO POSITIVE REGULATION OF TELOMERE MAINTENANCE VIA TELOMERE LENGTHENING	SET	20	0.58801	0.026388	0.2147	0.0030894
GO LOW DENSITY LIPOPROTEIN RECEPTOR ACTIVITY	SET	11	0.81379	0.027096	0.29765	0.003134
GO TRANSITION METAL ION TRANSPORT	SET	58	0.35567	0.027128	0.13057	0.0032315
GO REGULATION OF MULTI ORGANISM PROCESS	SET	237	0.17611	0.026906	0.06515	0.0034398

Heritability estimation and genetic correlations

We used the reported effective sample size (N_{eff}) in each manuscript, and for the latent factors we calculated these using the formula as mentioned in Mullins et al.[24]

N_{eff} = 4 * n_cases * n_controls / (n_cases + n_controls)

Table S22. Effective sample sizes used to estimate heritability of case-control traits using high-definition likelihood inference (HDL)

	N_{eff} (effective sample size)
Major depression (Wray et al. - excluding 23andMe and UK Biobank)	111,221
Bipolar disorder (Mullins et al.)	101,962
Schizophrenia (Ripke et al.)	157,013
Int factor	74,663
Sch factor	74,087
Bip factor	65,709

Figure S33. Point estimates and confidence intervals of heritability of latent traits and matched phenotypes using high-definition likelihood inference (HDL)

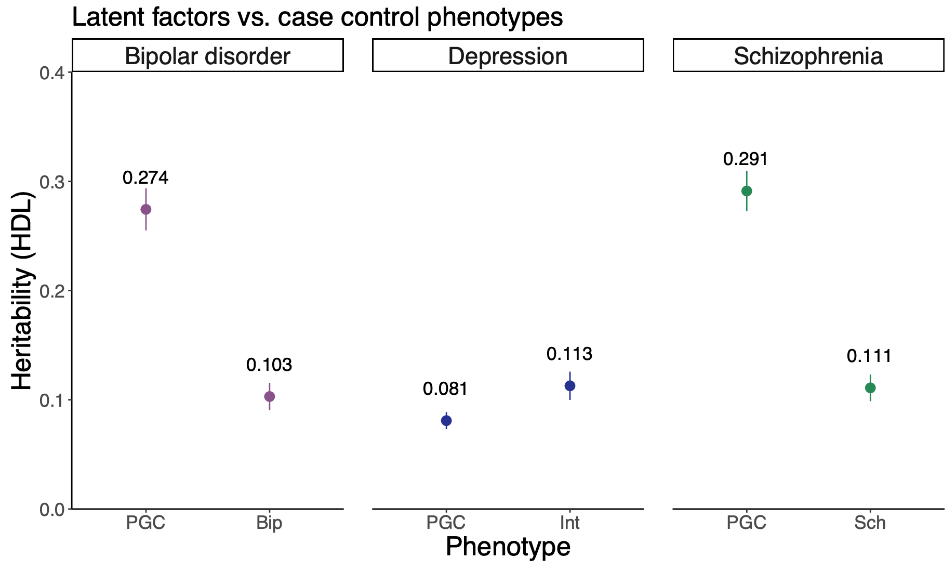

The vertical axis depicts the heritability (h^2) estimates of genetic data calculated using high-definition likelihood inference for latent traits in this study and matched GWAS phenotypes from summary

statistics in Wray et al.[47], Ripke et al.[25] and Mullins et al.[48] (noted as PGC papers). Points represent point estimates of heritability, while line ranges represent 95% confidence intervals.

Table S23. Table to show genetic correlations between latent psychiatric traits and case-control status from previous studies

Genetic correlations (SE)	Depression (Wray et al.)	Schizophrenia (Ripke et al.)	Bipolar disorder (Mullins et al.)
Internalising (Int)	**0.68 (0.05)**	0.21 (0.03)	0.20 (0.03)
General factor of schizophrenia (Sch)	0.70 (0.05)	**0.24 (0.03)**	0.27 (0.03)
General factor of bipolar disorder (Bip)	0.74 (0.05)	0.34 (0.03)	**0.40 (0.04)**

Results of genetic correlation analysis of latent traits in this study and case control analyses from matched GWAS studies, using the high-definition likelihood inference software (HDL). SE=standard error. For matched GWAS studies, we estimated heritability with HDL using summary statistics from the original papers. For Wray et al.,we used summary statistics from PGC excluding UK Biobank and 23andMe data.

Figure S34. Density plot depicting the distribution of nested window heritability, polygenicity and sigma for latent traits across 10 000 iterations of the model using the nested BayesC method in GCTB, using data from chromosome 1.

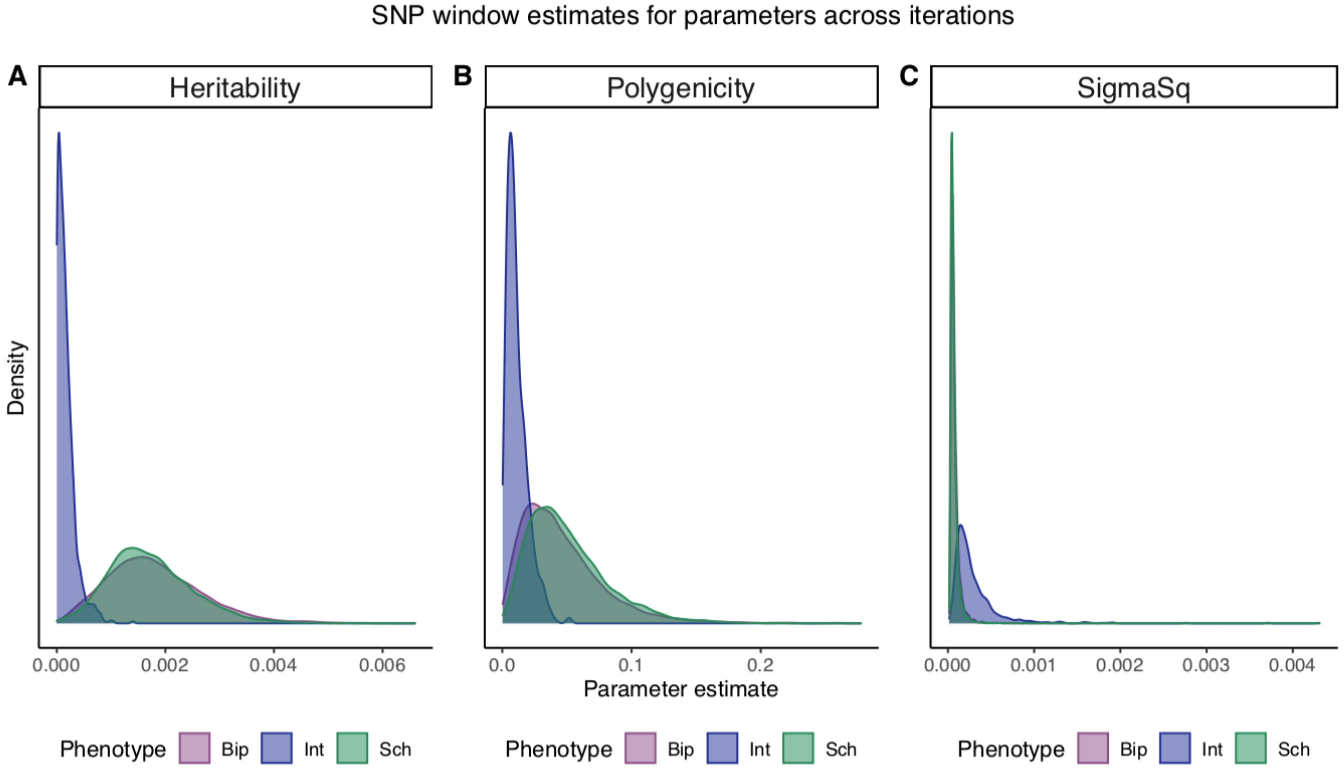

The horizontal axis depicts the heritability (h^2), polygenicity or sigma of 1 kB nested windows with the latent factors. We ran the MCMC model with 10 000 iterations, with a burn in of 2 000, using starting values of pi=0.1, h2=0.1 and S=0, and a MAF threshold of >0.005%. The vertical axis depicts the density of iterations with those estimated values. For computational feasibility, we conducted the analysis on data from chromosome 1 only; the distribution of S and pi are not expected to vary between chromosomes and are not additive. The MCMC model for the internalising factor of depression did not converge under the parameters we specified.

Table S24. Table to show point estimates and 95% credible intervals from GCTB estimation of heritability, polygenicity and selection using data from chromosome 1

Point estimates (95% credible intervals)	Heritability (h^2)	Polygenicity (pi)	Selection (sigma)
Internalising (Int)	NA	NA	NA
General factor of schizophrenia (Sch)	0.0018 (0.00038, 0.0035)	0.052 (0.0024, 0.11)	0.000068 (0.000020, 0.00015)
General factor of bipolar disorder (Bip)	0.0018 (0.00026, 0.0035)	0.045 (0.00091, 0.11)	0.000078 (0.00023, 0.00017)

The values describe the point estimates and 95% credible intervals of heritability (h^2), polygenicity or sigma of 1 kB nested windows with the latent factors. We ran the MCMC model with 10 000 iterations, with a burn in of 2 000, using starting values of pi=0.1, h2=0.1 and S=0, and a MAF threshold of >0.005%. For computational feasibility, we conducted the analysis on data from chromosome 1 only; the distribution of S and pi are not expected to vary between chromosomes and are not additive. The MCMC model for the internalising factor of depression did not converge under the parameters we specified.

References

1. Al Eissa, M. M. *et al.* Exome sequence analysis and follow up genotyping implicates rare *ULK1* variants to be involved in susceptibility to schizophrenia. *Annals of Human Genetics* **82**, 88–92 (2018).
2. Coelewij, L. & Curtis, D. Mini-review: Update on the genetics of schizophrenia. *Annals of Human Genetics* **82**, 239–243 (2018).
3. Giacopuzzi, E. *et al.* Exome sequencing in schizophrenic patients with high levels of homozygosity identifies novel and extremely rare mutations in the GABA/glutamatergic pathways. *PLoS ONE* **12**, e0182778 (2017).
4. Harold, D. *et al.* Population-based identity-by-descent mapping combined with exome sequencing to detect rare risk variants for schizophrenia. *Am. J. Med. Genet.* **180**, 223–231 (2019).
5. Lescai, F. *et al.* LARGE META-ANALYSIS OF SCANDINAVIAN EXOME SEQUENCING STUDIES OF SCHIZOPHRENIA. *European Neuropsychopharmacology* **29**, S813 (2019).
6. Rhoades, R., Jackson, F. & Teng, S. Discovery of rare variants implicated in schizophrenia using next-generation sequencing. *JTGG* (2019) doi:10.20517/jtgg.2018.26.
7. Swedish Schizophrenia Study *et al.* Rare loss-of-function variants in SETD1A are associated with schizophrenia and developmental disorders. *Nat Neurosci* **19**, 571–577 (2016).
8. Watanabe, Y. *et al.* Rare truncating variations and risk of schizophrenia: Whole-exome sequencing in three families with affected siblings and a three-stage follow-up study in a Japanese population. *Psychiatry Research* **235**, 13–18 (2016).
9. Zhao, L. *et al.* Replicated associations of FADS1, MAD1L1, and a rare variant at 10q26.13 with bipolar disorder in Chinese population. *Transl Psychiatry* **8**, 270 (2018).
10. Shim, H. *et al.* A Multivariate Genome-Wide Association Analysis of 10 LDL Subfractions, and Their Response to Statin Treatment, in 1868 Caucasians. *PLoS ONE* **10**, e0120758 (2015).
11. Cardon, L. R. & Abecasis, G. R. Some Properties of a Variance Components Model for Fine-Mapping Quantitative Trait Loci. *Behavior Genetics* **30**, 235–243 (2000).
12. Purcell, S., Cherny, S. S. & Sham, P. C. Genetic Power Calculator: design of linkage and association genetic mapping studies of complex traits. *Bioinformatics* **19**, 149–150 (2003).

13. Wu, Y., Zheng, Z., Visscher, P. M. & Yang, J. Quantifying the mapping precision of genome-wide association studies using whole-genome sequencing data. *Genome Biol* **18**, 86 (2017).
14. Steinberg, S. *et al.* Truncating mutations in RBM12 are associated with psychosis. *Nat Genet* **49**, 1251–1254 (2017).
15. GROUP Investigators *et al.* De novo mutations identified by exome sequencing implicate rare missense variants in SLC6A1 in schizophrenia. *Nat Neurosci* (2020) doi:10.1038/s41593-019-0565-2.
16. Derkach, A., Zhang, H. & Chatterjee, N. Power Analysis for Genetic Association Test (PAGEANT) provides insights to challenges for rare variant association studies. *Bioinformatics* **34**, 1506–1513 (2018).
17. de Leeuw, C. A., Mooij, J. M., Heskes, T. & Posthuma, D. MAGMA: Generalized Gene-Set Analysis of GWAS Data. *PLoS Comput Biol* **11**, e1004219 (2015).
18. Hubert, M., Debruyne, M. & Rousseeuw, P. J. Minimum covariance determinant and extensions. *WIREs Comput Stat* **10**, e1421 (2018).
19. Hubert, M. & Debruyne, M. Minimum covariance determinant: Minimum covariance determinant. *WIREs Comp Stat* **2**, 36–43 (2010).
20. Zeng, J. *et al.* Signatures of negative selection in the genetic architecture of human complex traits. *Nat Genet* **50**, 746–753 (2018).
21. Jermy, B. S. *et al.* Using major depression polygenic risk scores to explore the depressive symptom continuum. *Psychological Medicine* 1–10 (2020) doi:10.1017/S0033291720001828.
22. Beaton, D. *et al. Generalization of the minimum covariance determinant algorithm for categorical and mixed data types*. http://biorxiv.org/lookup/doi/10.1101/333005 (2018) doi:10.1101/333005.
23. Wray, N. R. *et al.* Genome-wide association analyses identify 44 risk variants and refine the genetic architecture of major depression. *Nature genetics* **50**, 668–681 (2018).
24. Mullins, N. *et al.* Genome-wide association study of more than 40,000 bipolar disorder cases provides new insights into the underlying biology. *Nat Genet* **53**, 817–829 (2021).
25. Schizophrenia Working Group of the Psychiatric Genomics Consortium, Ripke, S., Walters, J. T. & O'Donovan, M. C. *Mapping genomic loci prioritises genes and implicates synaptic biology in*

schizophrenia. http://medrxiv.org/lookup/doi/10.1101/2020.09.12.20192922 (2020) doi:10.1101/2020.09.12.20192922.

26. Bycroft, C. *et al. Genome-wide genetic data on ~500,000 UK Biobank participants*. http://biorxiv.org/lookup/doi/10.1101/166298 (2017) doi:10.1101/166298.
27. Affymetrix. UK Biobank Axiom Array DataSheet. (2014).
28. The 1000 Genomes Project Consortium. An integrated map of genetic variation from 1,092 human genomes. *Nature* **491**, 56–65 (2012).
29. Manichaikul, A. *et al.* Robust relationship inference in genome-wide association studies. *Bioinformatics* **26**, 2867–2873 (2010).
30. Choi, S. W. GreedyRelated. (2019).
31. Bycroft, C. *et al.* The UK Biobank resource with deep phenotyping and genomic data. *Nature* **562**, 203–209 (2018).
32. The 1000 Genomes Project Consortium. A global reference for human genetic variation. *Nature* **526**, 68–74 (2015).
33. UK10K Consortium *et al.* Improved imputation of low-frequency and rare variants using the UK10K haplotype reference panel. *Nat Commun* **6**, 8111 (2015).
34. McCarthy, D. J. *et al.* Choice of transcripts and software has a large effect on variant annotation. *Genome Med* **6**, 26 (2014).
35. Pistis, G. *et al.* Rare variant genotype imputation with thousands of study-specific whole-genome sequences: implications for cost-effective study designs. *Eur J Hum Genet* **23**, 975–983 (2015).
36. Wright, C. F. *et al.* Assessing the Pathogenicity, Penetrance, and Expressivity of Putative Disease-Causing Variants in a Population Setting. *The American Journal of Human Genetics* **104**, 275–286 (2019).
37. Aken, B. L. *et al.* The Ensembl gene annotation system. *Database* **2016**, baw093 (2016).
38. Liu, X., Wu, C., Li, C. & Boerwinkle, E. dbNSFP v3.0: A One-Stop Database of Functional Predictions and Annotations for Human Nonsynonymous and Splice-Site SNVs. *Human Mutation* **37**, 235–241 (2016).

39. Jian, X., Boerwinkle, E. & Liu, X. In silico prediction of splice-altering single nucleotide variants in the human genome. *Nucleic Acids Research* **42**, 13534–13544 (2014).

40. Schwarz, J. M., Rödelsperger, C., Schuelke, M. & Seelow, D. MutationTaster evaluates disease-causing potential of sequence alterations. *Nat Methods* **7**, 575–576 (2010).

41. Davydov, E. V. *et al.* Identifying a high fraction of the human genome to be under selective constraint using GERP++. *PLoS Comput. Biol.* **6**, e1001025 (2010).

42. Shihab, H. A. *et al.* Predicting the Functional, Molecular, and Phenotypic Consequences of Amino Acid Substitutions using Hidden Markov Models. *Human Mutation* **34**, 57–65 (2013).

43. Sim, N.-L. *et al.* SIFT web server: predicting effects of amino acid substitutions on proteins. *Nucleic Acids Research* **40**, W452–W457 (2012).

44. Habier, D., Fernando, R. L., Kizilkaya, K. & Garrick, D. J. Extension of the bayesian alphabet for genomic selection. *BMC Bioinformatics* **12**, 186 (2011).

45. Meuwissen, T. H., Hayes, B. J. & Goddard, M. E. Prediction of total genetic value using genome-wide dense marker maps. *Genetics* **157**, 1819–1829 (2001).

46. National Center for Biotechnology Information. NCBI Remap.

47. eQTLGen Consortium *et al.* Genome-wide association study identifies 30 loci associated with bipolar disorder. *Nat Genet* **51**, 793–803 (2019).

48. Mullins, N. *et al. Genome-wide association study of over 40,000 bipolar disorder cases provides novel biological insights*. http://medrxiv.org/lookup/doi/10.1101/2020.09.17.20187054 (2020) doi:10.1101/2020.09.17.20187054.
